# Supplementary figures and images for: XDream: Finding preferred stimuli for visual neurons using generative networks and gradient-free optimization
Source: PLoS Comput Biol. 2020 Jun 15;16(6):e1007973. doi: 10.1371/journal.pcbi.1007973 (PMC7316361; doi:10.1371/journal.pcbi.1007973)

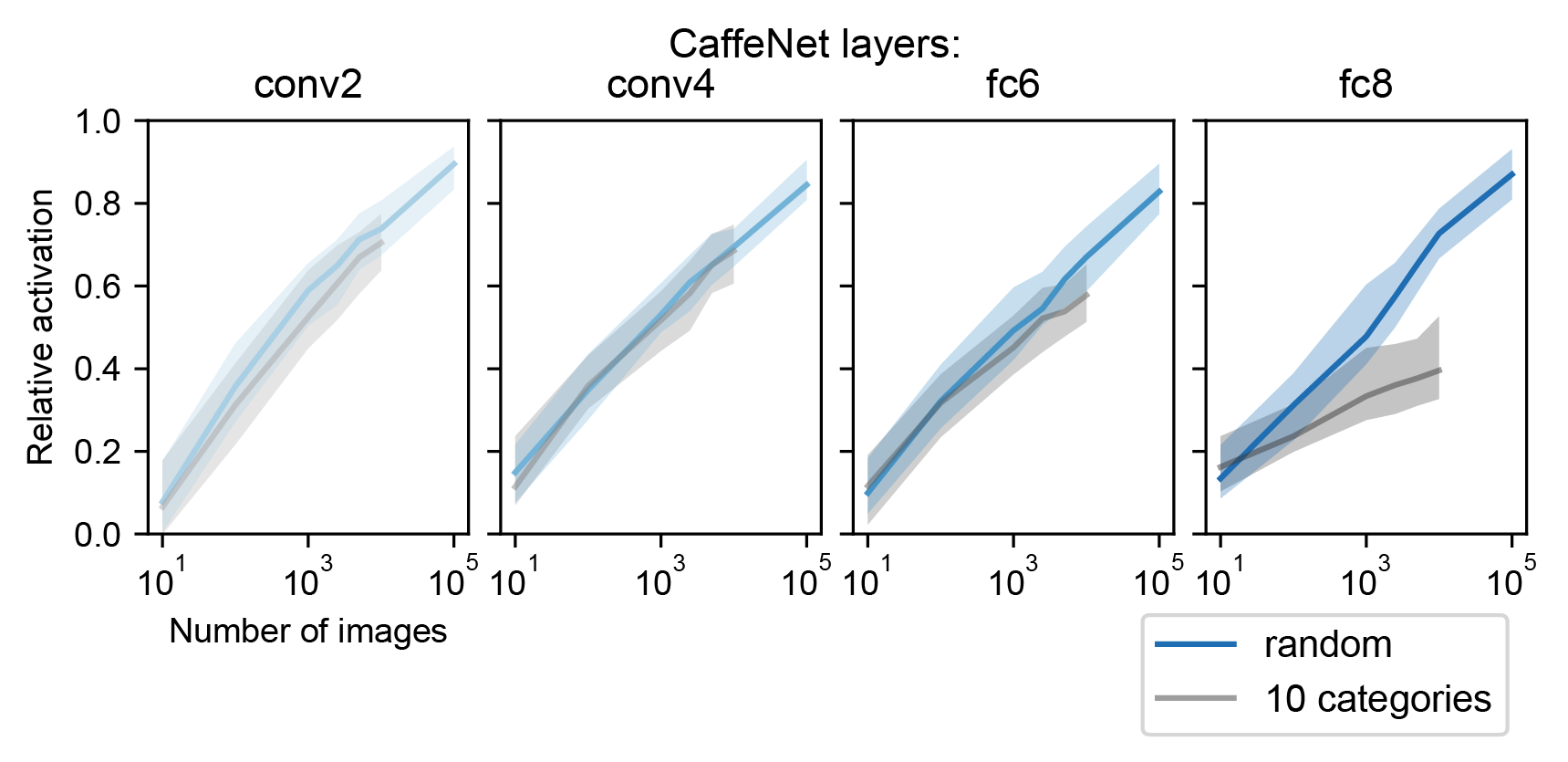

Supplement: S1 Fig — We measured the max relative activation expected in two random sampling schemes. “Random” refers to picking a given number of images randomly from the ImageNet dataset (blue). “10 categories” refers to first randomly picking 10 categories out of the 1000 ImageNet categories and then picking randomly from those categories (gray). We considered 4 layers from the CaffeNet architecture. Lines indicate the median relative activation (activation divided by the highest activation for all ImageNet images). Shading indicates the 25th- to 75th-percentiles among 100 random units per layer. (TIF) [file pcbi.1007973.s001.tif]

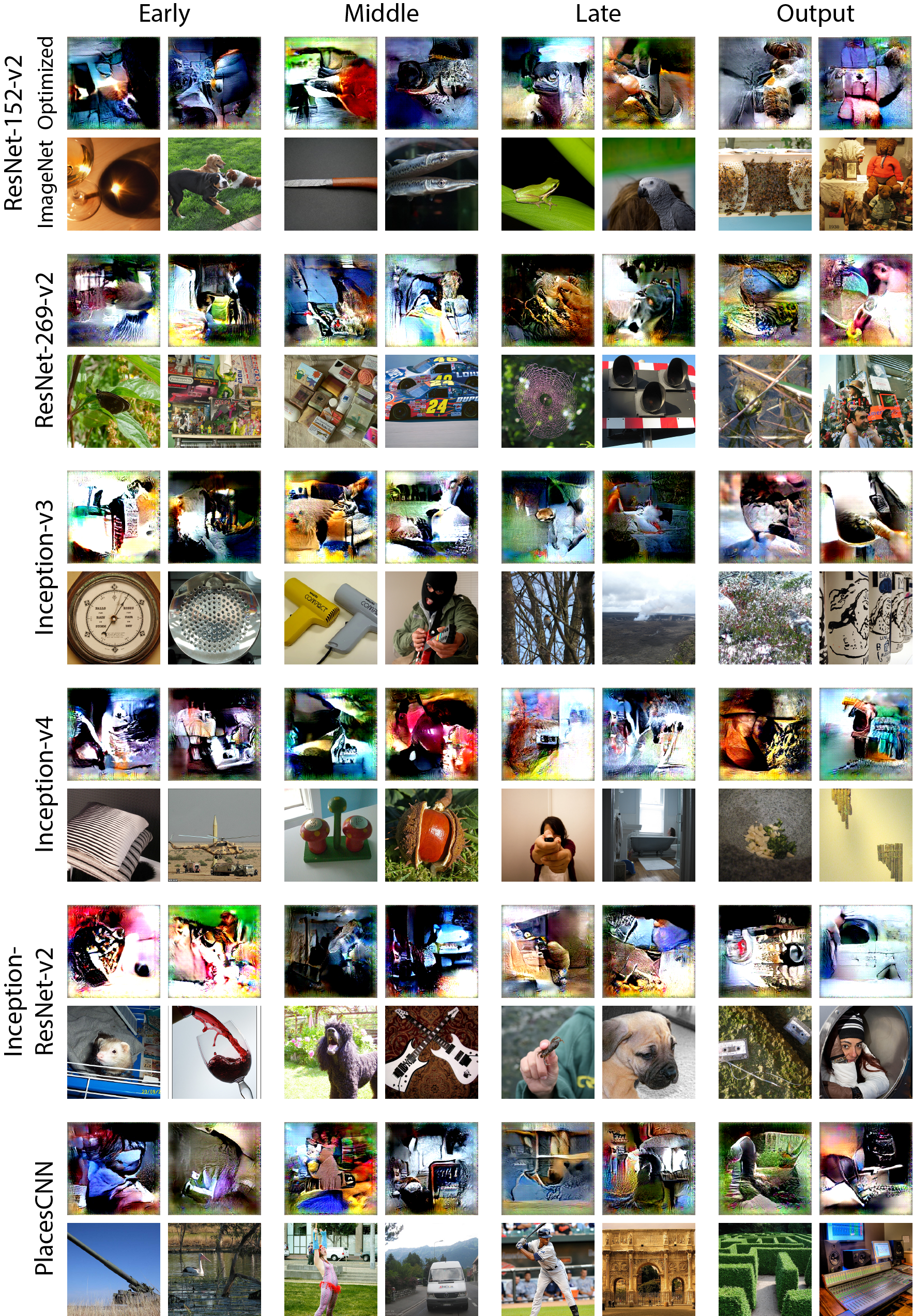

Supplement: S2 Fig — Two neurons were randomly selected per layer per architecture (S1 Table). Format is the same as in Fig 2. (TIF) [file pcbi.1007973.s002.tif]

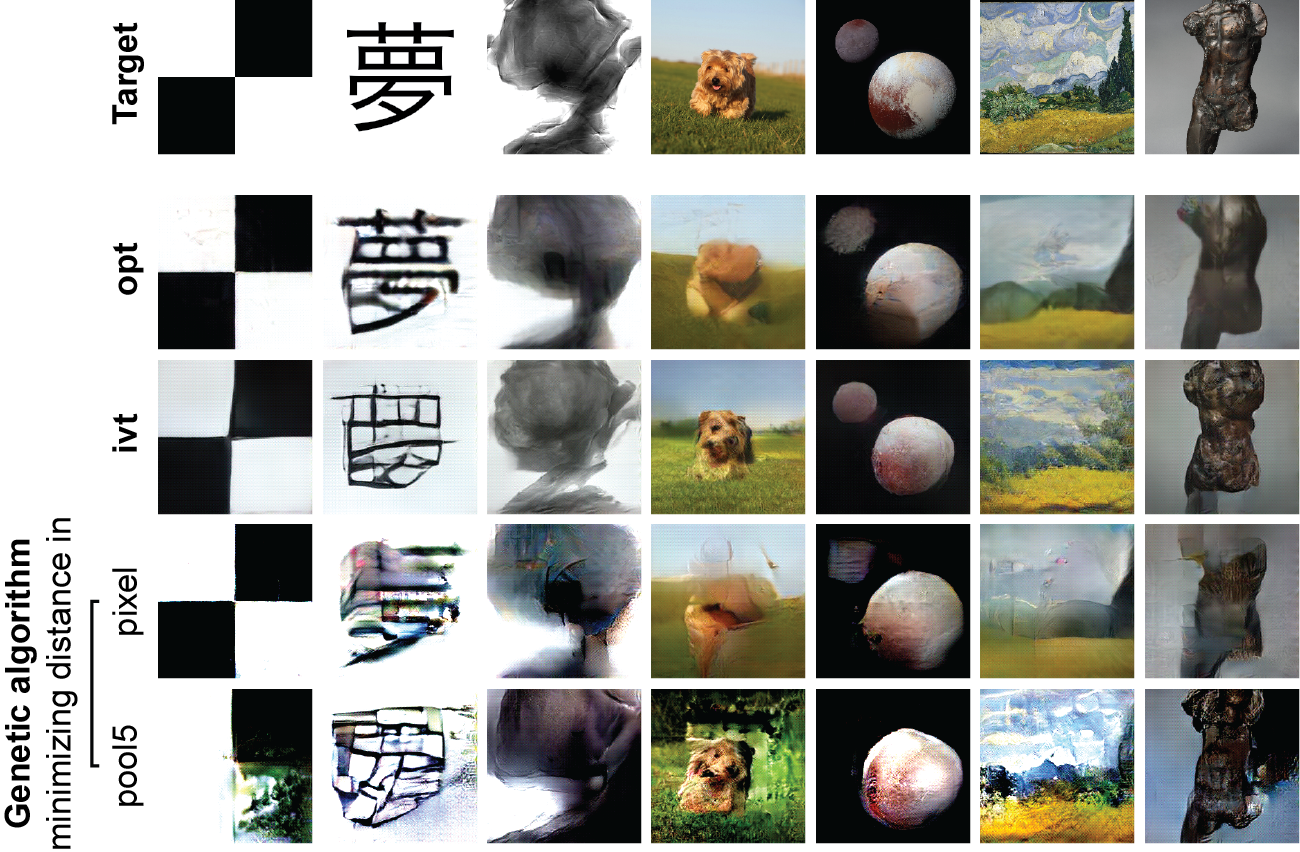

Supplement: S3 Fig — This figure reproduces Supplementary Figure 1 in [13]. The generative network is challenged to synthesize arbitrary target images (row 1) using one of two encoding methods, “opt” (row 2) and “ivt” (row 3; Methods). In addition, XDream can discover the target image efficiently (within 10,000 test image presentations) by using the genetic algorithm to minimize the mean squared difference between the target image and any test image as a loss function, either in pixel space (row 4) or in CaffeNet pool5 representation space (row 5). (TIF) [file pcbi.1007973.s003.tif]

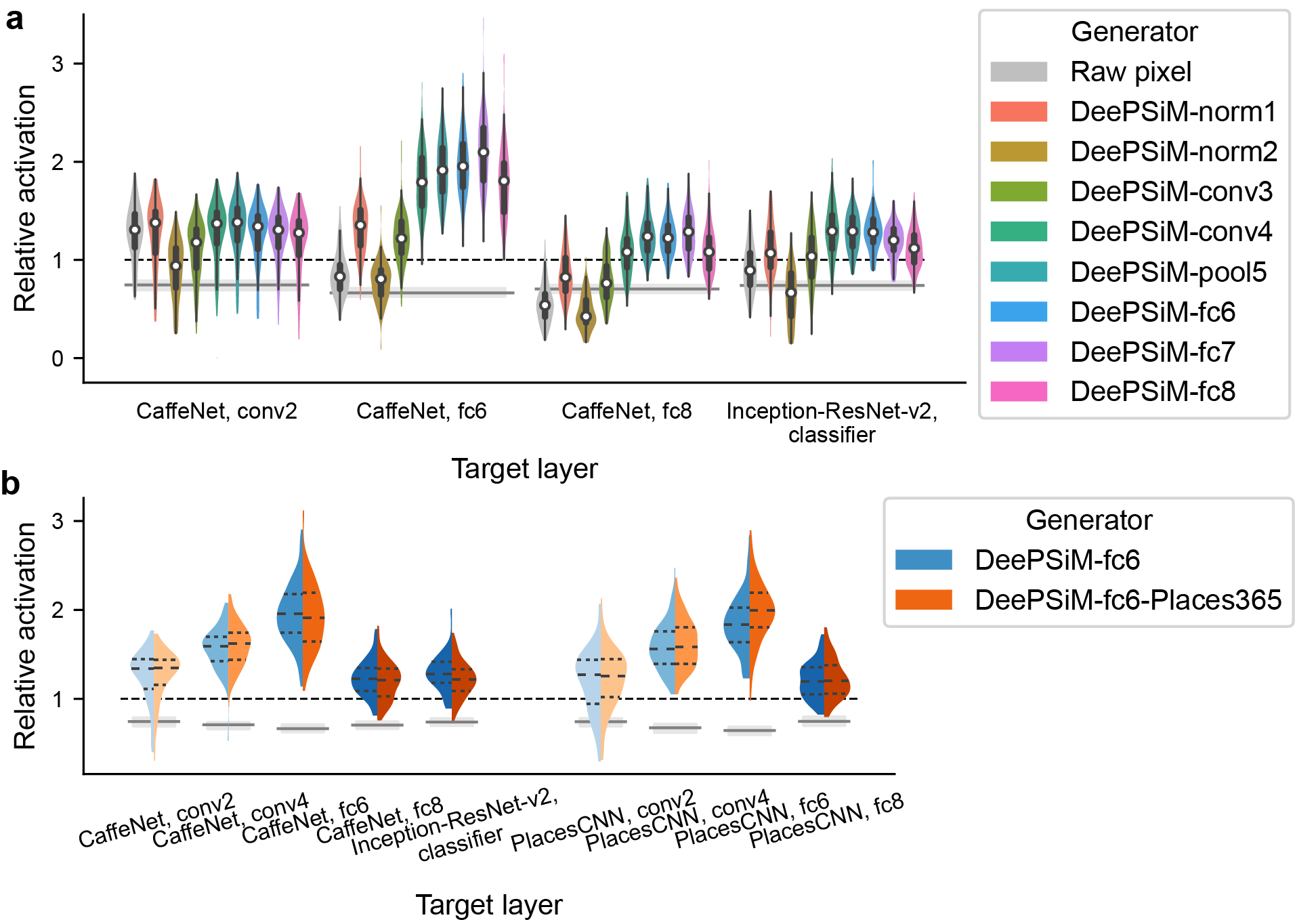

Supplement: S4 Fig — a) We tested each of the family of image generators from [8] as the image generator in XDream, together with a generator directly representing images as pixels. Format of the plot is the same as in Fig 2a. b), The same generator architecture (DeePSiM-fc6) was trained on ImageNet and Places365, respectively, and tested on classifiers trained on either dataset. Each half of a violin corresponds to one generator, and dashed lines inside the violins indicate quartiles of the distribution; otherwise, format of the plot is the same as in Fig 2a. (TIF) [file pcbi.1007973.s004.tif]

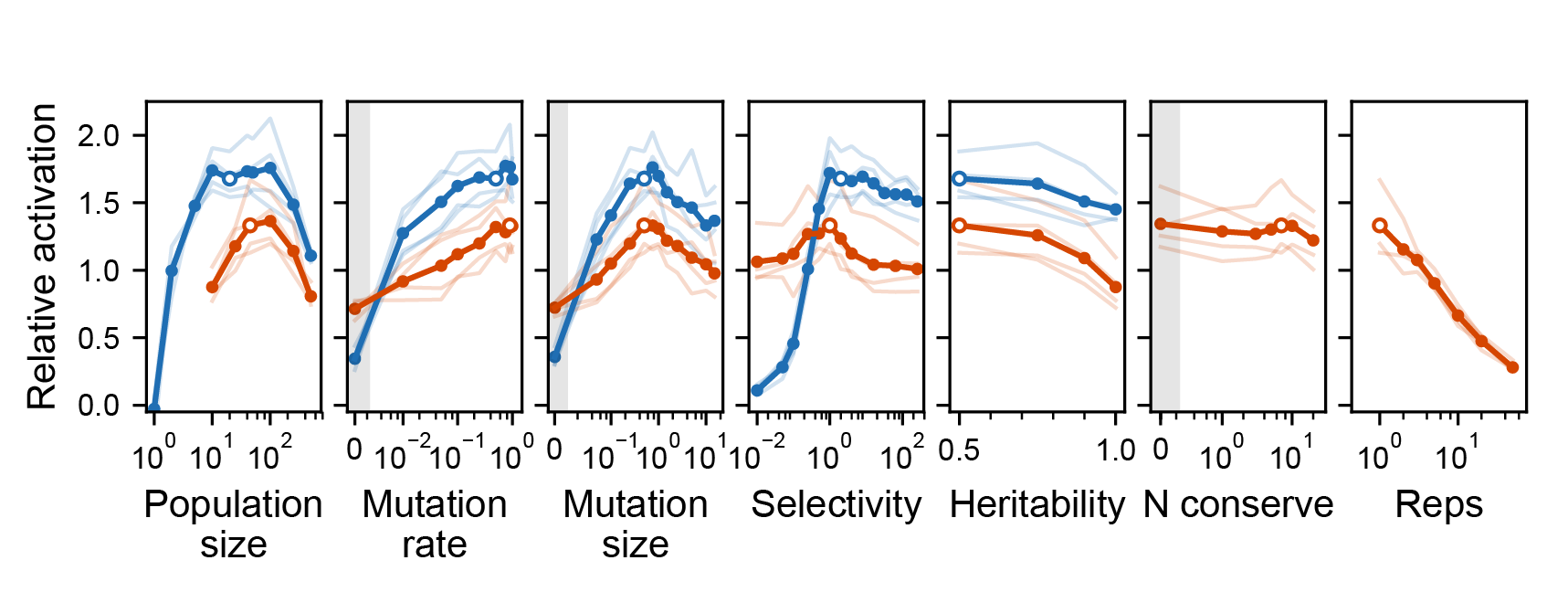

Supplement: S5 Fig — In each plot, one hyperparameter was varied while the others were held constant at default values indicated by the open circles. Dots indicate the mean of relative activation across 40 target neurons, 10 neurons each in 4 layers specified in S4 Table. Blue and orange lines indicate noiseless and noisy target units, respectively. Light colored lines indicate the mean across the 10 units within each architecture and layer. Light gray shading indicates the linear portion of a symmetrical log plot, which is used in order to show zero values. (TIF) [file pcbi.1007973.s005.tif]

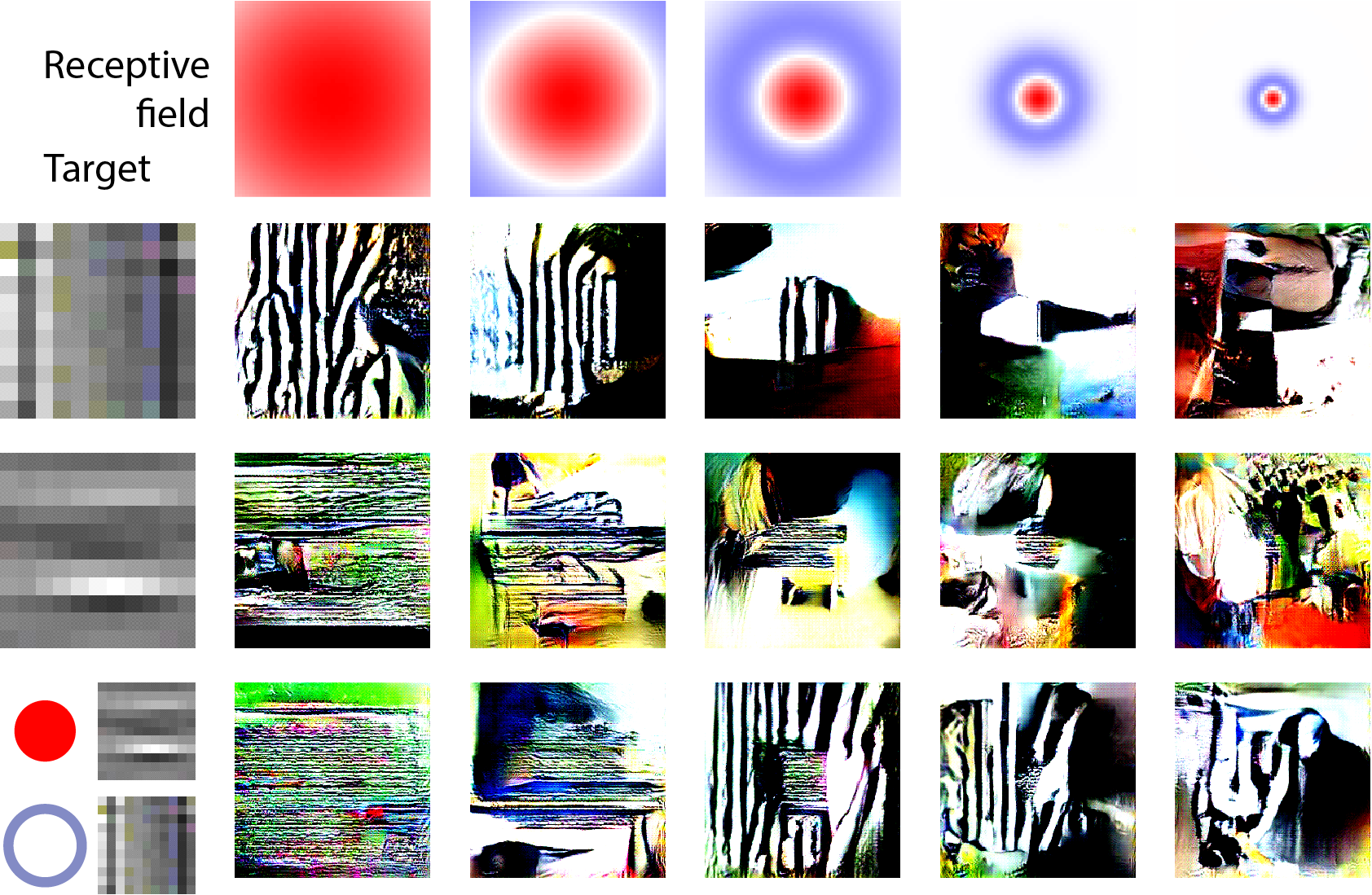

Supplement: S6 Fig — We took two feature channels (first column, rows 2 & 3) from the conv1 layer of AlexNet and tiled each spatially with positive and negative weights to create a central, circular excitatory region and a concentric suppressive ring, analogous to an excitatory classical receptive field (RF) and a suppressive extraclassical RF (first row). By maximizing responses of the constructed units, XDream created stimuli that are spatially confined and agreed with the varying RF sizes (rows 2 & 3). We also created a unit that preferred a horizontal pattern in the center and a vertical pattern in the surround; XDream was able to uncover this preference pattern as well (row 4). (TIF) [file pcbi.1007973.s006.tif]
