# Supplementary material for: XDream: Finding preferred stimuli for visual neurons using generative networks and gradient-free optimization
Source: PLoS Comput Biol. 2020 Jun 15;16(6):e1007973. doi: 10.1371/journal.pcbi.1007973 (PMC7316361; doi:10.1371/journal.pcbi.1007973)
Supplement: S1 Table — For each network, 4 layers from what is roughly the early, middle, late stages of processing, together with the output layer before softmax, were selected as targets. PlacesCNN has the same architecture as CaffeNet but is trained on the Places-205 dataset [31]. CaffeNet is as implemented in https://github.com/BVLC/caffe/tree/master/models/bvlc_reference_caffenet, PlacesCNN as in [31], and the remaining as in https://github.com/GeekLiB/caffe-model. (PDF) [file pcbi.1007973.s007.pdf]

Network	Layers			
	early	middle	late	output
CaffeNet	conv2	conv4	fc6	fc8
ResNet-152-v2	res15_eletwise	res25_eletwise	res35_eletwise	classifier
ResNet-269-v2	res25_eletwise	res45_eletwise	res60_eletwise	classifier
Inception-v3	pool2_3x3_s2	reduction_a_concat	reduction_b_concat	classifier
Inception-v4	inception_stem3	reduction_a_concat	reduction_b_concat	classifier
Inception-ResNet-v2	stem_concat	reduction_a_concat	reduction_b_concat	classifier
PlacesCNN	conv2	conv4	fc6	fc8

S1 Table.
