# Supplementary material for: XDream: Finding preferred stimuli for visual neurons using generative networks and gradient-free optimization
Source: PLoS Comput Biol. 2020 Jun 15;16(6):e1007973. doi: 10.1371/journal.pcbi.1007973 (PMC7316361; doi:10.1371/journal.pcbi.1007973)
Supplement: S2 Table — Hyperparameters used in the experiments in this paper, obtained as described in Methods separately for each generative network and for noiseless and noisy targets. (PDF) [file pcbi.1007973.s008.pdf]

Hyperparam.	Generator									
	pixel	norm1	norm2	conv3	conv4	pool5	fc6	fc7	fc8	fc6
Pop. size	30	15	10	12	10	10	20	45	20	45
Mut. rate	0.006	1	0.5	0.65	0.9	0.6	0.5	0.6	0.2	0.9
Mut. size	0.16	1.5	0.7	0.75	0.75	1	0.5	0.3	0.6	0.5
Selectivity	2.5	2	4	2.25	2.5	2.5	2	1.25	2	1
Heritability	0.6	0.5	0.5	0.5	0.5	0.55	0.5	0.5	0.5	0.5
N conserve	0	0	0	0	0	0	0	0	0	7
Reps.	1	1	1	1	1	1	1	1	1	1
Noise in target	Noiseless									Noisy

S2 Table.
