# Supplementary material for: XDream: Finding preferred stimuli for visual neurons using generative networks and gradient-free optimization
Source: PLoS Comput Biol. 2020 Jun 15;16(6):e1007973. doi: 10.1371/journal.pcbi.1007973 (PMC7316361; doi:10.1371/journal.pcbi.1007973)
Supplement: S3 Table — Hyperparameters used in the experiments in this paper, obtained as described in Methods separately for the noiseless and noisy case. The generative network was always deepsim-fc6. (PDF) [file pcbi.1007973.s009.pdf]

Hyperparam.	Algorithm			
	FDGD		NES	
N samples	20	40	20	18
Search radius	1.25	1.15	1.25	2.25
Learning rate	1.25	1.5	1.75	1.2
Search radius learning rate	N/A	N/A	0.05	0.02
Reps	1	3	1	1
Noise in target	Noiseless	Noisy	Noiseless	Noisy

S3 Table.
