# Supplementary material for: XDream: Finding preferred stimuli for visual neurons using generative networks and gradient-free optimization
Source: PLoS Comput Biol. 2020 Jun 15;16(6):e1007973. doi: 10.1371/journal.pcbi.1007973 (PMC7316361; doi:10.1371/journal.pcbi.1007973)
Supplement: S4 Table — From each layer, 10 units were randomly selected and used in hyperparameter evaluation. (PDF) [file pcbi.1007973.s010.pdf]

Network	Layer
CaffeNet	pool5
ResNet-101-v2	res32_eletwise
PlacesCNN	pool5
DenseNet-169	concat_5_31

S4 Table.
