# Supplementary material for: XDream: Finding preferred stimuli for visual neurons using generative networks and gradient-free optimization
Source: PLoS Comput Biol. 2020 Jun 15;16(6):e1007973. doi: 10.1371/journal.pcbi.1007973 (PMC7316361; doi:10.1371/journal.pcbi.1007973)
Supplement: S1 Text — (PDF) [file pcbi.1007973.s011.pdf]

Supplementary material for XDream: finding preferred stimuli for visual neurons using generative networks and gradient-free optimization

Author summary

A combinatorial explosion in the number of possible images hinders the study of neuronal tuning in visual cortex. We examine a novel method that allows neurons to dictate their preferred stimuli using closed-loop optimization and a diverse stimulus space with minimal prior assumptions. Using computational models of visual neurons to enable extensive, systematic tests, we find that the algorithm can identify stronger stimuli than possible with traditional approaches, can uncover ground truth stimulus preferences, and can robustly extrapolate to different processing stages, network architectures, and training regimes. Thus, the algorithm examined is a robust, new approach for studying neuronal tuning in a systematic and unbiased manner.

Materials and methods

Experimental design and statistical analyses

We tested XDream on units in state-of-the-art convolutional neural networks (ConvNets) pre-trained on image classification tasks. These networks are currently the best class of models of visual neuron responses [1–3]. When substituting *in silico* units for visual neurons, we considered the *activation* of a unit to a given image as analogous to the firing rate of a real neuron to a picture; this activation provides the objective function used by the optimization algorithm to search for preferred images. Further, we considered a layer in the models as analogous to a cortical area. We treated model layers as the units of comparison and report statistics across 100 randomly selected units each layer. When comparing two conditions for the same 100 units, we used a Wilcoxon signed-rank test. When comparing multiple conditions (in Fig 3d and S4 Figa), we used one-way ANOVA. We considered one-way ANOVA sufficient because the distributions were approximately normal, one-way ANOVA is conservative when there is deviation from normality and quickly converges to normal-case power while the sample sizes were reasonably large. For brevity, we only cite the p-values in text, but it should be clear from context. For multiple comparisons, p-values were corrected for false discovery rate (FDR) using the Benjamini-Hochberg procedure with an alpha level of 0.01; the number of conditions corrected will be mentioned in text with the first P-value in the group.

Target models and layers

We selected several state-of-the-art ConvNets as target models, many of which have been shown to be reasonably good models of primate visual neuron responses [2]. In each model, we tested what are approximately the early, middle, and late processing stages as well as the output layer; these layers roughly correspond to early-to-late processing stages in the ventral visual cortex [3–5]. S1 Table specifies which architectures and layers were used. One hundred (100) units were randomly selected from each layer. For convolutional layers, only the center spatial position was selected for each feature channel. All the networks were trained on the ImageNet dataset [6] except PlacesCNN, which was trained on the Places-205 dataset [7].

Optimization algorithms

The genetic algorithm works as follows: Each generation consists of n codes, where n is the population size parameter. Their corresponding fitness values $y_i, i = 1, \ldots, n$ are transformed into probability weights $w_i = \exp((y_i - \min_i(y_i))/k)$, where $k = \mathrm{stdev}_i(y_i)/s$ is analogous to temperature in the Boltzmann equation and s is the selectivity parameter (higher s is analogous to lower temperature and means high fitness is more heavily favored). To create each code in the next generation (a progeny), two codes (parents) are drawn with the probability for each code being drawn equal to $p_i = w_i / \sum_i w_i$. In our setting, the two parents do not have to be distinct. A random fraction h of vector components in the progeny is drawn from one parent and $(1 - h)$ from the other, where h is the heritability parameter. Finally, a fraction r of the components in each progeny is subject to mutation drawn from a zero-centered Gaussian of scale σ; r is the mutation rate parameter and σ the mutation size parameter.

The finite-difference gradient descent (FDGD) algorithm works as follows: A set of $2n$ sample codes $\boldsymbol{c}_{i,\pm}, i = 1, \ldots, n$ is proposed around the current center code $\boldsymbol{c}_0$ by adding to it zero-centered Gaussian perturbations $\boldsymbol{\delta}_i$ of scale σ, where σ is the search radius parameter. The samples are *antithetic*, meaning that $\boldsymbol{c}_{i,\pm} = (\boldsymbol{c}_0 \pm \boldsymbol{\delta}_i)$. The gradient estimate is then $\Delta \boldsymbol{c}_0 = \sum_i \Delta y_i \boldsymbol{\delta}_i / \|\boldsymbol{\delta}_i\|^2$, where $\Delta y_i = (y_{i,+} - y_{i,-})$ and $\|\cdot\|^2$ is the L2 norm. The new center is then $\boldsymbol{c}_0' = (\boldsymbol{c}_0 + \eta \Delta \boldsymbol{c}_0)$, where η is the learning rate parameter.

The natural evolution strategies (NES) algorithm aims to maximize not $y = f(\boldsymbol{c}_0)$ at a center code $\boldsymbol{c}_0$, but the expectation $\mathbb{E}_{\pi(\boldsymbol{c})}[f(\boldsymbol{c})]$ over a search distribution $\pi(\boldsymbol{c})$. We refer the reader to [8] for motivation and derivation. The implementation is as follows: The search distribution is a Gaussian of scale σ around the current center $\boldsymbol{c}_0$. A set of $2n$ antithetic samples, $\boldsymbol{c}_{i,\pm}, i = 1, \ldots, n$, is proposed as above. The gradient estimate for the center is $\Delta \boldsymbol{c}_0 = \frac{1}{n\sigma^2} \sum_{i,\pm} s_{i,\pm} \boldsymbol{\delta}_i$. The new center is $\boldsymbol{c}_0' = (\boldsymbol{c}_0 + \eta \Delta \boldsymbol{c}_0)$, where η is the learning rate parameter. The scale parameter is also updated using the gradient $\Delta \sigma = \frac{1}{n\sigma} \sum_{i,\pm} s_{i,\pm} \left(\frac{\boldsymbol{\delta}_i^2}{\sigma^2} - 1 \right)$ and a separate learning rate. Note that we use the same σ for all components in the image code and thus do not model a multidimensional Gaussian nor any covariances, different from the general case discussed in [8]. However, we do update the scale of the search distribution, different from [9]. We have tried updating separate, independent σ's for each component in the image code, but the performance is much worse, presumably because there is too

little information to reliably estimate gradients for the second moment.

Converting images to image codes

In several cases (e.g., Fig 3 and S3 Fig), we needed to convert an image into an image code in the input space of the image generator. It is a generally hard problem to invert a pre-trained generative model with high-fidelity (see for example [10, 11] for more involved methods). We used two heuristic methods for this purpose, which we refer to as “opt” and “ivt.” In the “opt” method, an image code was zero-initialized and iteratively optimized using backpropagation and gradient descent to minimize the pixel-wise difference between the generated image and the target image. In the “ivt” method, the fc6-layer encoding of the target image by CaffeNet was used as the image code, because the generator was originally trained to invert this encoding [12].

Hyperparameters

To choose a set of good hyperparameters, we used a greedy algorithm that maximized performance over a small set of target units by varying one hyperparameter at a time. To keep the computation tractable, we used 12 units total, 3 randomly chosen from the output layer of each of 4 networks: CaffeNet, ResNet-152, Inception-v2, and PlacesCNN. Starting from an educated guess of hyperparameter values, one hyperparameter was chosen at a time. Four test values were chosen around the current value with a pre-defined step size, and optimization performance was measured with the test values. The value that yielded the best performance was set as the current value. Then, another hyperparameter was chosen to be varied. The same hyperparameter was not chosen again until all others had been considered once; we call each repeat of all hyperparameters one *round*. If no hyperparameter was updated in a given round, the step size was decreased for the hyperparameter that had not been updated for the longest time. This procedure was repeated until all pre-defined, progressively decreasing step sizes for each parameter were exhausted. The final best parameter settings were used as the default values. Hyperparameters were optimized separately for each optimization algorithm, for each image generator, and for the noiseless and noisy case (described below). The hyperparameters used in this paper are listed in S2 Table and S3 Table. An example of hyperparameter landscape around the current best values can be seen in S5 Fig.

Stochastic neuron models

Biological neurons can yield different responses upon repeated presentation of the same stimulus. To examine the effect of this variability on XDream, we artificially added noise to the unit activations. Let y be the activation value of a ConvNet unit. The activation value corrupted by stochastic noise was drawn from $Y \sim \text{Poisson}(\max(0, \gamma))$, where the rate parameter γ is analogous to the number of spikes of a neuron and equals y times a constant scaling factor. The scaling factor is necessary because the signal-to-noise ratio (SNR) of a Poisson process—in terms of squared mean over standard deviation—increases as the rate of the Poisson process increases, but normalized vs. unnormalized networks and different layers in

an unnormalized network produce activation values of different scales. We used a scaling factor of $20/\hat{y}$, where $\hat{y}$ is the median of the max activation to 2,500 random ImageNet images, and 20 is a realistic number of spikes a biological neuron may fire to a preferred stimulus within a measurement time of 200 ms. Note that a Poisson process with $\gamma = 20$ has an SNR of $\mu^2/\sigma^2 = 20$; the SNR will be lower for less optimal images with a lower rate parameter. To simulate repeated image presentations, we simply drew multiple Y values from the same Poisson distribution, with the realistic trade-off that fewer unique images could be presented given the same total number of allowed queries.

Source of the target images in S3 Fig

The leftmost 2 images were manually created; the third image was synthesized as described in [13]; the fourth image is from the ImageNet test set; the rightmost 3 images are public domain images from NASA and The Metropolitan Museum of Art.

Additional Experiments

XDream can efficiently search a large and diverse stimulus space to find the ground truth optimal stimulus

Is XDream limited in the kind of images it can find? This is an essential question for the utility of XDream. An analysis of this question was presented in the supplement to [14], but the question is relevant here so we discuss the analyses in the current context using slightly different data. Because XDream optimizes in the latent space of a generative network, a first constraint is the range of images that can be created by the generative network. It is hard to quantify what fraction of all possible images is represented by a generative network. Instead, we qualitatively assessed the expressiveness of the generative network by challenging it to synthesize diverse, arbitrarily selected target images (S3 Fig, row 1). To find synthetic images that approximate the target images, we used two heuristic methods: 1) iteratively optimizing an image code to minimize the pixel-wise difference between the generated and target image (labeled "opt"); 2) directly using CaffeNet fc6 representation of the image as the image code, because the generative network was originally trained to invert this representation (labeled "ivt"; see Methods for details of both methods). S3 Fig, rows 2–3 show that the generative network is able to represent, at least approximately, all the tested target images.

Not only does XDream need to represent diverse images, it must also efficiently find those images (i.e., in a reasonable number of queries). When investigating the activation of units in a target model as in Fig 1, we do not know what the ground truth global optimum image is. To evaluate whether XDream is in principle capable of finding the global maximum, we considered a simple toy model where we know the ground truth best image by construction. For a target image, we minimized the mean squared difference between that image and any input image, computed using either the pixel-level representation of the image (S3 Fig, row 4), or, separately, the CaffeNet pool5 layer representation of the image (S3 Fig, row 5). With

both representations, XDream was able to uncover an image resembling the original using only 10,000 image presentations. The optimized images generated by XDream were not identical to the ground truth target. At least part of the remaining differences could be attributed to the loss function: Pixel-wise loss is known to lead to excessive smoothing, and pool5 loss is expected to lose some detailed features and spatial information due to pooling operations and ReLU activations in preceding layers.

These results show that XDream is highly "expressive"—i.e., it can generate a very large and diverse set of images—and suggest that it is in principle possible to reach close to the global maximum. Of note, these results do *not* show that *any* image can be generated, nor do they provide a proof of convergence to the global maximum for all visual neurons.

Image generators that use high-level representations work equally well

An essential component of XDream is the image generator. In other experiments in the paper, we used a generative network based on CaffeNet fc6 representations [12]. Here, we examine whether the choice of image generator matters for the performance of XDream, and whether the answer depends on the target unit. We hypothesized that image generators based on pixels or low-level representations would not work well, and high-level features may be required for efficient optimization. To test this hypothesis, we evaluated the family of DeePSiM generators, which are generative neural networks trained to invert each layer of CaffeNet respectively [12]. The 8 image generators are all of similar depth (11–13 layers) and architecture (feedforward with no bypass or recurrent connections), although image generators trained on higher layers have more parameters due to having more convolutional filters and, in the case of fc-layer generators, having fully-connected layers. In addition to generative neural networks, we also tested a control image "generator" where the image code was directly parameterized by the flattened pixel array. Therefore, searching the representational space of this generator is equivalent to brute-force search of possible images. As target units, we tested early, late, and output layers of CaffeNet, as well as the output layer of Inception-ResNet-v2 to evaluate cross-architecture generalization.

Using different image generators had a statistically significant effect on optimized activation in all layers (S4 Fig panel a; $p < 10^{-24}$, FDR corrected for 4 tests in 4 layers). Higher layer-based image generators appeared to work better than lower layer-based ones for all target model layers except CaffeNet conv2. On the other hand, the difference appeared small among high-level image generators. The generators deepsim-pool5, deepsim-fc6, and deepsim-fc7 performed similarly in CaffeNet conv2 and fc8 layers ($p = 0.3$, FDR corrected for 4 tests), whereas deepsim-pool5 and deepsim-fc6 were equivalent for CaffeNet fc6, fc8, and Inception-ResNet-v2 classifier layers ($p = 0.09, 0.12, 0.71$ respectively, FDR corrected for 32 tests comparing each generator to deepsim-fc6 in each target layer). These results indicate that the image generator does not need to be tailored to the target model unit, consistent with the ability of XDream to generalize across architectures demonstrated in Fig 2. Of note, in Fig 2a, the highest relative activation was always obtained for the "late layer" of different networks; the results here show that this was not due to the use of the fc6-based generative network, since other high-level generators worked

similarly well. The pixel-based image generator worked more poorly than generative neural networks ($p < 10^{-4}$; FDR-corrected for 32 tests comparing each generator to the raw pixel generator in each target layer), with the following exceptions: in target layer CaffeNet conv2 except when compared to generator deepsim-norm2 ($p = 1$ compared to deepsim-norm2; $p > 0.14$ compared to other generators in CaffeNet conv2; $p < 10^{-4}$ in all other comparisons; FDR-corrected for 32 tests comparing each generator to raw-pixel in each target layer). These results are consistent with the hypothesis that it is beneficial to use an image generator based on higher-level image features.

We also directly tested the ability of XDream to generalize beyond its component generator's training set: We compared two generators with the same architecture (DeePSiM-fc6) trained on different datasets (ImageNet and Places365) in terms of how well the generators could be used to optimize units in two classifiers with the same architecture (CaffeNet and PlacesCNN) trained separately on the same two datasets. Both generators performed similarly well on both classifiers (S4 Fig panel b), supporting the notion that the generators have learned general features, at least across these two datasets.

Additional Discussion

XDream extends the idea of feature visualization to situations where knowledge of the underlying architecture is not available by using gradient-free optimization algorithms. Another approach that has inspired the current work is to use a genetic algorithm to search a parametrically-defined stimulus space [15–17]. XDream extends this idea by using a more diverse stimulus space learned by a generative neural network, which does not require prior knowledge or intuitions about the tuning properties of the neurons under study. In addition, we frame the approach more broadly, incorporating additional image generators and optimization algorithms.

As introduced in the main text, another general approach to black box feature visualization is through substitute models that mimic the response properties of the black box, but are themselves fully accessible. In contrast to the substitute model approach, XDream directly operates on the black box of interest. Studies have found that the direct approach is both free of transferability problems (because no substitute model is involved) and more sample efficient [9, 18]. In this light, when comparing the substitute-model approach and the direct approach for studying neural coding properties, test case performance and sample efficiency is an important consideration, as is test case performance. Recent preliminary results suggest that some ConvNet-based models do not fully extrapolate to images very different from those used during training [4, 14]. If so, these models may not be adequately guiding the exploration of image space, of which training images can only represent a small fraction.

The performance of XDream is robust to many design and parameter choices in the algorithm. The use of the genetic algorithm and several related high-level generative networks [12] are adequate and perhaps ideally suited for this family of tasks. In addition, empirically optimized hyperparameter values are listed in S2 Table. The robustness of the algorithm to parameter variations (S5 Fig) indicates that it is unlikely that

specific parameters will drastically change performance and suggests that there is no need to tailor parameters to specific neurons, areas, or species. Nevertheless, it may be possible to further fine-tune the parameters to the neurons under study. The choice of the generative model is particularly relevant. Interpreted as a prior on the stimuli to test, the choice of a generative model may constitute a bias, analogous to how an investigator's selection of stimuli could result in bias [19, 20]. However, unlike manual stimulus selection, the generative model is a much less restrictive prior. Moreover, since animals live in a natural world, a bias on natural images (induced by training on ImageNet) seems a reasonable, ethologically-relevant prior [21]. Finally, results in Fig 2a and S4 Fig panel b show that using a generator outside its training domain does not significantly impact the optimization performance of XDream.

On repeated runs from different initializations, XDream finds related but not identical images that trigger similar activation values (Fig 3b). We speculate that there may be a whole "invariance manifold" of related images that elicit similar responses in any given unit. Indeed, certain neurons in high-level visual cortical areas are approximately invariant to image transformations such as position and scale changes. The invariance manifold may take several possible forms: It may be a linear subspace (within the image code space); it may comprise a convex set (i.e., points on an interpolation between any two members of the set are also members of the set); it may comprise a connected set (i.e., one can travel between any two points in the set while always remaining in the set); or, it may have none of these properties. In a sense, understanding the tuning of a neuron is equivalent to identifying the image representation space in which the neuron has a simple (e.g., linear) invariance manifold. The fact that we do not find linear or convex manifolds, particularly for later layer units (Fig 3b), suggests that the generative networks we used still do not perfectly match the feature representation of the units.

The fact that XDream extrapolates across layers, architectures, and models trained on different datasets bodes well for it to extrapolate to different ventral stream areas and even visual cortices of different species, but However, these models cannot replace actual neurons. For example, features like feedback and lateral inputs, which underlie "extra-classical" and other contextual effects, are not captured in the ConvNet models tested. As proof-of-concept, we constructed a toy model of extraclassical stimulus preference, and found that XDream could uncover those properties (S6 Fig). Ultimately, experiments with biological neurons are necessary to evaluate how well XDream will actually generalize to different neuronal types, brain areas, and species.

References

1. Yamins DLK, Hong H, Cadieu CF, Solomon EA, Seibert D, DiCarlo JJ. Performance-optimized hierarchical models predict neural responses in higher visual cortex. Proceedings of the National Academy of Sciences. 2014;111(23):8619–8624. doi:10.1073/pnas.1403112111.

2. Schrimpf M, Kubilius J, Hong H, Majaj NJ, Rajalingham R, Issa EB, et al. Brain-Score: Which Artificial Neural Network for Object Recognition is most Brain-Like? bioRxiv. 2018;.

3. Cadena SA, Denfield GH, Walker EY, Gatys LA, Tolias AS, Bethge M, et al. Deep convolutional models improve predictions of macaque V1 responses to natural images. PLOS Computational Biology. 2019;15(4):1–27. doi:10.1371/journal.pcbi.1006897.

4. Bashivan P, Kar K, DiCarlo JJ. Neural population control via deep image synthesis. Science. 2019;364(6439). doi:10.1126/science.aav9436.

5. Abbasi-Asl R, Chen Y, Bloniarz A, Oliver M, Willmore BDB, Gallant JL, et al. The DeepTune framework for modeling and characterizing neurons in visual cortex area V4. bioRxiv. 2018;.

6. Russakovsky O, Deng J, Su H, Krause J, Satheesh S, Ma S, et al. ImageNet Large Scale Visual Recognition Challenge. International Journal of Computer Vision. 2015;115(3):211–252. doi:10.1007/s11263-015-0816-y.

7. Zhou B, Lapedriza A, Xiao J, Torralba A, Oliva A. Learning Deep Features for Scene Recognition using Places Database. In: Advances in Neural Information Processing Systems 29; 2014. p. 487–495.

8. Wierstra D, Schaul T, Glasmachers T, Sun Y, Peters J, Schmidhuber J. Natural Evolution Strategies. Journal of Machine Learning Research. 2014;15:949–980.

9. Ilyas A, Engstrom L, Athalye A, Lin J. Black-box Adversarial Attacks with Limited Queries and Information. In: Dy J, Krause A, editors. Proceedings of the 35th International Conference on Machine Learning. vol. 80 of Proceedings of Machine Learning Research; 2018. p. 2137–2146.

10. Gu J, Shen Y, Zhou B. Image Processing Using Multi-Code GAN Prior. arXiv. 2019;.

11. Bau D, Strobelt H, Peebles W, Wulff J, Zhou B, Zhu JY, et al. Semantic Photo Manipulation with a Generative Image Prior. ACM Trans Graph. 2019;38(4). doi:10.1145/3306346.3323023.

12. Dosovitskiy A, Brox T. Generating Images with Perceptual Similarity Metrics based on Deep Networks. In: Advances in Neural Information Processing Systems; 2016. p. 658–666.

13. Portilla J, Simoncelli EP. A Parametric Texture Model Based on Joint Statistics of Complex Wavelet Coefficients. International Journal of Computer Vision. 2000;40(1):49–70. doi:10.1023/A:1026553619983.

14. Ponce CR, Xiao W, Schade P, Hartmann TS, Kreiman G, Livingstone MS. Evolving Images for Visual Neurons Using a Deep Generative Network Reveals Coding Principles and Neuronal Preferences. Cell. 2019;177:999–1009. doi:10.1016/j.cell.2019.04.005.

15. Yamane Y, Carlson ET, Bowman KC, Wang Z, Connor CE. A neural code for three-dimensional object shape in macaque inferotemporal cortex. Nature Neuroscience. 2008;11(11):1352–1360. doi:10.1038/nn.2202.

16. Carlson ET, Rasquinha RJ, Zhang K, Connor CE. A Sparse Object Coding Scheme in Area V4. Current Biology. 2011;21(4):288–293. doi:10.1016/j.cub.2011.01.013.

17. Vaziri S, Carlson ET, Wang Z, Connor CE. A Channel for 3D Environmental Shape in Anterior Inferotemporal Cortex. Neuron. 2014;84(1):55–62. doi:10.1016/j.neuron.2014.08.043.

18. Chen PY, Zhang H, Sharma Y, Yi J, Hsieh CJ. ZOO: Zeroth Order Optimization Based Black-box Attacks to Deep Neural Networks without Training Substitute Models. In: Proceedings of the 10th ACM Workshop on Artificial Intelligence and Security; 2017. p. 15–26.

19. Olshausen B, Field D. What Is the Other 85 Percent of V1 Doing? In: Van Hemmen J, Sejnowski T, editors. 23 Problems in Systems Neuroscience. Oxford: Oxford University Press; 2005. p. 182–211.

20. Carandini M, Demb JB, Mante V, Tolhurst DJ, Dan Y, Olshausen BA, et al. Do We Know What the Early Visual System Does? Journal of Neuroscience. 2005;25(46):10577–10597. doi:10.1523/jneurosci.3726-05.2005.

21. Simoncelli EP, Olshausen BA. Natural Image Statistics and Neural Representation. Annual Review of Neuroscience. 2001;24(1):1193–1216. doi:10.1146/annurev.neuro.24.1.1193.
