# Supplementary material for: XDream: Finding preferred stimuli for visual neurons using generative networks and gradient-free optimization
Source: PLoS Comput Biol. 2020 Jun 15;16(6):e1007973. doi: 10.1371/journal.pcbi.1007973 (PMC7316361; doi:10.1371/journal.pcbi.1007973)
Supplement: Attachment — Submitted filename: PCOMPBIOL-D-19-01642_reviewer.pdf [file pcbi.1007973.s012.pdf]

# PLOS Computational Biology

## XDream: finding preferred stimuli for visual neurons using generative networks and gradient-free optimization

--Manuscript Draft--

| | |
|---|---|
| **Manuscript Number:** | PCOMPBIOL-D-19-01642 |
| **Full Title:** | XDream: finding preferred stimuli for visual neurons using generative networks and gradient-free optimization |
| **Short Title:** | Finding preferred visual stimuli via optimization |
| **Article Type:** | Research Article |
| **Keywords:** | Computational neuroscience; visual processing; neuronal coding; ventral visual stream; preferred stimuli; deep generative neural networks; genetic algorithm; convolutional neural networks |
| **Abstract:** | A longstanding question in sensory neuroscience is what types of stimuli drive neurons to fire. The characterization of effective stimuli has traditionally been based on a combination of intuition, insights from previous studies, and luck. A new method termed XDream (EXtending DeepDream with real-time evolution for activation maximization) combined a generative neural network and a genetic algorithm in a closed loop to create strong stimuli for neurons in the macaque visual cortex. Here we evaluate how the method compares to brute-force search, how well the method generalizes to different neurons and processing stages, whether the design choices are optimal, and whether those choices need to be adapted to the neurons under study. We use ConvNet units as in silico models of neurons, enabling extensive and systematic experiments that would be prohibitive with biological neurons. We show that XDream can efficiently find preferred features for visual units without any prior knowledge about them. XDream extrapolates to different layers, architectures, and developmental regimes, performing better than brute-force search and often even better than exhaustive sampling of > 1 million images. Furthermore, XDream is robust to multiple image generators, optimization algorithms, and hyperparameters, suggesting that its performance is locally near-optimal. Lastly, we found no significant advantage to problem-specific tuning. These results establish expectations and provide practical recommendations for using XDream to investigate neural coding in biological preparations. Overall, XDream is an efficient, general, and robust algorithm for uncovering neuronal tuning preferences using a vast and diverse stimulus space. |
| **Additional Information:** | |
| **Question** | **Response** |
| **Financial Disclosure**<br><br>Enter a financial disclosure statement that describes the sources of funding for the work included in this submission. Review the submission guidelines for detailed requirements. View published research articles from *PLOS Computational Biology* for specific examples.<br><br>This statement is required for submission and **will appear in the published article** if the submission is accepted. Please make sure it is accurate. | W.X. and G.K. are supported by the Center for Brains, Minds and Machines funded by NSF528STC award CCF-1231216 and also by NIH R01EY026025. The funders had no role in study design, data collection and analysis, decision to publish, or preparation of the manuscript.<br>https://www.nsf.gov<br>https://www.nih.gov |

Powered by Editorial Manager® and ProduXion Manager® from Aries Systems Corporation

| | |
|---|---|
| **Unfunded studies**<br>Enter: *The author(s) received no specific funding for this work.*<br><br>**Funded studies**<br>Enter a statement with the following details:<br>• Initials of the authors who received each award<br>• Grant numbers awarded to each author<br>• The full name of each funder<br>• URL of each funder website<br>• Did the sponsors or funders play any role in the study design, data collection and analysis, decision to publish, or preparation of the manuscript?<br>• **NO** - Include this sentence at the end of your statement: *The funders had no role in study design, data collection and analysis, decision to publish, or preparation of the manuscript.*<br>• **YES** - Specify the role(s) played.<br><br>* typeset | |
| **Competing Interests**<br><br>Use the instructions below to enter a competing interest statement for this submission. On behalf of all authors, disclose any competing interests that could be perceived to bias this work—acknowledging all financial support and any other relevant financial or non-financial competing interests.<br><br>This statement **will appear in the published article** if the submission is accepted. Please make sure it is accurate. View published research articles from *PLOS Computational Biology* for specific examples. | The authors have declared that no competing interests exist. |

Powered by Editorial Manager® and ProduXion Manager® from Aries Systems Corporation

| | |
|---|---|
| **NO authors have competing interests**<br><br>Enter: *The authors have declared that no competing interests exist.*<br><br>**Authors with competing interests**<br><br>Enter competing interest details beginning with this statement:<br><br>*I have read the journal's policy and the authors of this manuscript have the following competing interests: [insert competing interests here]*<br><br>* typeset | |
| **Data Availability**<br><br>Authors are required to make all data underlying the findings described fully available, without restriction, and from the time of publication. PLOS allows rare exceptions to address legal and ethical concerns. See the PLOS Data Policy and FAQ for detailed information.<br><br>A Data Availability Statement describing where the data can be found is required at submission. Your answers to this question constitute the Data Availability Statement and **will be published in the article**, if accepted.<br><br>**Important:** Stating 'data available on request from the author' is not sufficient. If your data are only available upon request, select 'No' for the first question and explain your exceptional situation in the text box.<br><br>Do the authors confirm that all data underlying the findings described in their manuscript are fully available without restriction? | Yes - all data are fully available without restriction |
| **Describe where the data may be found in full sentences. If you are copying our sample text, replace any instances of XXX with the appropriate details.** | All data underlying the findings described in their manuscript will be made available in the same repository where the software is deposited (URL included in text) before publication of the paper. |

| | |
|---|---|
| • If the data are **held or will be held in a public repository**, include URLs, accession numbers or DOIs. If this information will only be available after acceptance, indicate this by ticking the box below. For example: *All XXX files are available from the XXX database (accession number(s) XXX, XXX.*).<br>• If the data are all contained **within the manuscript and/or Supporting Information files**, enter the following: *All relevant data are within the manuscript and its Supporting Information files.*<br>• If neither of these applies but you are able to provide **details of access elsewhere**, with or without limitations, please do so. For example:<br><br>*Data cannot be shared publicly because of [XXX]. Data are available from the XXX Institutional Data Access / Ethics Committee (contact via XXX) for researchers who meet the criteria for access to confidential data.*<br><br>*The data underlying the results presented in the study are available from (include the name of the third party and contact information or URL).*<br>• This text is appropriate if the data are owned by a third party and authors do not have permission to share the data.<br><br>* typeset | |
| Additional data availability information: | |

September 24, 2019

To Whom It May Concern:

We are enclosing a manuscript entitled “Finding Preferred Stimuli for Visual Neurons Using Generative Networks and Gradient-Free Optimization” for your consideration for publication as a methods or software article in PLOS Computational Biology.

A central question in Neuroscience is the elucidation of tuning properties of cortical neurons. In the visual system, neurophysiological recordings over the last five decades have discovered neurons with center-surround receptive fields, tuning to bars of specific orientations, tuning to curvature, all the way to tuning to complex objects such as faces. Despite these textbook notions of stimulus selectivity along ventral visual cortex, we still do not know whether these stimuli are truly optimal or whether there could be other images that trigger even more selective responses. The main challenge to defining preferred stimuli is that it is impossible to exhaustively sample all images. Instead, past investigations depended on ingenious heuristics that were based on intuitions, natural image statistics, computational models, and random exploration.

We recently introduced a new method to address this challenge and systematically investigate neural coding properties in an unbiased manner. We refer to this method as XDream (for EXtending DeepDream with Real-time Evolution for Activation Maximization). In Ponce et al., Cell 2019, we presented initial experimental evidence of the method’s feasibility in macaque monkey neurons. This method elicited strong interest, leading to a large number of important questions about the performance characteristics and practical application of the algorithm. In the current manuscript, we quantitatively investigate these questions, with the goal of making the algorithm more widely applicable by the research community. By using computational models as a proxy for neuronal recordings, we are able to systematically and extensively test critical questions about the utility of the algorithm. Specifically, we determined the following performance characteristics of the algorithm:

- XDream can find “super stimuli” that trigger stronger responses than more than one million natural images;
- XDream is highly “expressive,” allowing extensive sampling of image space;
- XDream can find globally optimal stimuli;
- The ability of XDream to find effective stimuli is general across stages of processing from early to late vision, across different network architectures, and across different training regimes analogous to developmental shaping of selectivity;
- XDream is robust to different random initial conditions and performs well with them.

In addition, we extended the method in the following way:

- We incorporated several image generators and two other optimization algorithms, and empirically optimized parameter settings. The performance of XDream is robust to a wide range of parameter settings and algorithmic choices.

Based on the enclosed results, we can provide specific recommendations for applying this method. Given the ability of the XDream method to uncover stimulus preferences in an unbiased, efficient, and robust manner, we expect that these results will be of interest to the large community of researchers using electrophysiological recordings, calcium imaging, or other methods to functionally study visual cortex across species and visual areas. The principles of the algorithm could be informative for designing similar methods for studying other sensory modalities.

The source code of the algorithm is openly shared with the community (https://github.com/willwx/XDream). As a part of publishing the software, we are updating the repository as recommended in the pre-submission inquiry and will include license information, more API documentation, and more examples before acceptance of the paper.

The manuscript is not under consideration elsewhere. We thank you in advance for considering this submission.

Yours sincerely,

Will Xiao and Gabriel Kreiman

# XDream: finding preferred stimuli for visual neurons using generative networks and gradient-free optimization

Will Xiao[1*], Gabriel Kreiman[2,3],

**1** Department of Molecular and Cellular Biology, Harvard University, Cambridge, MA, U.S.A.
**2** Center for Brains, Minds, and Machines, Boston, MA, U.S.A.
**3** Department of Ophthalmology, Boston Children's Hospital, Boston, MA, U.S.A

* xiaow@fas.harvard.edu

**Short title** : Finding preferred visual stimuli via optimization

## Abstract

A longstanding question in sensory neuroscience is what types of stimuli drive neurons to fire. The characterization of effective stimuli has traditionally been based on a combination of intuition, insights from previous studies, and luck. A new method termed XDream (E**X**tending **D**eepDream with **r**eal-time **e**volution for **a**ctivation **m**aximization) combined a generative neural network and a genetic algorithm in a closed loop to create strong stimuli for neurons in the macaque visual cortex [1]. Here we evaluate how the method compares to brute-force search, how well the method generalizes to different neurons and processing stages, whether the design choices are optimal, and whether those choices need to be adapted to the neurons under study. We use ConvNet units as *in silico* models of neurons, enabling extensive and systematic experiments that would be prohibitive with biological neurons. We show that XDream can efficiently find preferred features for visual units without any prior knowledge about them. XDream extrapolates to different layers, architectures, and developmental regimes, performing better than brute-force search and often even better than exhaustive sampling of $> 1$ million images. Furthermore, XDream is robust to multiple image generators, optimization algorithms, and hyperparameters, suggesting that its performance is locally near-optimal. Lastly, we found no significant advantage to problem-specific tuning. These results establish expectations and provide practical recommendations for using XDream to investigate neural coding in biological preparations. Overall, XDream is an efficient, general, and robust algorithm for uncovering neuronal tuning preferences using a vast and diverse stimulus space.

## Author summary

A combinatorial explosion in the number of possible images hinders the study of neuronal tuning in visual cortex. We examine a novel method that allows neurons to dictate their preferred stimuli using closed-loop optimization and a diverse stimulus space. Using computational models of visual neurons to enable extensive, systematic tests, we find that the algorithm can identify stronger stimuli than possible with traditional approaches, can uncover ground truth stimulus preferences, and can robustly extrapolate to different processing stages, network architectures, and training regimes.

Thus, the algorithm examined is a robust new approach for studying neuronal tuning in a systematic and unbiased manner.

## Introduction

What stimuli excite a neuron, and how can we find them? Considering vision as a paradigmatic example, the selection of stimuli to probe neural activity has shaped our understanding of how visual neurons represent information. It is practically impossible to exhaustively evaluate neuronal responses to images, due to the combinatorically large number of possible images. Instead, investigators have traditionally selected stimuli guided by natural image statistics, behavioral relevance, theoretical postulates about internal representations, intuitions from previous studies, and serendipitous findings. Stimuli selected in this way underlie our current understandings of how circular center-surround receptive fields [2] give rise to orientation tuning [3], then to encoding of more complex shapes such as curvatures [4, 5], and further to selective responses to complex objects such as faces [6–8].

Despite the progress made in understanding visual cortex by testing limited sets of hand-chosen stimuli, these experiments could be missing the true feature preferences of neurons. In other words, there could be other images that drive visual neurons better than those found so far, and such images could lead us to revisit our current descriptions of feature tuning in visual cortex.

A recently introduced method shows promise to begin bridging the gap. Termed XDream (e**X**tending **D**eepDream with **r**eal-time **e**volution for **a**ctivation **m**aximization), this method combines a genetic algorithm and a deep generative neural network [9]—both inspired by previous work [10–13]—to evolve images that trigger high activation in neurons [1]. XDream can generate strong stimuli for neurons in macaque inferior temporal (IT) and primary visual cortex (V1).

The performance and design options of XDream have not been thoroughly evaluated, due to the time-intensiveness of neuronal recordings and the difficulty to fully control all experimental variables. To overcome these challenges, here we test the performance of XDream using state-of-the-art *in silico* models of visual neurons in lieu of real neurons, in the same spirit of [14]. Specifically, we use convolutional neural networks (ConvNets) pre-trained on visual recognition tasks as an approximation to the computations performed along ventral visual cortex [15–17]. Using these models as a proxy for real neurons allows us to compare synthetic stimuli with a large set of reference images, to evaluate XDream's performance across processing stages, model architectures, and training regimes, to empirically optimize algorithm and parameter choices in a systematic fashion, and to disentangle the effects of response stochasticity.

Should probably reference Zeiler and Fergus also: https://arxiv.org/abs/1311.2901

Although there is a rich literature in computer science on feature visualization [18–20], we focus on the more biologically relevant scenario where there is no information about the architecture and weights of the target model, and where we only have access to a few, potentially stochastic, activation values from the neurons. These conditions reflect those prevailing in neuronal recordings and are fundamentally different from the assumptions made in computer science studies.

can

Under these realistic constraints, we show that XDream could still reliably and efficiently uncover preferred features of units with a wide range of response properties, generalizing to different processing stages within a network, different network architectures, and different training datasets. Furthermore, XDream performed equally well with a wide range of algorithmic and parameter choices, indicating that it is a robust algorithm. Based on these results, we suggest parameters to use and results that can be expected when using XDream to investigate neuronal tuning properties. Our findings suggest that XDream is a general and robust method for investigating neuronal

preferences in visual cortex.

# Results

## Random exploration of stimulus space is inefficient

A common approach for exploring neuronal selectivity is to use arbitrarily selected images, often from a limited number of categories (for example in [8]). Thus, we considered random exploration as a baseline for comparison. We used the AlexNet architecture as the target model ( [25], implemented as CaffeNet; Table 2) and sampled images from ImageNet ( [23]; ILSVRC12 dataset, 1,431,167 images), a large dataset common in computer vision that also contains the training set of CaffeNet. We randomly sampled $n$ images either from all of ImageNet or from 10 categories randomly selected from the 1,000 training categories in ImageNet ($n/10$ images per category). For units in different layers of the network, we evaluated the activation value to these images, and calculated the maximum *relative activation* defined as the ratio between the maximum activation in the $n$ random images and the maximum activation in all of ImageNet (S1 Fig). By definition, the relative activation for the best image in ImageNet is 1, which is also an upper bound on the observed relative activation values. Randomly selected images with both approaches typically yielded relative activation values well below 1. As expected, the maximum observed relative activation increased with $n$ but only did so slowly, with near-logarithmic growth. Moreover, for later layers (e.g., fc8), sampling from only 10 categories yielded significantly worse results than sampling completely randomly, which we hypothesize is because the small number of categories imposes a bottleneck on the diversity of high-level features represented. In neuroscience studies, category selection is clearly not completely random; investigators may have intuitions and prior knowledge about the types of stimuli that are likely more effective. To the extent that those intuitions are correct, they can enhance the search process. However, those intuitions are seldom guided by systematic examination of stimulus space and could well miss important types of stimuli.

**Fig 1. Overview of the XDream method. a)**, XDream combines in a closed loop an image generator (e.g., a generative adversarial network), a target neuron (e.g., a unit in a ConvNet), and a non-gradient-based optimization algorithm (e.g., a genetic algorithm). In each iteration, the optimization algorithm proposes a set of codes, the image generator synthesizes the codes into images, the images are evaluated by the target neuron to produce one scalar score per image, and the scores are used by the optimization algorithm to propose a new set of codes. Importantly, no optimization gradient is needed from the neuron. **b,c)**, An example experiment targeting CaffeNet layer fc8, unit 1. **b)**, mean activation achieved over 500 generations, 20 images per generation (10,000 total image presentations). **c)**, Images obtained at a few example generations indicated by minor x-ticks in **b)**. The activation to each image is labeled above the image and indicated by the color of the margin. **d)**, The top 5 images among 10,000 random images from ImageNet (ILSVRC12 dataset, $>$ 1.4 M images). The number of random images is matched to the number of images presented during optimization. The top image in all $>$ 1.4 M images is shown in Fig 2b.

I can’t figure out what image is in Figure 2b, as I don’t see a “b” there..oh - you have swapped figures 2 and 3 by mistake.

## XDream can find strong stimuli for neurons

XDream has three key components: an image generator representing the search space; an objective function given by the activation of the target unit guiding the search; and

an optimization algorithm performing the search (Fig 1a). In each *generation*, the generator creates images from their latent representations (*codes*), the target unit activation is evaluated for each of the generated images, and the optimizer refines the codes based on the measured activation values. Initialized randomly (examples shown in Fig 1a), the algorithm is iterated for 10,000 total image presentations, a relatively small number that comprises $< 1\%$ of ImageNet and that is accessible in a typical neuroscience experiment. Critically, the algorithm does not use any prior knowledge about the architecture or weights of the target model.

Can you really hold a stick for that long?

An example experiment with unit 1 in the output layer (layer fc8) of CaffeNet is shown in Fig 1b. In 500 generations of 20 images each, the activation of the target unit increased rapidly and saturated at approximately generation 300. Fig 1c shows example images at a few generations (log-spaced to show a range of activations), depicting the evolution of the images from the initial noise pattern to the final image.

How strong was the activation achieved by XDream-generated images? We compared the best image in the last generation (the *optimized image*) to images from ImageNet. Unit 1 in fc8 was trained to be a ”goldfish” detector. Correspondingly, when we randomly sampled 10,000 images from ImageNet, the best images are photos of goldfish (Fig 1d). The highest activation value observed in this random sample was 30.67. The best image from ImageNet for this unit was a picture of a goldfish and elicited an activation of 40.55 (Fig 2b). Consistent with S1 Fig, the best image found by random sampling produced a much lower activation value than the best example in ImageNet. In comparison, the optimized image generated by XDream elicited an activation of 72.42. In other words, using a limited number of presentations, XDream generated images that elicited higher activation than any natural image from ImageNet. We refer to such images with relative activation $> 1$ as *super stimuli*.

I think you mean Figure 3.

**Fig 2. XDream generalizes across layers, architectures, and training sets.** **a)**, Violin plot showing the distributions of relative activation (activation of optimized stimulus relative to highest activation in $> 1.4$ M ImageNet images) over 100 randomly selected units per layer. For each target model, we investigated early, middle, late, and output layers (see Table 2 for the specific layers). The violin contours indicate kernel density estimates of the distributions, white circles indicate the medians, thick bars indicate first and third quartiles, and whiskers indicate $1.5\times$ interquartile ranges. For comparison, grey boxes (interquartile ranges) and lines (medians) show the distribution of maximum relative activation for 10,000 random ImageNet images. The horizontal dashed line corresponds to the best ImageNet image. **b)**, Optimized (top row) and best ImageNet (bottom row) images and activations for 10 example units across layers and architectures. For output units, corresponding category labels are shown below the images.

## XDream can efficiently search a large and diverse stimulus space to find the ground truth optimal stimulus

Is XDream limited in the kind of images it can find? This is an essential question for the utility of XDream. An analysis of this question was presented in the supplement to [1], but the question is relevant here so we discuss the analyses in the current context using slightly different data. Because XDream optimizes in the latent space of a generative network, a first constraint is the range of images that can be created by the generative network. It is hard to quantify what fraction of all possible images is represented by a generative network. Instead, we qualitative assessed the expressiveness of the generative network by challenging it to synthesize diverse, arbitrarily selected target images (Fig 3, row 1). To find synthetic images that approximate the target images, we used two

heuristic methods: 1) iteratively optimizing an image code to minimize the pixel-wise difference between the generated and target image (labeled “opt”); 2) directly using CaffeNet fc6 representation of the image as the image code, because the generative network was originally trained to invert this representation (labeled “ivt”; see **Methods** for details of both methods). Fig 3, rows 2–3 show that the generative network is able to represent, at least approximately, all the tested target images.

Again, this is figure 2 in this document.

**Fig 3. The image generator can approximate arbitrary images, and XDream can find these images using only scalar distance as a loss function.** This figure reproduces Supplementary Figure 1 in [1]. The generative network is challenged to synthesize arbitrary target images (row 1) using one of two encoding methods, “opt” (row 2) and “ivt” (row 3; **Methods**). In addition, XDream can discover the target image efficiently (within 10,000 test image presentations) by using the genetic algorithm to minimize the mean squared difference between the target image and any test image as a loss function, either in pixel space (row 4) or in CaffeNet pool5 representation space (row 5).

Not only does XDream need to represent diverse images, it must also efficiently find those images (i.e., in a reasonable number of queries). When investigating the activation of units in a target model as in Fig 1, we do not know what the ground truth global optimum image is. To evaluate whether XDream is in principle capable of finding the global maximum, we considered a simple toy model where we know the ground truth best image by construction. For a target image, we minimized the mean square difference between that image and any input image, computed using either the pixel-level representation of the image (Fig 3, row 4), or, separately, the CaffeNet pool5 layer representation of the image (Fig 3, row 5). With both representations, XDream was able to uncover an image resembling the original using only 10,000 image presentations. The optimized images generated by XDream were not perfectly identical to the ground truth target. At least part of the remaining differences could be attributed to the loss function: Pixel-wise loss is known to lead to excessive smoothing, and pool5 loss is expected to lose some features and spatial information due to pooling operations and ReLU activations in preceding layers.

These results show that XDream ~~has~~ is highly “expressive”—i.e., it can generate a very large and diverse set of images ~~can be generated~~ —and suggest that it is in principle possible to reach close to the global maximum. Of note, these results do *not* show that *any* image can be generated, nor do they provide a proof of convergence to the global maximum for all visual neurons.

## XDream generalizes across layers, architectures, and training sets

The default generative network used in XDream was trained to invert the internal representations of CaffeNet layer fc6, which was in turn trained on ImageNet [9]. Can ~~Could~~ this generator allow XDream to generalize to other network layers, architectures, and training sets? If XDream is specific to certain layers and architectures, or specific to ImageNet-trained networks, this may limit its applicability to real neurons.

We first assessed whether XDream could extrapolate to other layers in CaffeNet by selecting 100 units respectively from the early, middle, late, and output layers of CaffeNet (Fig 2a). XDream was able to find optimized images that are better than the best randomly selected images across all layers ($p < 10^{-16}$, FDR corrected for 28 tests in this section). The optimized images were also significantly better than the best images in ImageNet across all units ($p < 10^{-9}$, FDR corrected for 28 tests in this

The comment starting with “but this was not…” here is confusing. Delete it here and don’t bring it up until you get to Figure 5. In fact, I think I would delete the whole sentence.

-> late hidden layer? Or output layer?

section). The highest relative activation was obtained for the late layer, but this was not a result of using the fc6-based generative network, a possibility we tested in Fig 5.

Next, we tested 100 units from 4 layers each from 5 different network architectures: ResNet-v2 152- and 269-layer variants [30], Inception-v3 [31], Inception-v4, and Inception-ResNet-v2 [32]. These models were all trained on ImageNet. XDream was able to generate better images than the best random images for the vast majority of units across all layers and architectures (Fig 2a; $p < 10^{-8}$ across layers) except the early layer of Inception-v3 ($p = 0.2$) and Inception-ResNet-v2 ($p = 0.09$). With the same exceptions, XDream generated super stimuli for all tested layers ($p = 0.01$ for the early layer of Inception-v4, $p = 2 \times 10^{-4}$ for the middle layer of Inception-ResNet-v2, and $p < 10^{-9}$ for all other layers). Example optimized images for units in different layers and architectures are shown in Fig 2b. Notably, in all networks, the late layer could be driven to higher relative activation than the other layers, suggesting that the relatively high optimized activation may be a property of layers at this relative processing stage in the network.

In Figure 2b, it would be more useful to show early, middle and late layer images, not just output images for the non-caffe architectures.

Finally, we tested a network with the same architecture as CaffeNet but trained on a different dataset, PlacesCNN [24]. PlacesCNN also contains photographic images, but they depict scenes rather than salient objects. Again, XDream was able to find super stimuli across layers in this network, even though XDream had no accesss to the corresponding training images (Fig 2a, last four distributions; $p < 10^{-6}$ across layers).

These results show that XDream can efficiently create images that trigger high activations without making any assumptions about the type of images a unit may prefer and without any knowledge of the target model architecture or connectivity, suggesting that XDream may well generalize to biological neurons. Furthermore, XDream generalizes across layers in a ConvNet, which have been shown to roughly correspond to areas along the ventral visual stream [17, 21, 22], suggesting that XDream may also generalize to several ventral stream areas. Consistent with this, initial results from [1] indicated that XDream can find optimized stimuli for V1 as well as IT neurons.

## XDream is robust to different initial conditions

XDream starts the search from an initial generation of image codes. In Fig 2, we always initialized the algorithm using the same set of 20 random image codes, 6 of which are shown in Fig 1a. In [1], XDream was initialized with random Portilla & Simoncelli textures. Does the choice of initial conditions affect the results? To address this question, we first tested how much the particular choice of random initial codes matters. For each target unit, we repeated the experiment using 10 more different random initializations and compared the optimized relative activation to that of the original random initialization. Different initial conditions produced slightly better or worse relative activation values centered around a mean difference of 0, and the standard deviation was lower than 10% of the activation values (Fig 4a). Similar activation values notwithstanding, the optimized images were different on a pixel level (Fig 4b); they may comprise an ”invariance manifold” for each neuron that contains similar but not identical images eliciting comparable activation values (see Discussion).

This is somewhat surprising. I had a similar idea many moons ago (using the GA over a vocabulary of image generating functions), and expected that there may not be a convex region containing the optimal stimuli - rather, I expected that there would be multiple optimal stimuli. My research didn’t go anywhere as the basis functions available at the time were not adequate. I think most neuroscientists expect that there will be a single optimal stimulus. There may well be an invariance manifold, but it needn’t be connected. Can’t you simple interpolate between the codes you found to see if points in between also are near optimal?

Next, we tested whether there are particularly good or bad ways of choosing the initial stimuli. We selected, separately for each target unit, the 20 ImageNet images that led to the highest, middle, and lowest activation values and used those images to form the initial population (Fig 4c). To convert images into image codes comprising the initial population, we used either the “opt” or the “ivt” method (**Methods**). Initializing with better or worse natural images did not improve the optimized images in the conv2 layer ($p = 0.87$ and 0.19 for “opt” and “ivt”, respectively, FDR-corrected for 8 tests in this and the next sentence). In higher layers, initializing with the best natural images led to slightly higher relative activation values (Fig 4c; Table 1; $p < 5 \times 10^{-3}$ for

**Fig 4. Comparison of different initializations. a,b)**, Effect of random initializations. **a)**, Top, distributions of fractional change in optimized activation if one random initialization is changed to 10 different random initializations. Bottom, standard deviation in optimized activation across 10 different random initializations, normalized to the mean activation. Error bars indicate standard deviation across different units. **b)**, Optimized images from different initializations for 3 example units in the output layer (one unit per row; 6 examples shown). Activation values are shown above each image. **c)**, Good versus bad initializations. For each target unit, its best, middle, or worst 20 images from ImageNet were used as the initial generation. The images were converted to the image code space using either an optimization method ("opt") or an inversion method ("ivt"; **Methods**). Left to right within the opt and ivt groups are results from initialization with the best, middle, and worst 20 images. Random initialization is shown for comparison. The open and solid violins show the distributions, in the first and last generation respectively, of relative activation over 100 units in each layer.

"opt" and $p < 10^{-10}$ for "ivt" across layers). However, initializing with either the middle or worst natural stimuli were not different from initializing with random codes ($p \geq 0.15$, FDR-corrected for 8 tests), except for layers fc6 ($p = 0.0014$) and fc8 ($p = 2 \times 10^{-15}$) with the "ivt" method. In an actual neurophysiology experiment, it is unlikely that the investigator would know such good stimuli comparable to the best of 1.4 M images. Therefore, initializing randomly seems reasonable.

**Table 1. Effect of using good vs. bad initialization.**

| Encoding alg. | Measure | Layer | | | |
|---|---|---|---|---|---|
| | | conv2 | conv4 | fc6 | fc8 |
| opt | slope | 0.010 | 0.037 | 0.047 | 0.056 |
| | p-value | 0.87 | 0.004 | 0.004 | $7 \times 10^{-5}$ |
| ivt | slope | 0.044 | 0.113 | 0.241 | 0.353 |
| | p-value | 0.19 | $7 \times 10^{-11}$ | $4 \times 10^{-22}$ | $5 \times 10^{-77}$ |

Any idea why there is a gradient of the slopes from left to right?

~~Table notes Phasellus venenatis, tortor nec vestibulum mattis, massa tortor interdum felis, nec pellentesque metus tortor nec nisl. Ut ornare mauris tellus, vel dapibus arcu suscipit sed.~~

For each unit, the 20 best, middle, and worst images from ImageNet, as ranked by that unit, were used to initialize the genetic algorithm. The images were converted to image codes using one of two encoding algorithms, "opt" or "ivt" (see **Methods**). "Slope" quantifies the improvement in relative activation (median across 100 random units each layer) if a better initialization is used (worst $\rightarrow$ middle or middle $\rightarrow$ best). Concretely, the slope is the linear regression coefficient with the independent variable being {0,1,2} for {worst, middle, best}, respectively. The "p-value" indicates whether there is a statistically significant difference initializing by {worst, middle, best} images (one-way ANOVA, FDR-corrected).

To summarize, initializing the algorithm with different random conditions resulted in only a small variation in the optimized image activation, and the images were similar although not identical at the pixel level. Initializing with prior knowledge has little to no effect on the optimized image activation, unless the seed is comparable to the best image in $\sim 1$ M images and only in later layers.

Use a reference, rather than saying “the paper.” Or do you mean in *this* paper? How about “In the previous experiments, we used Caffe fc6 to generate images. Here, we look at the effect of using different image generators.”

## Image generators that use high-level representations work equally well

An essential component of XDream is the image generator. In other experiments in the paper, we used a generative network based on CaffeNet fc6 representations [9]. Here, we examine whether the choice of image generator matters for the performance of XDream, and whether the answer depends on the target unit. We hypothesized that image generators based on pixels or low-level representations would not work well, and high-level features may be required for efficient optimization. To test this hypothesis, we evaluated the family of DeePSiM generators, which are generative neural networks trained to invert each layer of CaffeNet respectively [9]. The 8 image generators are all of similar depth (11–13 layers) and architecture (feedforward with no bypass or recurrent connections), although image generators trained on higher layers have more parameters due to having more convolutional filters and, in the case of fc-layer generators, having fully-connected layers. In addition to generative neural networks, we also tested a control image generator where the image was directly parameterized by the flattened pixel array. Therefore, searching the representational space of this generator is equivalent to brute-force search of possible images. As target units, we tested early, late, and output layers of CaffeNet, as well as the output layer of Inception-ResNet-v2 to evaluate cross-architecture generalization.

?? What does that mean?

Using different image generators had a statistically significant effect on optimized activation in all layers ($p < 10^{-24}$, FDR corrected for 4 tests in 4 layers). Higher layer-based image generators appeared to work better than lower layer-based ones for all target model layers except CaffeNet conv2 (Fig 5). One the other hand, the difference appeared small among high-level image generators. The generators deepsim-pool5, deepsim-fc6, and deepsim-fc7 performed similarly in CaffeNet conv2 and fc8 layers ($p = 0.3$, FDR corrected for 4 tests), whereas deepsim-pool5 and deepsim-fc6 were equivalent for CaffeNet fc6, fc8, and Inception-ResNet-v2 classifier layers ($p = 0.09, 0.12, 0.71$ respectively, FDR corrected for 32 tests comparing each generator to deepsim-fc6 in each target layer). These results indicate that the image generator does not need to be tailored to the target model unit, consistent with the ability of XDream to generalize across architectures demonstrated in Fig 2. The pixel-based image generator, compared to generative neural networks, worked more poorly in all target layers other than CaffeNet conv2 except when compared to deepsim-norm2 ($p = 1$ compared to deepsim-norm2; $p > 0.14$ compared to other generators in CaffeNet conv2; $p < 10^{-4}$ in all other comparisons; FDR-corrected for 32 tests comparing each generator to raw-pixel in each target layer). These results are consistent with the hypothesis that it is beneficial to use an image generator based on higher-level image features.

**Fig 5. Comparison of image generators.** We tested each of the family of image generators from [9] as the image generator in XDream, together with a generator representing the image as the raw pixels. Format of the plot is the same as in Fig 2a.

**but the genetic**

## Different optimization algorithms can be incorporated into XDream, but genetic algorithm consistently works well

Another important component of XDream is the optimization algorithm. The results shown thus far were based on using a genetic algorithm as the optimization algorithm, a choice inspired by previous work [10–12]. Here, we compared the genetic algorithm to two additional algorithms, a naïve finite-difference gradient descent algorithm (FDGD; **Methods**) and Natural Evolution Strategies (NES; [26], **Methods**). NES has been used in a related problem [27]. FDGD and NES were significantly worse than the

The noiseless vs. noisy colors in the key for this image don't correspond to all the plots in Figure 6 - just state that noisy is on the right and noiseless is on the left.

genetic algorithm in CaffeNet conv2 ($p < 10^{-13}$ for FDGD and NES, FDR corrected for 20 tests here and in the stochastic condition in the next section) and conv4 layers ($p < 10^{-3}$). Yet, both FDGS and NES were significantly better than the genetic algorithm in CaffeNet fc6 ($p < 10^{-16}$), fc8 ($p < 10^{-16}$), and Inception-ResNet-v2 classifier layers ($p < 10^{-12}$; Fig 6).

**Fig 6. Comparison of optimization algorithms and their robustness to noise.** We compared 3 gradient-free optimization algorithms (**Methods**): genetic algorithm, finite-difference gradient descent (FDGD), and Natural Evolution Strategies (NES; [26]). We also considered their robustness to noise in the objective function by adding Poisson noise to activation values (**Methods**). Left and right half of each violin corresponds to noiseless and noisy units, respectively. Dashed lines inside the violins indicate quartiles of the distribution. Otherwise, format of the plot is as in Fig 2a.

## XDream is robust to noise in neuronal responses

An important difference between model units and real neurons is the lack of noise in model unit activations. Upon presenting the same image, model units return a deterministic activation value; in contrast, in biological neurons, the same image can evoke different responses on repeated presentations (even though trial-averaged response may be highly consistent; see [33]). To test whether XDream could still find super stimuli with noisy units, we implemented a simple model of stochasticity in the units by using the true activation value to control the rate of a homogeneous Poisson process, from which the "observed" activation value on a single trial was drawn (**Methods**). Homogeneous Poisson processes have been used extensively to model stochasticity in cortical neurons [34].

As expected, performance deteriorated when noise was added (Fig 6, noisy condition). However, XDream was still able to find optimized stimuli that were better than random exploration for most layers ($p < 10^{-10}$ for all tested layers except $p = 0.19$ for CaffeNet fc8, FDR-corrected for 5 tests) and was also able to find super stimuli for some layers ($p < 10^{-5}$ for CaffeNet conv4 and fc6 layers; $p = 0.069$ for CaffeNet conv2 layer; FDR-corrected for 5 tests), although it was not able to find super stimuli for most units in CaffeNet fc8 layer and Inception-ResNet-v2 classifier layer ($p = 1$).

Noise in the unit activations affected different optimization algorithms to different extents. The genetic algorithm was at least as good as, and often superior to, both alternatives optimization algorithms when considering noisy units. The NES algorithm performed similarly well as the genetic algorithm in CaffeNet fc8 layer and Inception-ResNet-v2 classifier layer ($p = 0.03$ and 0.65, respectively), but was worse in the other 3 tested layers layers ($p < 10^{-14}$). The FDGD algorithm was particularly sensitive to noise, performing worse than the genetic algorithm in all layers tested ($p < 10^{-6}$) and frequently failing to find good stimuli.

I expected that in the conclusion, you would give some recommendations for using the software - in particular, recommended hyper-parameters. Maybe in the supplementary material, or in a Table here?

# Discussion

It is challenging to study the stimulus features encoded by visual cortical neurons, because the number of possible images is beyond astronomical while experimental time with a neuron is limited. In this work, we thoroughly characterize a method, named XDream, that combines an image generation algorithm with closed-loop optimization to directly use neural activity to search image space and find preferred stimuli. Using units in artificial neural networks as models of neurons in the brain, and with realistic constraints, we systematically evaluated the performance of XDream. We found that XDream can efficiently find images that trigger high activations, often higher than that from even the best in over a million natural images (Fig 1). XDream can generate a diverse set of stimuli and, in one scenario where the global maximum is known by construction, can find images approximating that maximum (Fig 3). XDream can generalize across early and late processing stages, across widely different architectures, and also across different training sets, which resemble different developmental environments (Fig 2). Furthermore, XDream is robust to different initial conditions (Fig 4) and to noise in unit responses (Fig 6).

really? This seems like a stretch.

In computer science literature, activation maximization is a well-known approach for visualizing features represented by units in a ConvNet [13, 20, 35–37]. However, the techniques are only applicable to networks that provide optimization gradients. In other words, perfect knowledge of the architecture and weights is assumed. Clearly, such requirements are not met in current neuroscience experiments. XDream extends the idea of feature visualization to situations where perfect knowledge of the underlying architecture is not available by using gradient-free optimization algorithms.

in THE computer science literature

Another approach that has inspired the current work is to use a genetic algorithm to search a parametrically-defined stimulus space [10–12]. XDream extends this idea by using a more diverse stimulus space learned by a generative neural network, which does not require prior knowledge or intuitions about the tuning properties of the neurons under study. In addition, we frame the approach more broadly, incorporating additional image generators and optimization algorithms.

Recently, several other studies have focused on similar goals to the ones in XDream, but with a different approach [21, 22, 38, 39]. In that approach, a ConvNet-based model is first fitted to predict neuronal firing responses. Then, standard white-box activation maximization techniques are applied on the ConvNet model. The relation between this approach and XDream is similar to the relation between the so-called "substitute model" approach and what, in comparison, we may call a "direct" approach, in computer science research on black-box adversarial attack. There, studies have found that the direct approach is both free of transferability problems (because no substitute model is involved) and more sample efficient [27, 40]. In this light, when comparing the substitute-model approach and the direct approach for studying neural coding properties, sample efficiency is an important consideration, as is test case performance. Recent preliminary results suggest that some ConvNet-based models do not fully extrapolate to images very different from those used during training [1, 21]. If so, these models may not be adequately guiding the exploration of image space, of which training images can only represent a small fraction.

reword to avoid repeating the word "approach". In fact, the word "approach" appears 7 times in this paragraph. Try substituting synonyms here and there, e.g., "method", "technique". Maybe it is just me that is bothered by this.

The performance of XDream is robust to many design and parameter choices in the algorithm. The use of the genetic algorithm and several related high-level generative networks [9] are adequate and perhaps ideally suited for this family of tasks. In addition, empirically optimized hyperparameter values are listed in S1 Table. The robustness of the algorithm indicates that it is unlikely specific parameters will drastically change performance, and suggests that there is no need to tailor parameters to specific neurons, areas, or species. Nevertheless, it may be possible to further fine-tune the parameters to the neurons under study.

On repeated runs from different initializations, XDream finds related but not identical images that trigger similar activation values (**Figure 4b**). We speculate that there may be a whole "invariance manifold" of related images that elicit similar responses in any given unit. Indeed, certain neurons are thought to be approximately invariant to such image transformations as position and scale changes. The notion of an invariance manifold is consistent with models of neurons as encoding particular image features and inconsistent with models of neurons as representing a single "ideal" image.

The results presented here are based on maximizing activation values, whereas the results shown in [1] are based on maximizing spike counts. While activation values and firing rates are commonly-used proxies for internal representation in machine learning and neuroscience, respectively, they are not the only possible objective functions to maximize. One could design objective functions to study other putative neural codes, such as pooled activation across multiple units, sparseness of the representation across units, patterns of population firing, correlated firing, synchronized firing, local field potentials, etc. XDream is agnostic to the underpinning of the objective function as long as it is image-specific and can be computed in real time. Thus, the same algorithm can be readily applied to investigate different putative neural coding mechanisms.

It is not practical to study the responses of biological neurons to millions of images and a wide variety of different algorithms as done in the current study. On the other hand, although we tested state-of-the-art models of neurons along the ventral visual cortex, these models cannot replace actual neurons. The fact that XDream extrapolates across layers, architectures, and models trained on different datasets bodes well for it to extrapolate to different ventral stream areas and even visual cortices of different species, but experiments with biological neurons are necessary to evaluate how well XDream will actually generalize to different neuronal types, brain areas, and species.

In summary, the XDream method is able to discover preferred features of visual units without assuming any knowledge about the structure or connectivity of the system under study. Exhaustive search is impractical for most neurophysiological studies, while random search is inefficient. XDream can search the stimulus space efficiently, requiring only on the order of a few thousand queries, within reach of common experimental paradigms. Thus, XDream can be a powerful tool for elucidating the tuning properties of neurons in the brain. Given its minimal assumptions and good generalization, XDream can be readily applied to study a variety of visual areas in different species, even where there is no prior knowledge about the neuronal preferences. Furthermore, we speculate that the general framework of XDream can be extended to other sensory domains for which good generative networks can be built, potentially including sounds, language, and music.

# Materials and methods

## Experimental design and statistical analyses

We tested XDream on units in state-of-the-art convolutional neural networks (ConvNets) pre-trained on classification tasks. These networks are currently the best class of models of visual neurons [15–17]. When substituting *in silico* units for visual neurons, we considered the *activation* of a unit to a given image as analogous to the firing rate of a real neuron to a picture; this activation provides the objective function used by the optimization algorithm to search for preferred images. Further, we considered a layer in the models as analogous to a cortical area. We treated model layers as the units of comparison and report statistics across 100 randomly selected units each layer, unless otherwise noted. When comparing two conditions for the same 100 units, we used a Wilcoxon signed-rank test. When comparing multiple conditions

(in Fig 4 and Fig 5), we used one-way ANOVA. We considered one-way ANOVA sufficient because the distributions were approximately normal, one-way ANOVA is conservative when there is deviation from normality, and one-way ANOVA quickly converges to normal-case power while the sample sizes were reasonably large. For brevity, we only cite the corresponding p-values and do not mention the type of test in text, but it should be clear from context. For multiple comparisons made to answer the same question, P-values were corrected for false discovery rate using the Benjamini-Hochberg procedure with an alpha level of 0.01; the number of conditions corrected will be mentioned in text with the first P-value in the group.

## Target models and layers

We selected several state-of-the-art ConvNets as target models, many of which have been shown to be reasonably good models of primate visual neuron responses [16]. In each model, we tested what are approximately the early, middle, and late processing stages as well as the output layer; these layers roughly correspond to early-to-late processing stages in the ventral visual cortex [17, 21, 22]. Table 2 specifies which architectures and layers were used. One hundred (100) units were randomly selected from each layer. For convolutional layers, only the center spatial position was selected for each feature channel. All the networks were trained on the ImageNet dataset [23] except PlacesCNN, which was trained on the Places-205 dataset [24].

**Table 2. Target networks and layers.**

| Network | Layers | | | |
|---|---|---|---|---|
| | early | middle | late | output |
| caffenet | conv2 | conv4 | fc6 | fc8 |
| resnet-152-v2 | res15_eletwise | res25_eletwise | res35_eletwise | classifier |
| resnet-269-v2 | res25_eletwise | res45_eletwise | res60_eletwise | classifier |
| inception-v3 | pool2_3x3_s2 | reduction_a_concat | reduction_b_concat | classifier |
| inception-v4 | inception_stem3 | reduction_a_concat | reduction_b_concat | classifier |
| inception-resnet-v2 | stem_concat | reduction_a_concat | reduction_b_concat | classifier |
| placesCNN | conv2 | conv4 | fc6 | fc8 |

For each network, 4 layers from what is roughly the early, middle, late stages of processing, together with the output layer before softmax, were selected as targets. PlacesCNN has the same architecture as CaffeNet but is trained on the Places-205 dataset [24]. CaffeNet is as implemented in `https://github.com/BVLC/caffe/tree/master/models/bvlc_reference_caffenet`, PlacesCNN as in [24], and the remaining as in `https://github.com/GeekLiB/caffe-model`.

**use tinyurl to shorten this.**

## Image generators

An image generator is a function that outputs an image given some representation of that image (an *image code*) as input. We tested the family of DeePSiM generators developed in [9]; they are generative adversarial networks trained to invert each layer of AlexNet [25]. The pre-trained models are available on the authors' website at `https://lmb.informatik.uni-freiburg.de/people/dosovits/code.html`. We used the image generator inverting the fc6 layer by default except in Fig 5, where we compared different generators.

## Optimization algorithms

An optimization algorithm in the context of XDream is a function that iteratively proposes a set of image codes (real-valued vectors) $\boldsymbol{c}_i, i = 1, \ldots, n$, or *codes* for short,

and then uses their corresponding *fitness* values $y_i, i = 1, \ldots, n$ (i.e., scalar activation values by a target model unit to the image associated with each code) to propose a new set of codes expected to have higher fitness. We used a genetic algorithm by default, but also considered two other algorithms: finite-difference gradient descent (FDGD) and natural evolution strategies (NES).

The genetic algorithm works as follows: Each generation consists of $n$ codes, where $n$ is the population size parameter. Their corresponding fitness values $y_i, i = 1, \ldots, n$ are transformed into probability weights $w_i = \exp((y_i - \min_i(y_i))/k)$, where $k = \mathrm{stdev}_i(y_i)/s$ is analogous to temperature in the Boltzmann equation and $s$ is the selectivity parameter (higher $s$ is analogous to lower temperature and means high fitness is more heavily favored). To create each code in the next generation (a progeny), two codes (parents) are drawn with the probability for each code to be drawn equal to $p_i = w_i / \sum_i w_i$. In our setting, the two parents do not have to be distinct. A random fraction $h$ of vector components in the progeny is drawn from one parent and $(1 - h)$ from the other, where $h$ is the heritability parameter. Finally, a fraction $r$ of the components in each progeny is subject to mutation drawn from a zero-centered Gaussian of scale $\sigma$; $r$ is the mutation rate parameter and $\sigma$ the mutation size parameter.

The finite-difference gradient descent (FDGD) algorithm works as follows: a set of $2n$ sample codes $\boldsymbol{c}_{i,\pm}, i = 1, \ldots, n$ is proposed around the current center code $\boldsymbol{c}_0$ by adding to it zero-centered Gaussian perturbation $\boldsymbol{\delta}_i$ of scale $\sigma$, where $\sigma$ is the search radius parameter. The samples are antithetic, meaning that $\boldsymbol{c}_{i,\pm} = (\boldsymbol{c}_0 \pm \boldsymbol{\delta}_i)$. The gradient estimate is then $\Delta \boldsymbol{c}_0 = \sum_i \Delta y_i \boldsymbol{\delta}_i / \|\boldsymbol{\delta}_i\|^2$, where $\Delta y_i = (y_{i,+} - y_{i,-})$ and $\|\cdot\|$ is the L1 norm. The new center is then $\boldsymbol{c}_0' = (\boldsymbol{c}_0 + \eta \Delta \boldsymbol{c}_0)$, where $\eta$ is the learning rate parameter.

The natural evolution strategies (NES) algorithm aims to maximize not $y = f(\boldsymbol{c}_0)$ at a center code $\boldsymbol{c}_0$, but the expectation $\mathbb{E}_{\pi(\boldsymbol{c})}[f(\boldsymbol{c})]$ over a search distribution $\pi(\boldsymbol{c})$. We refer the reader to [26] for motivation and derivation. The implementation is as follows: the search distribution is a Gaussian of scale $\sigma$ around the current center $\boldsymbol{c}_0$. A set of $2n$ antithetic samples, $\boldsymbol{c}_{i,\pm}, i = 1, \ldots, n$, is proposed as above. The gradient estimate for the center is $\Delta \boldsymbol{c}_0 = \frac{1}{n\sigma^2} \sum_{i,\pm} s_{i,\pm} \boldsymbol{\delta}_i$. The new center is $\boldsymbol{c}_0' = (\boldsymbol{c}_0 + \eta \Delta \boldsymbol{c}_0)$, where $\eta$ is the learning rate parameter. The scale parameter is also updated using the gradient $\Delta \sigma = \frac{1}{n\sigma} \sum_{i,\pm} s_{i,\pm} \left( \frac{\boldsymbol{\delta}_i^2}{\sigma^2} - 1 \right)$ and a separate learning rate. Note that we use the same $\sigma$ for all components in the image code and thus do not model a multidimensional Gaussian nor any covariances, different from the general case discussed in [26]. However, we do update the scale of the search distribution, different from [27]. We have tried updating separate, independent $\sigma$'s for each component in the image code, but the performance is much worse, presumably because there is too little information to reliably estimate gradients for the second moment.

## Converting images to image codes

In several cases (e.g., Fig 3, Fig 4), we needed to convert an image into an image code in the input space of the image generator. We used two heuristic methods for this purpose, "opt" and "ivt." In the "opt" method, starting with an all-zero image code, the image code was iteratively optimized using backpropagation and gradient descent to minimize the pixel-wise difference between the generated image and the target image. In the "ivt" method, because the generator was originally trained to invert this encoding [9], the fc6-layer encoding of the target image by AlexNet was used as the image code.

## Hyperparameters

To choose a set of good hyperparameters, we used a greedy algorithm that maximized performance over a small set of target units by varying one hyperparameter at a time.

To keep the computation tractable, we used 12 units total, 3 randomly chosen from the output layer of each of 4 networks: CaffeNet, ResNet-152, Inception-v2, and PlacesCNN. Starting from an educated guess of hyperparameter values, one hyperparameter was chosen at a time. Four test values were chosen around the current value with a pre-defined step size, and optimization performance was measured with the test values. The value that yielded the best performance was set as the current value. Then, another hyperparameter was chosen to be varied. The same hyperparameter was not chosen again until all others had been considered once; we call each repeat of all hyperparameters one *round*. If no hyperparameter was updated in a given round, the step size was decreased for the hyperparameter that had not been updated for the longest time. This procedure was repeated until all pre-defined, progressively decreasing step sizes for each parameter were exhausted. The final best parameter settings were used as the default values. Hyperparameters were optimized separately for each optimization algorithm, for each image generator, and for the noiseless and noisy case (described below). The hyperparameters used in this paper are listed in S1 Table and S2 Table. An example of hyperparameter landscape around the current best values can be seen in S2 Fig.

## Stochastic neuron models

Biological neurons can yield different responses upon repeated presentation of the same stimulus. To examine the effect of this variability on XDream, we artificially added noise to the unit activations. Let $y$ be the activation value of a ConvNet unit. The activation value corrupted by stochastic noise was drawn from $Y \sim \text{Poisson}(\max(0, \gamma))$, where the rate parameter $\gamma$ is analogous to the number of spikes of a neuron and equals $y$ times a constant scaling factor. The scaling factor is necessary because the signal-to-noise ratio (SNR) of a Poisson process—in terms of mean over standard deviation—increases as the rate of the Poisson process increases, but normalized vs. unnormalized networks and different layers in an unnormalized network produce activation values of different scales. We used a scaling factor of $20/\hat{y}$, where $\hat{y}$ is the median of the max activation to 2,500 random ImageNet images, and 20 is a realistic number of spikes a biological neuron may fire to a preferred stimulus within a measurement time of 200 ms. Note that a Poisson process with $\gamma = 20$ has an SNR of $\mu/\sigma = \sqrt{20} \approx 4.5$; the SNR will be lower for less optimal images with a lower rate parameter. To simulate repeated image presentations, we simply drew multiple $Y$ values from the same Poisson distribution, with the realistic trade-off that fewer unique images could be presented given the same total number of allowed queries.

## Computing environment

The generative models were based on the caffe library [28] in Python. We have converted the models to PyTorch for convenience for future research. Links to the converted models are available on the code repository (see Code availability below). Neural network computations were performed on NVIDIA GPUs.

## Code availability

The code for XDream can be obtained directly from `https://github.com/willwx/XDream/`.

## Source of the target images in Fig 3

The leftmost 2 images were manually created; the third image was synthesized as described in [29]; the fourth image is from the ImageNet test set; the rightmost 3 images are public domain images from NASA and The Metropolitan Museum of Art.

# Acknowledgements

This work was supported by the Center for Brains, Minds and Machines funded by NSF STC award CCF-1231216 and also by NIH R01EY026025. The authors declare no competing interests.

# Supporting information

**S1 Fig. Expected maximum relative activation in response to natural images with random sampling.** We measured the max relative activation expected in two random sampling schemes. "Random" refers to picking a given number of images randomly from the ImageNet dataset (blue). "10 categories" refers to first randomly picking 10 categories out of the 1000 ImageNet categories and then picking a given number of images randomly from those categories so that the total number of images is the one indicated on the x-axis (gray). We considered 4 layers from the CaffeNet architecture. Lines indicate the median relative activation (highest activation divided by the highest activation for all ImageNet images). Shading indicates the 25th- to 75th-percentiles among 100 random units per layer.

**S2 Fig. Comparison of hyperparameters in the genetic algorithm.** In each plot, one hyperparameter was varied while the others were held constant at default values indicated by the open circles. Dots indicate the mean of relative activation across 40 target neurons, 10 neurons each in 4 layers specified in S3 Table. Blue and orange lines indicate noiseless and noisy target units, respectively. Light colored lines indicate the mean across the 10 units within each architecture and layer. Light gray shading indicates the linear portion of a symmetrical log plot, which is used in order to show zero values.

**S1 Table. Optimized hyperparameter values for the genetic algorithm.** Hyperparameters used in the experiments in this paper, obtained as described in **Methods** separately for each generative network and for noiseless and noisy targets.

| Hyperparam. | Generator | | | | | | | | | |
|---|---|---|---|---|---|---|---|---|---|---|
| | pixel | norm1 | norm2 | conv3 | conv4 | pool5 | fc6 | fc7 | fc8 | fc6 |
| Pop. size | 30 | 15 | 10 | 12 | 10 | 10 | 20 | 45 | 20 | 45 |
| Mut. rate | 0.006 | 1 | 0.5 | 0.65 | 0.9 | 0.6 | 0.5 | 0.6 | 0.2 | 0.9 |
| Mut. size | 0.16 | 1.5 | 0.7 | 0.75 | 0.75 | 1 | 0.5 | 0.3 | 0.6 | 0.5 |
| Selectivity | 2.5 | 2 | 4 | 2.25 | 2.5 | 2.5 | 2 | 1.25 | 2 | 1 |
| Heritability | 0.6 | 0.5 | 0.5 | 0.5 | 0.5 | 0.55 | 0.5 | 0.5 | 0.5 | 0.5 |
| N conserve | 0 | 0 | 0 | 0 | 0 | 0 | 0 | 0 | 0 | 7 |
| Reps. | 1 | 1 | 1 | 1 | 1 | 1 | 1 | 1 | 1 | 1 |
| Noise in target | Noiseless | | | | | | | | | Noisy |

These mutation rates seem rather high. Can you comment on that?

**S2 Table. Optimized hyperparameter values for the FDGD and NES algorithms.** Hyperparameters used in the experiments in this paper, obtained as described in **Methods** separately for the noiseless and noisy case. The generative network was always deepsim-fc6.

| Hyperparam. | Algorithm | | | |
|---|---|---|---|---|
| | FDGD | | NES | |
| N samples | 20 | 40 | 20 | 18 |
| Search radius | 1.25 | 1.15 | 1.25 | 2.25 |
| Learning rate | 1.25 | 1.5 | 1.75 | 1.2 |
| Search radius learning rate | N/A | N/A | 0.05 | 0.02 |
| Reps | 1 | 3 | 1 | 1 |
| Noise in target | Noiseless | Noisy | Noiseless | Noisy |

**S3 Table. Inferior temporal cortex-like layers.** From each layer, 10 units were randomly selected and used in hyperparameter evaluation.

| Network | Layer |
|---|---|
| caffenet | pool5 |
| resnet-101-v2 | res32_eletwise |
| placesCNN | pool5 |
| densenet-169 | concat_5_31 |

# References


1. Ponce CR, Xiao W, Schade P, Hartmann TS, Kreiman G, Livingstone MS. Evolving Images for Visual Neurons Using a Deep Generative Network Reveals Coding Principles and Neuronal Preferences. Cell. 2019;177:999–1009. doi:10.1016/j.cell.2019.04.005.

2. Kuffler S. Discharge patterns and functional organization of mammalian retina. Journal of Neurophysiology. 1953;16:37–68.

3. Hubel DH, Wiesel TN. Receptive fields, binocular interaction and functional architecture in the cat's visual cortex. The Journal of physiology. 1962;160(1):106–154.

4. Gallant JL, Braun J, Van Essen DC. Selectivity for polar, hyperbolic, and Cartesian gratings in macaque visual cortex. Science. 1993;259(5091):100–103.

5. Pasupathy A, Connor CE. Population coding of shape in area V4. Nature Neuroscience. 2002;5(12):1332–1338. doi:10.1038/972.

6. Logothetis NK, Sheinberg DL. Visual object recognition. Annual Review of Neuroscience. 1996;19:577–621. doi:10.1146/annurev.ne.19.030196.003045.

7. Desimone R, Albright T, Gross C, Bruce C. Stimulus-selective properties of inferior temporal neurons in the macaque. Journal of Neuroscience. 1984;4(8):2051–2062.

8. Tsao DY, Freiwald WA, Tootell RBH, Livingstone MS. A Cortical Region Consisting Entirely of Face-Selective Cells. Science. 2006;311(5761):670–674. doi:10.1126/science.1119983.

9. Dosovitskiy A, Brox T. Generating Images with Perceptual Similarity Metrics based on Deep Networks. In: Advances in Neural Information Processing Systems; 2016. p. 658–666.

10. Yamane Y, Carlson ET, Bowman KC, Wang Z, Connor CE. A neural code for three-dimensional object shape in macaque inferotemporal cortex. Nature Neuroscience. 2008;11(11):1352–1360. doi:10.1038/nn.2202.

11. Carlson ET, Rasquinha RJ, Zhang K, Connor CE. A Sparse Object Coding Scheme in Area V4. Current Biology. 2011;21(4):288–293. doi:10.1016/j.cub.2011.01.013.

12. Vaziri S, Carlson ET, Wang Z, Connor CE. A Channel for 3D Environmental Shape in Anterior Inferotemporal Cortex. Neuron. 2014;84(1):55–62. doi:10.1016/j.neuron.2014.08.043.

13. Nguyen A, Dosovitskiy A, Yosinski J, Brox T, Clune J. Synthesizing the preferred inputs for neurons in neural networks via deep generator networks. In: Advances in Neural Information Processing Systems; 2016. p. 3387–3395.

14. Pospisil DA, Pasupathy A, Bair W. 'Artiphysiology' reveals V4-like shape tuning in a deep network trained for image classification. eLife. 2018;7:e38242.

15. Yamins DLK, Hong H, Cadieu CF, Solomon EA, Seibert D, DiCarlo JJ. Performance-optimized hierarchical models predict neural responses in higher visual cortex. Proceedings of the National Academy of Sciences. 2014;111(23):8619–8624. doi:10.1073/pnas.1403112111.

16. Schrimpf M, Kubilius J, Hong H, Majaj NJ, Rajalingham R, Issa EB, et al. Brain-Score: Which Artificial Neural Network for Object Recognition is most Brain-Like? bioRxiv. 2018;.

17. Cadena SA, Denfield GH, Walker EY, Gatys LA, Tolias AS, Bethge M, et al. Deep convolutional models improve predictions of macaque V1 responses to natural images. PLOS Computational Biology. 2019;15(4):1–27. doi:10.1371/journal.pcbi.1006897.

18. Erhan D, Bengio Y, Courville A, Vincent P. Visualizing Higher-Layer Features of a Deep Network. University of Montreal. 2009;1341(3).

19. Szegedy C, Zaremba W, Sutskever I, Bruna J, Erhan D, Goodfellow I, et al. Intriguing properties of neural networks. arXiv. 2013;.

20. Olah C, Mordvintsev A, Schubert L. Feature Visualization. Distill. 2017;.

21. Bashivan P, Kar K, DiCarlo JJ. Neural population control via deep image synthesis. Science. 2019;364(6439). doi:10.1126/science.aav9436.

22. Abbasi-Asl R, Chen Y, Bloniarz A, Oliver M, Willmore BDB, Gallant JL, et al. The DeepTune framework for modeling and characterizing neurons in visual cortex area V4. bioRxiv. 2018;.

23. Russakovsky O, Deng J, Su H, Krause J, Satheesh S, Ma S, et al. ImageNet Large Scale Visual Recognition Challenge. International Journal of Computer Vision. 2015;115(3):211–252. doi:10.1007/s11263-015-0816-y.

24. Zhou B, Lapedriza A, Xiao J, Torralba A, Oliva A. Learning Deep Features for Scene Recognition using Places Database. In: Advances in Neural Information Processing Systems 29; 2014. p. 487–495. Available from: http://dl.acm.org/citation.cfm?id=2968826.2968881.

25. Krizhevsky A, Sutskever I, Hinton GE. ImageNet Classification with Deep Convolutional Neural Networks. In: Pereira F, Burges CJC, Bottou L, Weinberger KQ, editors. Advances in Neural Information Processing Systems 25; 2012. p. 1097–1105. Available from: http://papers.nips.cc/paper/4824-imagenet-classification-with-deep-convolutional-neural-networks.pdf.

26. Wierstra D, Schaul T, Glasmachers T, Sun Y, Peters J, Schmidhuber J. Natural Evolution Strategies. Journal of Machine Learning Research. 2014;15:949–980.

27. Ilyas A, Engstrom L, Athalye A, Lin J. Black-box Adversarial Attacks with Limited Queries and Information. In: Dy J, Krause A, editors. Proceedings of the 35th International Conference on Machine Learning. vol. 80 of Proceedings of Machine Learning Research; 2018. p. 2137–2146. Available from: http://proceedings.mlr.press/v80/ilyas18a.html.

28. Jia Y, Shelhamer E, Donahue J, Karayev S, Long J, Girshick R, et al. Caffe: Convolutional Architecture for Fast Feature Embedding. arXiv. 2014;.

29. Portilla J, Simoncelli EP. A Parametric Texture Model Based on Joint Statistics of Complex Wavelet Coefficients. International Journal of Computer Vision. 2000;40(1):49–70. doi:10.1023/A:1026553619983.

30. He K, Zhang X, Ren S, Sun J. Identity Mappings in Deep Residual Networks. Lecture Notes in Computer Science. 2016; p. 630–645. doi:10.1007/978-3-319-46493-0_38.

31. Szegedy C, Vanhoucke V, Ioffe S, Shlens J, Wojna Z. Rethinking the Inception architecture for computer vision. In: Proceedings of the IEEE conference on computer vision and pattern recognition; 2016. p. 2818–2826.

32. Szegedy C, Ioffe S, Vanhoucke V, Alemi AA. Inception-v4, Inception-ResNet and the Impact of Residual Connections on Learning. In: Thirty-First AAAI Conference on Artificial Intelligence; 2017.

33. Koch C. Biophysics of Computation. New York: Oxford University Press; 1999.

34. Gabbiani F, Cox S. Mathematics for Neuroscientists. London: Academic Press; 2010.

35. Simonyan K, Vedaldi A, Zisserman A. Deep Inside Convolutional Networks: Visualising Image Classification Models and Saliency Maps. arXiv. 2013;.

36. Olah C, Satyanarayan A, Johnson I, Carter S, Schubert L, Ye K, et al. The Building Blocks of Interpretability. Distill. 2018;.

37. Carter S, Armstrong Z, Schubert L, Johnson I, Olah C. Activation Atlas. Distill. 2019;.

38. Walker EY, Sinz FH, Froudarakis E, Fahey PG, Muhammad T, Ecker AS, et al. Inception in visual cortex: in vivo-silico loops reveal most exciting images. bioRxiv. 2018;.

39. Malakhova K. Visualization of information encoded by neurons in the higher-level areas of the visual system. J Opt Technol. 2018;85(8):494–498. doi:10.1364/JOT.85.000494.

40. Chen PY, Zhang H, Sharma Y, Yi J, Hsieh CJ. ZOO: Zeroth Order Optimization Based Black-box Attacks to Deep Neural Networks without Training Substitute Models. In: Proceedings of the 10th ACM Workshop on Artificial Intelligence and Security; 2017. p. 15–26.

Click here to download Figure Figure_1.eps

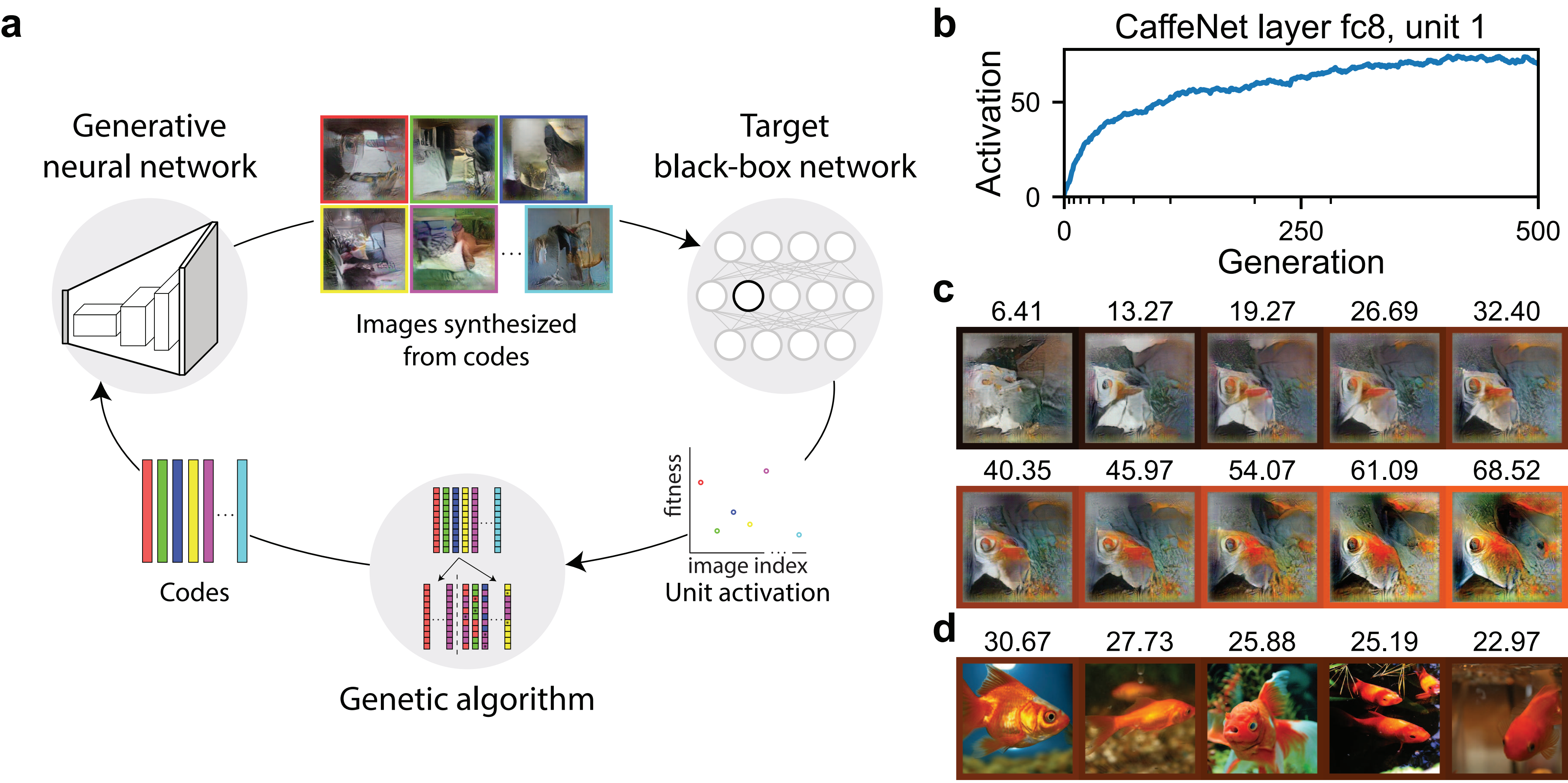

Click here to download Figure Figure_2.eps

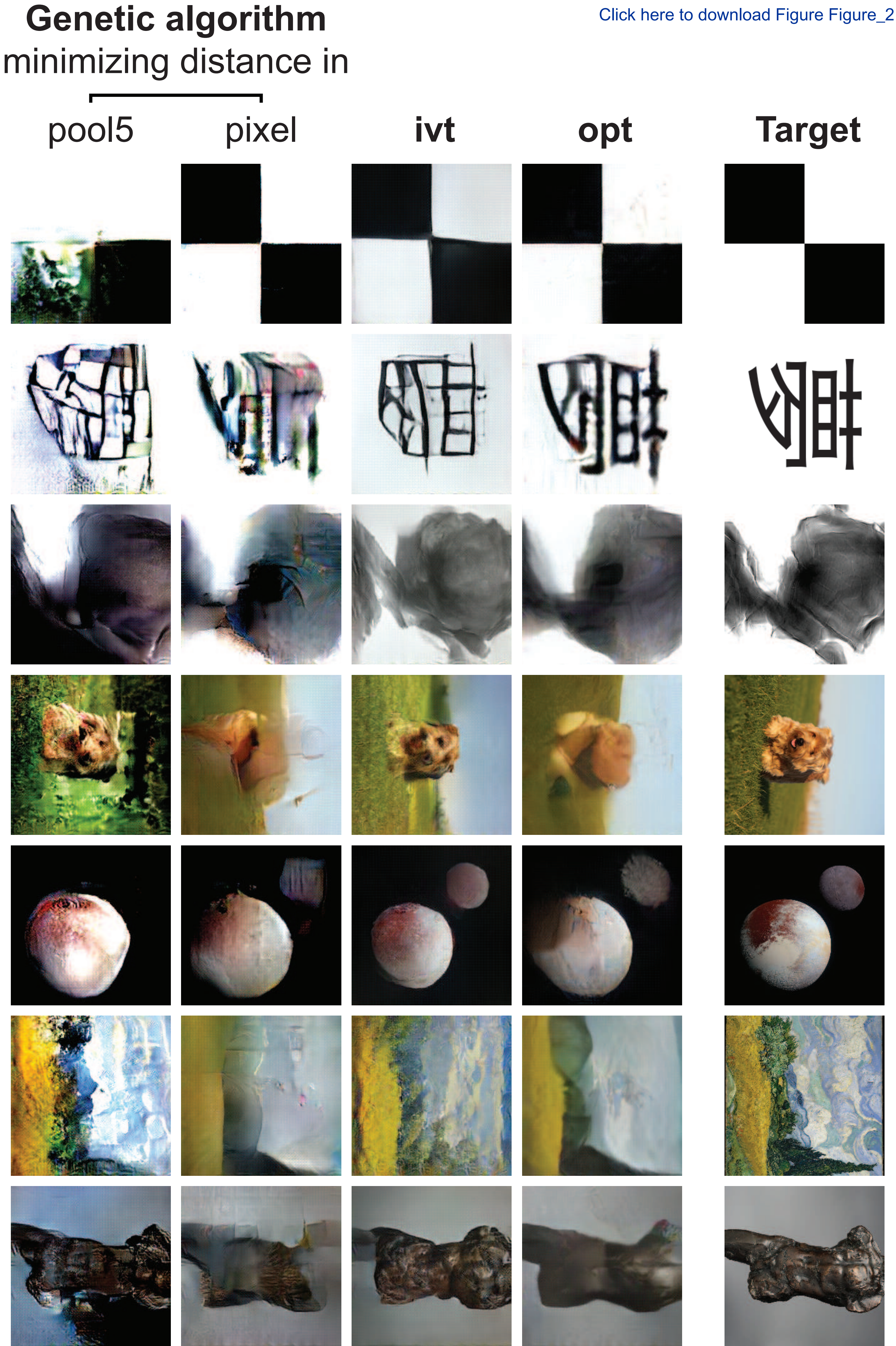

Click here to download Figure Figure_3.eps

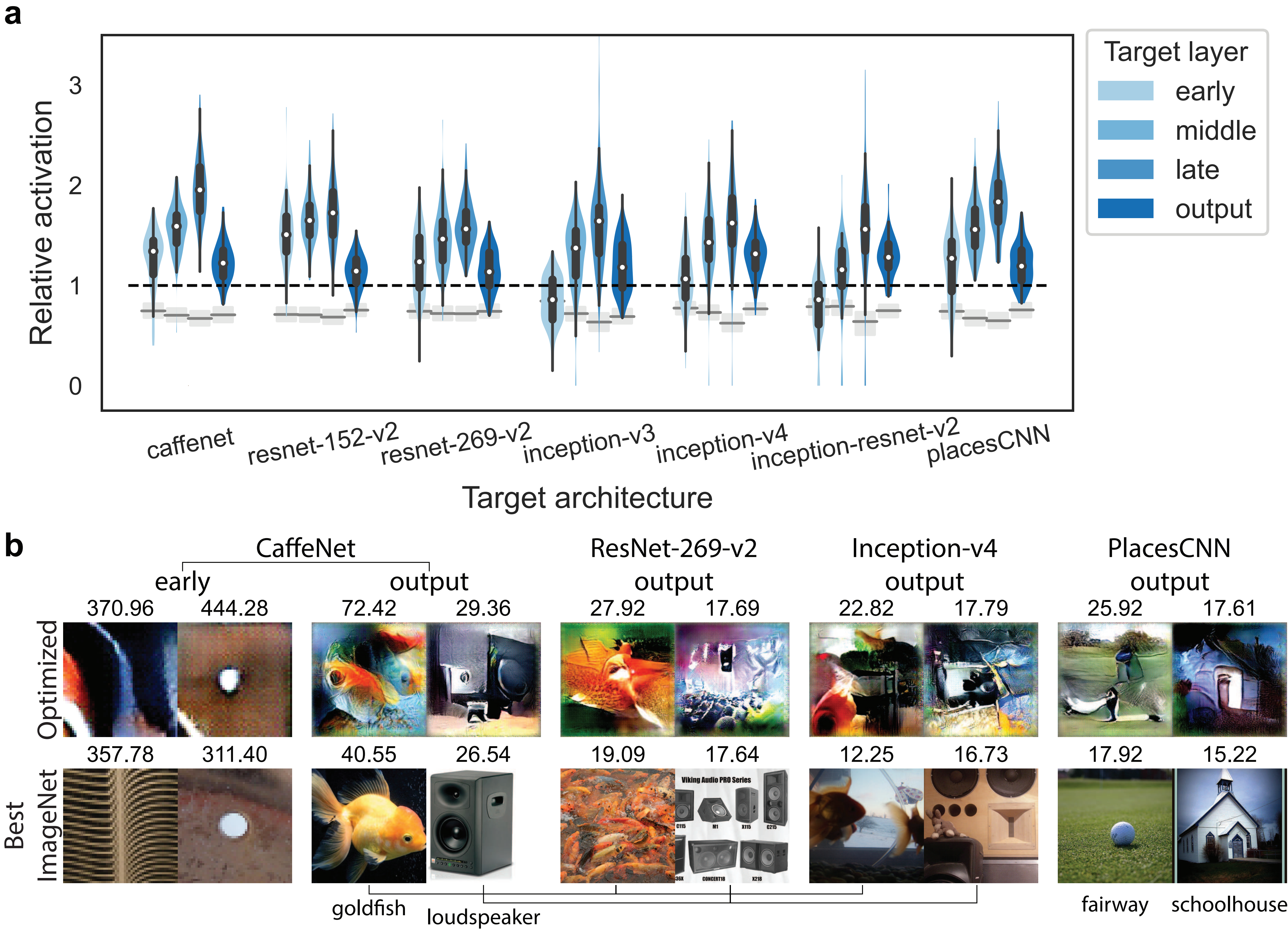

Click here to download Figure Figure_4.eps

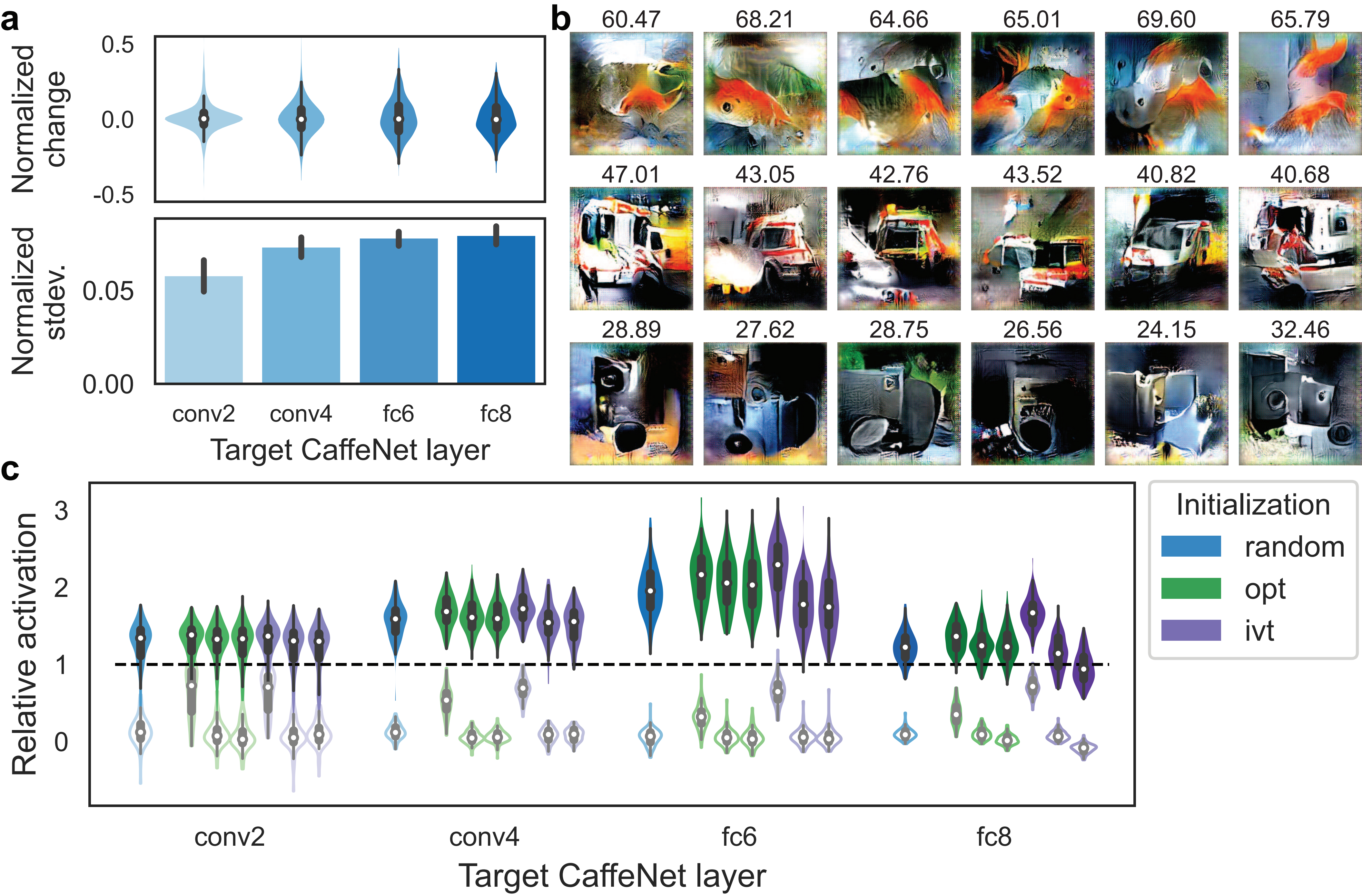

Click here to download Figure Figure_5.eps

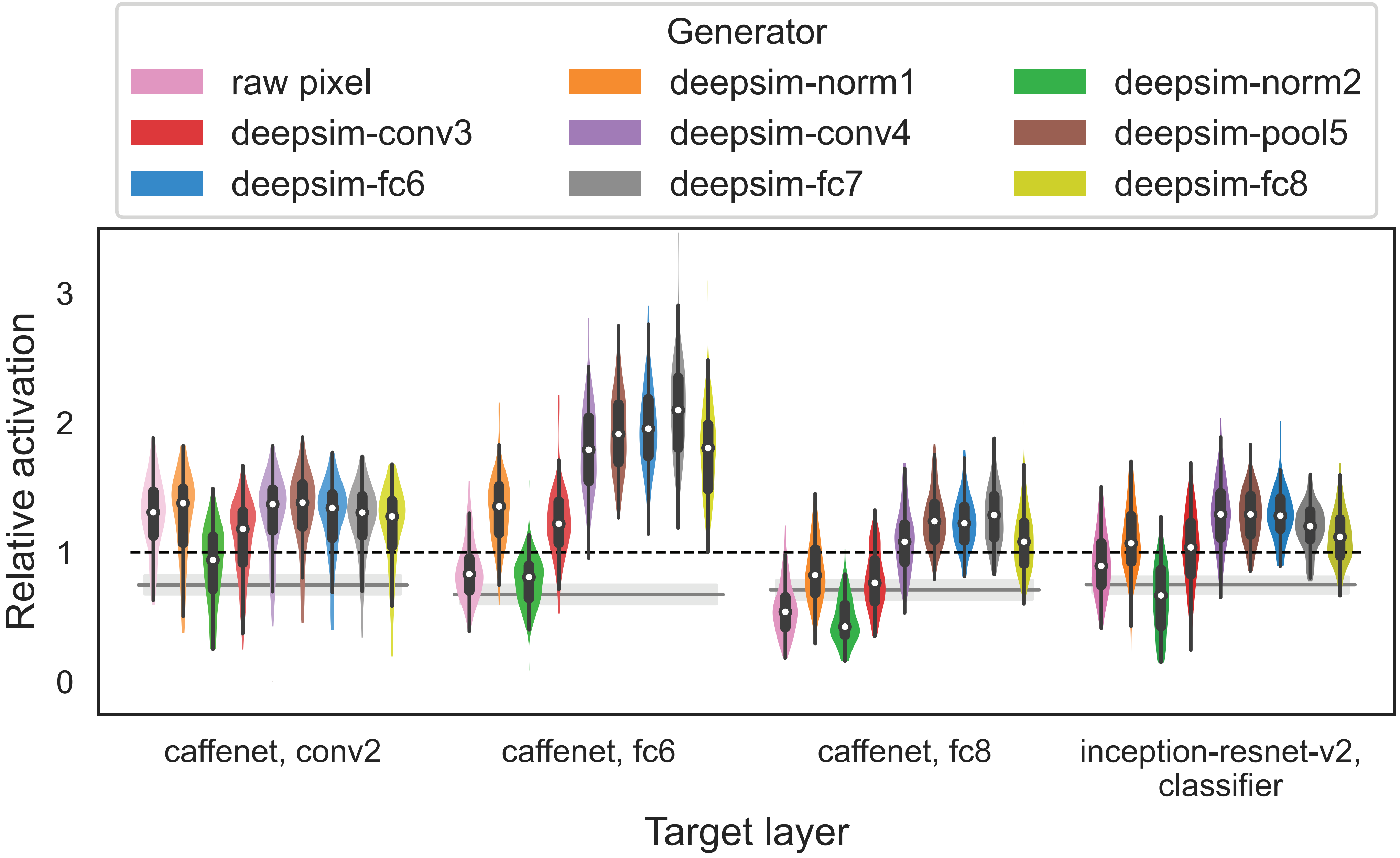

Click here to download Figure FIgure_6.eps

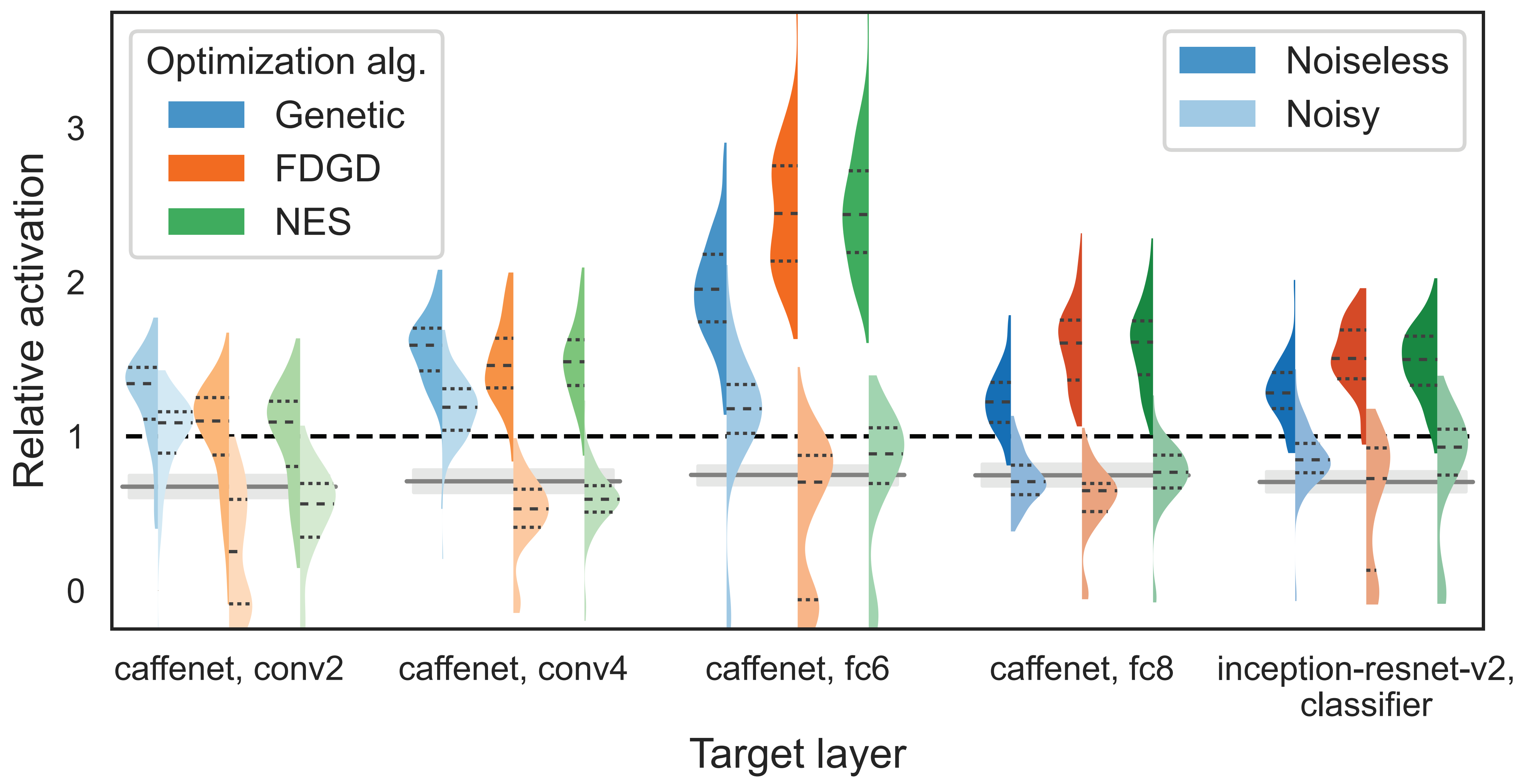

Click here to access/download
**Supporting Information**
Figure_S1.eps

Click here to access/download
**Supporting Information**
Figure_S2.eps
