# Supplementary material for: XDream: Finding preferred stimuli for visual neurons using generative networks and gradient-free optimization
Source: PLoS Comput Biol. 2020 Jun 15;16(6):e1007973. doi: 10.1371/journal.pcbi.1007973 (PMC7316361; doi:10.1371/journal.pcbi.1007973)
Supplement: Attachment — Submitted filename: response2.pdf [file pcbi.1007973.s013.pdf]

We are thankful to the reviewers for their insightful and constructive comments. We have addressed the comments point-by-point and incorporated all replies into the text.

Due to the editor's request to reformat the article to conform to the Software Article format of the journal, substantial parts of the manuscript have been moved to the supplementary material; we apologize in advance for the major changes. In the following replies, line references correspond to the clean version (no tracked changes) of the revised manuscript (line #) or supplementary material (line S#).

Reviewer 1: The authors produce an analysis of the XDream method that uses generative networks with a genetic algorithm for obtaining stimuli that will optimally drive neurons. They test this method by using it on pre-trained deep neural networks. They show that XDream can consistently find stimuli that will drive units in pre-trained networks to responses greater than those of the image set from which the networks were trained. This result is approximately independent of depth in the network and is independent of initial conditions. It is relatively robust to the choice of generative network, as long as that generator produces a "high-level" representation. These results are somewhat robust to the choice of optimization but are impacted by adding noise to the representation.

This looks at an important issue for visual neuroscience, that of finding stimuli that drive neurons in a manner that is not plagued by various experimental and theoretical biases (cf. Olshausen-Field "What is the other 85% of V1 doing?" and Carandini, et al 2005, "Do we know what the early visual system does?"). The XDream tool is an example of an emerging methodology for dealing with this problem and studying its capabilities and drawbacks is therefore important and useful to the field. The paper is clear. I recommend publication after the authors have addressed the feedback below.

Thank you for mentioning Olshausen & Field, 2005 and Carandini et al., 2005. We certainly embrace their viewpoint and now cite them in the Discussion (line S338).

1) The most important issue the authors should address is to provide some acknowledgement or discussion of the notion of optimal or "preferred" stimuli in the first place. By their methodology, they are looking for a single image that will optimally drive neurons, but this is probably not the right way to think about the coding problem, certainly not for neurons and probably not for the units in deep networks as well. The "single best image" approach is going to gloss over issues like contextual dependence. These optimal stimuli may in fact be a very small set that drives responses because they combine some "feature" for which the neuron/unit codes with exactly the right context to enhance the response. Depending on the size of this space and the nature of the context, one might wind up with an image that is not very informative of the actual coding properties of the neuron or unit. The authors use the phrase "true feature preference" in the introduction, but need to acknowledge that their method may not find it either. Furthermore, this problem is compounded in the case of real neurons, in which there are feedback and lateral inputs that

produce extra-classical effects. These effects may or may not appear in the models that the authors are testing, since they are all feedforward networks. This set of questions will almost certainly lead to the "invariance manifold" that the authors discussed.

None of this takes away from the authors' work, but users of the method should be aware of these issues.

Thank you for this insightful evaluation of the XDream method. The reviewer makes many interesting points here, which we will discuss one by one.

In terms of finding the “true feature preference,” we acknowledge here and in text that we cannot guarantee that XDream will converge to the global optimum (“Of note, these results do not [...] provide a proof of convergence to the global maximum for all visual neurons” (lines S177–179). Data such as (what is now) S3 Fig suggest that XDream can get quite close in certain cases, whereas data such as (what is now) Fig 3d, comparing random init and best-ivt init for layer fc6 and fc8, suggest that in certain cases XDream may not find the global optimum within the amount of observation allowed (10,000 image presentations).

Regarding the “right way to think about the coding problem,” we also concur that the optimal stimulus of a neuron only gives us a limited understanding of the function of that neuron in representing visual information. We focused on defining the optimal stimulus, which we believe can serve as the first step to defining stimulus parameters whose variation is encoded by that neuron (e.g., Pauspathy & Connor, 2002). This is in turn essential for understanding how a population of neurons, with such a collection of “tuning curves,” can lead to the representation of complete visual percepts (e.g., Chang & Tsao, 2017). As the discovery of visual tuning features is often serendipitous, we think XDream is useful in providing ideas of what visual properties investigators should test, both in areas whose tuning is still poorly understood, and in well-studied areas where studies may nevertheless have missed important aspects of tuning. Furthermore, we note that during the optimization process, XDream does test thousands of related images, covering the target unit's response levels both widely and densely (Fig 1c). Closer analyses of these images will likely reveal richer information about the tuning curve (e.g., properties of tuning and invariant properties) than what's reflected by the single best image.

In terms of contextual dependence, we again completely agree with the reviewer that XDream is designed to identify any and all aspects of the stimulus that will activate the neuron, including features that may be considered “context.” However, we think that the notion of context is somewhat arbitrary (in the same way that there is no sharp distinction between classical and extra-classical receptive fields). What does it mean for a neuron to distinguish between “object” and “context”? Perhaps, different stimulus features interact in a non-linear fashion, in a possibly hierarchical manner (e.g., a face neuron may have a preferred face orientation, but it will not respond to other objects in that orientation); XDream will not automatically factorize the different features, a fact that can be considered a shortcoming of the method. Still, in such a case, we believe that both features are part of the “actual coding properties” of the

neuron, and it is valuable for XDream to uncover them.

Finally, concerning recurrent (feedback and lateral) inputs that may produce extra-classical effects in real neurons, the reviewer correctly points out that they are absent from the models we have tested in the paper. Therefore, we constructed a toy model that mimics the iconic extra-classical effect of "surround suppression" (Allman et al., 1985, Annu Rev Neurosci): We took a single feature channel from the conv1 layer of AlexNet and tiled it spatially with a central, circular excitatory region and a concentric suppressive ring, creating a unit that is activated by a feature in the center but suppressed by the same feature in the surround (see the new S6 Fig). When evolving a stimulus for this unit, XDream indeed creates a center-ON surround-OFF pattern that reflects the pattern preference and matches varying receptive field sizes. In general, we think that as long as any extra-classical effect is reflected in the part of the firing rate of a neuron used for the fitness function (in particular, the relevant response time window needs to be included), and to the extent that XDream is a general method for finding stimuli that maximizes stimulus response—which appears true to the extent we have tested—XDream should be able to address the issue of recurrent, extra-classical effects. In fact, we think it is an interesting future direction to study the extra-classical tuning of ventral stream neurons by using XDream with different-sized stimuli or different response time windows.

Thank you for these questions that clarify the utility of XDream. We have added to the text summarizing all the discussion above (lines 381–396, S365–372).

2) Related to point 1, the authors should comment on the possible ethological relevance of super-stimuli. It is not surprising that such stimuli exist. Given that each unit produces a 1D parameterized function of image space, one should be able to find a point in the input space that produces a more extreme result than any finite set of inputs. Do these have a useful meaning in terms of describing that function?

One preferred stimulus already contains some information about the tuning function. Ponce et al., 2019 showed that the evolved stimulus can be used as a simple model to predict a neuron's responses to natural images: A natural image that is more similar to the super-stimuli will elicit a higher firing rate than a more dissimilar image.

Of course, we do not think this is a sufficient description of the tuning function. As noted in the previous response, XDream not only maps one extreme on the 1D tuning function of a neuron, but also a cross-section through it, since the trajectory of each evolution contains stimuli that elicit a wide range of responses. Both the extreme and the trajectory should be useful for generating hypotheses and guiding investigation of the tuning function.

3) The authors showed that the generative model in XDream was "expressive", but those demonstrations also show what the model does not capture, namely high-frequency content of images (fine details are lost in all the examples in (what should be) Figure 3). Is this a limitation for the methodology? It seems so, since the model shouldn't then be able to capture optimal stimuli for neurons that respond to fine details. I suspect this is not a severe practical

limitation, but it is there.

Thank you for raising this point. We have pondered the question of generator bias and refined our thinking about it several times. The generative neural networks are almost certainly limited in what images they can generate, but we find it non-trivial to describe just what that limit is. Unfortunately, we do not have an analytical way to describe the extent of the stimulus space encompassed by the generators.

We do not think that the bias is about fine details *per se*. The new S6 Fig gives an example of recreating fine details of feature preference (gratings of about 2 pixels in spatial frequency, for AlexNet conv1 units). To take another example, in (what is now) S3 Fig, not *all* details of the target images were recovered, but we don't think what was lost was necessarily the *fine* details. For the example of Pluto and Charon, in the reconstruction (the last row, or leftmost column when viewed in portrait) the entire moon is missing, but much detail on Pluto itself about the colors (if not their exact spatial arrangement) is preserved. Or, consider the reconstruction of the painting of a wheatfield. The reconstruction is much higher contrast than the original, but it still seems to preserve the spirit of van Gogh's signature brushstrokes. In general, in this figure, we found that many of the inaccuracies are consistent with the limit of the loss function–blurrier images and loss of sharp edges when using pixel-level loss, and large non-localized changes that nonetheless still leaves the image "looking similar" when using pool5 loss. Because the lost features depend specifically on the loss function, it is difficult to attribute the losses to the generator (lines S170–174).

Based on the training objective of these generative networks—that is, to make images that look like ImageNet images (mostly photographs)—we may assume that the generators probably cannot make arbitrary pixel combinations that deviate far from natural image statistics, such as random Gaussian-noise images. However, since neurons may be biased to representing natural images too, and since that is the functionally-relevant domain interesting for study anyway, we agree with the reviewer that this should not pose a severe practical limitation.

The next reply may also be relevant, as it discusses whether the biases or limitations of the generators are specifically constrained by ImageNet, or whether they are more general biases that perhaps encompass a wide domain of natural images. This and the next reply are summarized and added to the main text (lines S335-344).

4) The authors should acknowledge the bias that is built into their methodology by looking at samples from fixed image databases. ImageNet does have a particular structure and, unless I'm mistaken, all the tested generators were trained on this structure (implicitly by inverting AlexNet). This will bias towards finding features similar to those necessary for describing ImageNet in particular. This is not just an issue of whether the generative model can reproduce an image when forced to (e.g. the Figure that should be Figure 3) but whether it will tend to promote certain features over others. ImageNet will provide an implicit bias.

We completely agree with the reviewer that the generative networks have an implicit bias partly provided by ImageNet. We would in fact argue that, to be useful for an efficient search, a generator will *need* an implicit bias (whether it is in what images are represented, or in how images are arranged relative to each other), since it is inefficient to search in the raw pixel representation space containing all possible images (of a given resolution; S4 Fig).

While certain features may be promoted over others, it is a separate question whether or how this adversely affects the search for optimal stimuli. We tested PlacesCNN (AlexNet architecture trained on the Places-205 dataset instead of ImageNet) in Fig 2, specifically to assess whether the ImageNet-trained generators will still be general-enough to create super stimuli for a classifier trained on another dataset. Indeed, similar relative activation values were achieved in both AlexNets regardless of their training set. For a more direct comparison, we have additionally trained the DeePSiM-fc6 generator on the Places-365 dataset (a later version of Places-205), and tested both generators (identical architecture, different training sets) respectively on both classifiers (identical architecture, the same two different training sets). The results are shown in what is now S4 Fig, panel b. By and large, both generators performed equally well on both classifiers, supporting the notion that the generators have learned general features (at least, general enough to apply across these two datasets). Furthermore, in Nguyen et al., 2016 (https://arxiv.org/abs/1605.09304), the same generator was successfully used to generate preferred stimuli for an even wider range of target networks, including an action-recognition network and an AlexNet with RGB channels permuted (which would make it prefer otherwise normal images with highly unnatural colors, such as wine glasses with green wine).

Finally, to the extent that biological neurons have a large "invariance manifold," yet another question is whether, while being able to create super stimuli, XDream is still biased in over-representing a subset of that manifold congenial to the generator. For example, for a face neuron that responds equally well to a photograph and a cartoon, the generators may tend to, or perhaps even exclusively, produce a photographic optimal stimulus. Is this a problem? The answer seems to depend on what the goal is and what the alternative is. Since animals live in a natural world, a bias on natural images seem a reasonable, ethologically-relevant prior, and for this purpose ImageNet is among the best available datasets. Indeed, it seems harder to motivate any other prior, including a uniform prior on all possible pixel combinations. However, it is worth pointing out that, should a different prior be needed, XDream can be outfitted with another generator that reflect the chosen prior. (Training such a generator may be challenging, but an entire, active field of generative modeling is dedicated to such problems.)

In sum, we agree that the generators are biased to "natural image statistics" as represented in the dataset in a hard-to-define way, although available data suggest it does not adversely affect search for preferred images.

5) The claim about robustness to noise is overstated. Figure 6 makes the technique look quite susceptible to Poisson noise, depending upon target layer.

Thank you for pointing this out. We originally intended to compare the noise robustness of different optimization algorithms; the header is overly broad and we have changed it. Although the genetic algorithm is more robust than the other two optimization algorithms, it still sensitive to noise to an extent. To characterize the noise sensitivity better, we have collected additional data where we varied the amount of noise. In the new Fig 4b, the optimization results are presented according to amount of stochasticity quantified in intuitive and commonly-used terms (self-correlation, noise as fraction of signal, and signal-to-noise ratio). This can function as a reference table that, based on the empirical amount of noise, helps an investigator better estimate how well XDream can perform.

Minor issues:

1) Figure 1b and 1c: there are 9 minor tick marks and ten example images.

Thank you for catching this. The minor tick at 0 was blocked by a major tick mark. It has been fixed.

2) Figure 2 and 3 appear to be swapped (at least in the pdf I received).

Thank you. It was a mistake when uploading the figures and should now be fixed.

3) Figure 2: label the random ImageNet samples in the Figure (grey boxes). label the dotted line as "maximum ImageNet response" or something similar. This will make the figure easier to read and digest.

4) line 112: ...we qualitative[ly] assessed...

5) lines 136-7: delete either "it can generate" or "can be generated"

6) Figure 4: label the open and solid violin plots on the Figure

3–6) Thank you for the suggestions. We have applied all of them.

7) Table 1: the caption appears to be a Latinate placeholder.

8) lines 211-9: this section needs to be rewritten. There needs to be a reference to Table 1, otherwise "slope" is introduced before the reader has seen such a thing. It isn't in a figure or preceding discussion. One is left to infer what variables the linear regression is being performed upon.

7–8) We have inadvertently placed the table caption in the main text. This is now fixed, and "slope" is also more clearly defined in the table legend.

Reviewer 2: I will upload the pdf with my comments in it, where I have found typos and made wording suggestions.

We have fixed all the typos identified and incorporated the wording suggestions, thank you. We added the Zeiler & Fergus reference. As requested, we added a supplementary figure (new S2 Fig) showing evolved images for intermediate layers for non-AlexNet architectures. There are a few comments in the PDF document for which a written response would be useful:

Regarding the gradient of slopes in Table 1, we added to the main text offering our interpretation (lines 263–266).

A set of recommended hyperparameters had been included in Supplementary Tables S1 and S2.

Regarding the "rather high" mutation rate in Table S1, we note that: 1) mutation rate and size work in conjunction, 2) mutation size is not scale-free but rather depends on the scale of the input space of the generator, which

needs to be measured empirically based on the input training data. We did so for one example generator, DeePSiM-fc6; the input codes during training have standard deviation of approx. 15—in this light, a mutation size of 0.5 is relatively small. In general, rather than manually choosing hyperparameters, we empirically optimize them. An example of how hyperparameter choice matters for XDream performance is shown in what is now S5 Fig.

We switch next to the questions and suggestions that were separate from those in the PDF document (for which, again, we are grateful).

Review of XDream: finding preferred stimuli for visual neurons using generative networks and gradient-free optimization

by Gary Cottrell

This paper uses an existing method for finding optimal visual stimuli for neurons that has previously been applied in Macaque in a paper in Cell in 2019. The goal here is to elucidate how robust the method is by applying it to several deep network models with varying architectures and at different layers of the networks. Using network models allows for extensive experimentation that is impractical in biological preparations. The model proves to be very robust to various parameter regimes, and finds stimuli that drive the neuron much more than any of the over 1 million images in the ImageNet dataset. One of the most interesting findings here is that, using different image generators or different initial conditions, the model finds multiple images that drive the neurons similarly, and these images resemble one another to the human eye. Hence they suggest that there is an optimal image manifold in the latent space of images. Unfortunately, this point was already made in the prior paper with actual monkey visual neurons.

Since the authors postulate that there is an invariance manifold, it would be useful to test this idea by looking at what is generated by a linear interpolation of the codes found from different initial conditions, and how well those interpolated images drive the model neuron. While it is unlikely that the manifold is linear, since the images are similar, they are probably nearby in this space.

We would like to point out that there are many nearby images that can drive the target neurons approximately equally well: During the last generations, there tend to be subtle changes to the image and small changes in the activation values.

Of course, this does not imply the converse, that similarly-activating images are all nearby. To test this, we performed the suggested analysis (thank you for this wonderful idea). We measured model unit responses to linear interpolations between codes optimized from different initial conditions. (Of note, we interpolated in code space and not pixel space. We think this is more reasonable because images quite different on a pixel level can elicit similar responses, e.g., Fig 3c.) The results are added in the new Fig 3b. The manifolds do not appear *linear*, since extrapolating outward from the line segment connecting two strong stimuli results in significant loss in stimuli effectiveness. For early layer (conv2 and conv4) units, extrapolating inward resulted in higher activation than extrapolating outward, showing a tendency for the manifolds to be *convex*. But the convexity is not perfect, since the interpolations are still weaker than the

endpoints (original optimized images). For fc6 and fc8 units, the interpolation midpoint has much weaker activity than either endpoint, suggesting either that the set of strong stimuli is indeed disjoint, or that the invariance manifold has complex, non-convex geometry (meaning that activity-preserving interpolations between two optimal images are *nonlinear*).

To elaborate, we should first specify that our notion of invariance manifold is intended to mean the collection of codes/images that activate a neuron similarly; an equivalent term is a "level set." A level set exists by definition (even if it may only contain one point), but it may be linear (i.e., it is a linear subspace of the image representation space), convex (i.e., points on an interpolation between any two members of the set are also members of the set), connected (i.e., one can travel between any two points in the set while always remaining in the set), or none of these. The geometry of the level set is dependent on both the image representation space (the "code" space) and the target neuron. To take a concrete example, consider so-called "face neurons" in the middle lateral (ML) face patch, and consider a space of faces images defined by "face shape axes" (Chang et al., 2017). The neurons have linear level sets ("invariance manifolds") reflected by their linear tuning function. On the other hand, if we consider the same neurons with regards to pixel-level representation, one can imagine that the "invariance manifolds" will be highly nonlinear.

In a sense, to understand the tuning of a neuron is equivalent to identifying the image representation space in which the neuron has a simple (e.g., linear) "invariance manifold." The fact that we do not find linear or convex manifolds for later layer neurons suggests that, even though the generative networks enables us to find effective stimuli, these networks still do not perfectly match the feature representation of the neurons.

We thank the reviewer for raising this interesting point and summarize the discussion in the main text (lines S345–361).;

In the paragraph on other things one could study with this approach, such as correlated firing, synchronized firing, LFPs, etc., it would be helpful to say what you would optimize in a couple of cases. E.g., you might say, "for example, we could optimize based on increased correlations in firing rates between neurons" or some such.

Thank you. We have followed your suggestion.

The paper could be improved by moving more of the information into the methods section or into supplementary material. There are many details that make the exposition rather tough sledding for the reader. In particular, the section on the effects of different generators has a couple of caveats, e.g., "except for CaffeNet conv2", and "The pixel-based image generator, compared to generative neural networks, worked more poorly in all target layers other than CaffeNet conv2 except when compared to deepsim-norm2 ($p = 1$ compared to deepsim-norm2; $p > 0.14$ compared to other generators in CaffeNet conv2; $p < 10^{-4}$ in all other comparisons; FDR-corrected for 32 tests comparing each generator to raw-pixel in each target layer)." This amount of detail makes my eyes glaze over. I think this section doesn't add a lot to the paper, and could profitably be moved to the supplementary material.

Thank you. To make this section flow better, we have re-written to report the statistics in a clearer fashion. We think this content does add to the results; please see the next reply.

Similarly, the section on different optimizers doesn't flow well. The GA works better on some layers and the other two algorithms work better in some other layers. I'm not sure I care, and I'm not sure what the take-home message is. Again, there are similar "this works better in this layer and that works better in other layers" results in the noise experiments. I think the noise experiments are important, as this is a more realistic case. Unfortunately, the results in Figure 6 are not encouraging. I would disagree with the header for this section: "XDream is robust to noise in neuronal responses." It doesn't seem that robust. I wonder if there is a fix for this - for example, can you average the rate over some time interval of the Poisson process instead of simply sampling from it? Unfortunately, I don't know enough about Poisson processes to know if this is a good suggestion or not.

Thank you for these comments. In terms of practical utility, we agree that the sections on generator and optimizer choice are of limited use, in large part because it turns out that the original choice of generator and optimizer happens to be near-optimal, among the options tested. However, in terms of developing the algorithm, we think these sections are useful for highlighting the modularity of the algorithm, suggesting directions in which it can be improved, and explaining extensions we have tested.

We agree that the original section header is inaccurate (the section was originally meant to compare among optimization algorithms.) and have changed it to "The influence of neuronal noise on XDream." Now, we better characterize the algorithm's ability to tolerate varying amounts of noise; the results are presented in the new Fig 4b.

We consistently use a Poisson noise model, which has a Fano factor (variance-to-mean ratio) of 1, and which has been a typical way to characterize spiking activity in cortex (Gabbiani, 2010, *Mathematics for Neuroscientists*). As discussed in Methods, in this model, the relative amount of noise (standard deviation-to-mean ratio, which is closely related to signal-to-noise ratio) will decrease with more spikes observed—this could result from a higher firing rate, longer observation time, and/or several repetitions, as the reviewer suggested. To account for these factors, we varied the "expected max number of spikes"—that is, we normalized all responses by the expected max activation in 2,500 natural images, scaled the responses by a varying number, then used that as the rate of a Poisson process (Methods). This procedure results in different levels of noise. We further quantify the amount of noise with several commonly used measures: trial-to-trial correlation, standard deviation-to-mean ratio, and signal-to-noise ratio. The results can be used like a look-up-table to map experimentally observed neuronal noisiness to how XDream is expected to perform. In (what is now) Fig 4a, the "Noisy" condition corresponds to expecting 20 spikes from the best image—for example, a firing rate of 100 spks/s and a 200 ms observation window. The spike number can be higher (and effective noise lower) when using a longer observation window, multi-unit activity, and/or more image repetitions.

The number of repetitions is considered separately. Since we impose a strict limit of 10,000 image *presentations*, more repetition means fewer unique images shown and consequently fewer optimization iterations. This trade-off has been considered during parameter optimization (now S5 Fig, last panel); for the genetic algorithm, the final performance decreases when the number of repetitions is greater than 1, at least with the original noise level (20 spikes expected in response to a preferred image). It is possible that with even noisier data, more repetitions could be beneficial, although this would make optimization harder in general.

One concern is that, although the authors state that "we focus on the more biologically relevant scenario where there is no information about the architecture and weights of the target model, and where we only have access to a few, potentially stochastic, activation values from the neurons." In fact, they don't have access to only a few activations. The model is used to generate 10,000 images to find the optimal one. This seems biologically unrealistic. I skimmed the previous paper, and there they used many fewer generations for the monkeys - 200. Since this is what is apparently possible in biological preparations, it seems like they should evaluate how much is gained in 200 generations and compare that to the 500 generations they used here. This would provide a better estimate of what is possible today.

The main limitation in biological recordings is time, which maps directly to the number of "queries" (image presentations). The number of queries is therefore an important concern for us, motivating our strict limit of 10,000 image presentations throughout the paper. This number is quite realistic. For example, in Ponce et al., 2019, each generation consisted of 40 synthetic and 40 reference images, which for 200 generations corresponds to $40 \times 40 \times 200 = 16,000$ image presentations (of which 8,000 were used for evolution). What's more, in this paper the population size (images per generation) is 20 rather than 40, making it unfair to directly compare the number of generations.

Still, 10,000 is just an arbitrary number. To gauge how much optimization can be achieved over time, take Fig 1b as an example: Activation grows roughly logarithmically with generation, and most of the optimization gain is achieved in the first 250 generations (activation of around 60 by generation 250 *vs.* around 70 by 500).

Originality

The originality is tempered somewhat by the fact that this is investigating an existing method that has already been used in Macaque visual cortex. Obviously, this is different in the sense that using convnets as the preparation allows for much more extensive experimentation with the method than would be possible with a biological one, which is the point of this paper. For example, they can test the neuron's response to the over one million images in ImageNet, and then compare the results of this brute force search to the effectiveness of XDream. They vary just about everything and still find that the method works well. So it is original in that sense, but I have to think that the big-font headline is the first paper.

We are glad that the reviewer agrees with our motivation for the paper.

Several neurophysiologists are now interested in this type of methods to study neural responses. We hope that the current paper will justify parameter choice and better describe performance under conditions that cannot quite be tested experimentally for practical reasons, as the reviewer points out.

Innovation

Again, the innovation here is to benchmark their method using in silico models. This kind of thing has been done before with other analyses of deep net features.

We are not entirely sure which "other analyses"the reviewer is referring to. Perhaps the reviewer is referring to DeepDream and similar studies where investigators visualize unit preferences. However, we emphasize that DeepDream and similar algorithms strictly depend on completely knowledge of the system's weights and architecture. There have also been studies that apply neurophysiological methods to explain deep nets (Pospisil et al., 2018, eLife); the chief goal in those studies is not to use deep nets to benchmark/validate a method. As the reviewer points out, our main goal here is to connect with the Neuroscience literature and help experimentalists who want to study neural coding for visual features in cortex.

High importance to researchers in the field:

To the extent to which other researchers may start to use this method, the importance here is that neuroscientists can get some assurance that the method is robust, and that they needn't worry too much about optimizing the meta-parameters.

Indeed, we agree with the reviewer.

"Similar activation values notwithstanding, the optimized images were different on a pixel level (Fig 4b); they may comprise an "invariance manifold" for each neuron that contains similar but not identical images eliciting comparable activation values (see Discussion)." I think this is an important point, so it would be good to test the hypothesis by looking along a line between some of the image codes to see if there are optimal images between these. This would be of interest to researchers using this method.

Thank you for this suggestion. As detailed above, we have tested this idea and reported the results.

Significant biological and/or methodological insight:

There is not much in the way of biological insight here, but the paper does demonstrate that this methodology is robust.

Indeed, the focus here is on the method itself. On a speculative note, several works have argued that the ConvNets that we test here constitute an approximation to visual cortex (e.g., Yamins & Dicarlo 2014 among many others). To the extent that visual cortex can be modeled by a ConvNets (a matter of significant debate in the field), the conclusions drawn here could extrapolate to visual cortex. However, the reviewer correctly points out that the main focus here is on the method.

Rigorous methodology

This is quite a rigorous test of the model.

Substantial evidence for its conclusions

The evidence that this approach works well over a variety of convnet architectures and layers is extensive. On the other hand, I think the evidence for robustness to noise is weak. This is a point the authors should address in a revision of the paper.

We have revised this part as suggested (see above).

Minor comments:

I'm not quite sure what this sentence means: "the standard deviation was lower than 10% of the activation values (Fig 4a)."

This means that if a different *random* initialization is used, on average, one would expect no more than a 10% change in the activation value. We have made the relevant figure and description clearer.

I'm not sure the slopes in Table 1 give a very intuitive idea of how much improvement you get by using good vs. bad initializations. A bar chart might be better. Also, while the text suggests that there isn't much difference, the slopes seem fairly big as you go deeper in the net using the ivt method. I don't know if that conclusion of mine is warranted. I.e., I would better understand this if the actual differences in medians were shown instead of the slope. Perhaps another violin plot would work here? It seems slightly counterintuitive to conclude that there is only slight variation in the optimized image activation while the p-values for the differences are on the order of 10^{-77}.

We apologize that the legend for this table was misplaced into the main text, making the table hard to interpret. This has now been fixed. Regarding the suggesting of a bar chart/violin plot, the effect of using different initializations are indeed reported, as a violin plot, in (what is now) Fig 3d.

The slope was intended as a more specific statistical test (because it specifies the direction of change) than ANOVA. We report p-values for the slope (Table 1) and ANOVA (main text). To be sure, there is an effect of using a *good* initialization—provided the investigator knows, *a priori*, a preferred image comparable to the best in approx. 1 M images (a strong assumption). Nevertheless, this is consistent with the conclusion that there is no effect when using different *random* initializations and, indeed, when using middle or worst ImageNet images. These issues are discussed in the last two paragraphs in the main text, section "XDream is robust to different initial conditions."
