# Supplementary material for: XDream: Finding preferred stimuli for visual neurons using generative networks and gradient-free optimization
Source: PLoS Comput Biol. 2020 Jun 15;16(6):e1007973. doi: 10.1371/journal.pcbi.1007973 (PMC7316361; doi:10.1371/journal.pcbi.1007973)
Supplement: Attachment — Submitted filename: PCOMPBIOL-D-19-01642_R1_reviewer_gwc_comments.pdf [file pcbi.1007973.s014.pdf]

PLOS Computational Biology

XDream: finding preferred stimuli for visual neurons using generative networks and gradient-free optimization

--Manuscript Draft--

**Manuscript Number:**	PCOMPBIOL-D-19-01642R1
**Full Title:**	XDream: finding preferred stimuli for visual neurons using generative networks and gradient-free optimization
**Short Title:**	XDream: Finding preferred visual stimuli via optimization
**Article Type:**	Research Article
**Keywords:**	computational neuroscience; visual processing; neuronal coding; ventral visual stream; preferred stimuli; deep generative neural networks; genetic algorithm; convolutional neural networks
**Abstract:**	A longstanding question in sensory neuroscience is what types of stimuli drive neurons to fire. The characterization of effective stimuli has traditionally been based on a combination of intuition, insights from previous studies, and luck. A new method termed XDream (EXtending DeepDream with real-time evolution for activation maximization) combined a generative neural network and a genetic algorithm in a closed loop to create strong stimuli for neurons in the macaque visual cortex. Here we extensively and systematically evaluate the performance of XDream. We use ConvNet units as in silico models of neurons, enabling experiments that would be prohibitive with biological neurons. We evaluated how the method compares to brute-force search, and how well the method generalizes to different neurons and processing stages. We also explored design and parameter choices. XDream can efficiently find preferred features for visual units without any prior knowledge about those units. XDream extrapolates to different layers, architectures, and training datasets, performing better than brute-force search, and often better than exhaustive sampling of > 1 million images. Furthermore, XDream is robust to choices of multiple image generators, optimization algorithms, and hyperparameters, suggesting that its performance is locally near-optimal. Lastly, we found no significant advantage to problem-specific parameter tuning. These results establish expectations and provide practical recommendations for using XDream to investigate neural coding in biological preparations. Overall, XDream is an efficient, general, and robust algorithm for uncovering neuronal tuning preferences using a vast and diverse stimulus space. XDream is implemented in Python, released under the MIT License, and works on Linux, Windows, and MacOS.
**Additional Information:**	
**Question**	**Response**
**Financial Disclosure**<br><br>Enter a financial disclosure statement that describes the sources of funding for the work included in this submission. Review the submission guidelines for detailed requirements. View published research articles from *PLOS Computational Biology* for specific examples.<br><br>This statement is required for submission and **will appear in the published article** if the submission is accepted. Please make sure it is accurate.	W.X. and G.K. are supported by the Center for Brains, Minds and Machines funded by NSF528STC award CCF-1231216 and also by NIH R01EY026025. The funders had no role in study design, data collection and analysis, decision to publish, or preparation of the manuscript.<br>https://www.nsf.gov<br>https://www.nih.gov

Powered by Editorial Manager® and ProduXion Manager® from Aries Systems Corporation

**Unfunded studies**<br>Enter: *The author(s) received no specific funding for this work.*<br><br>**Funded studies**<br>Enter a statement with the following details:<br>• Initials of the authors who received each award<br>• Grant numbers awarded to each author<br>• The full name of each funder<br>• URL of each funder website<br>• Did the sponsors or funders play any role in the study design, data collection and analysis, decision to publish, or preparation of the manuscript?<br>• **NO** - Include this sentence at the end of your statement: *The funders had no role in study design, data collection and analysis, decision to publish, or preparation of the manuscript.*<br>• **YES** - Specify the role(s) played.<br><br>* typeset	
**Competing Interests**<br><br>Use the instructions below to enter a competing interest statement for this submission. On behalf of all authors, disclose any competing interests that could be perceived to bias this work—acknowledging all financial support and any other relevant financial or non-financial competing interests.<br><br>This statement **will appear in the published article** if the submission is accepted. Please make sure it is accurate. View published research articles from *PLOS Computational Biology* for specific examples.	The authors have declared that no competing interests exist.

Powered by Editorial Manager® and ProduXion Manager® from Aries Systems Corporation

**NO authors have competing interests**<br><br>Enter: *The authors have declared that no competing interests exist.*<br><br>**Authors with competing interests**<br><br>Enter competing interest details beginning with this statement:<br><br>*I have read the journal's policy and the authors of this manuscript have the following competing interests: [insert competing interests here]*<br><br>* typeset	
**Data Availability**<br><br>Authors are required to make all data underlying the findings described fully available, without restriction, and from the time of publication. PLOS allows rare exceptions to address legal and ethical concerns. See the PLOS Data Policy and FAQ for detailed information.<br><br>A Data Availability Statement describing where the data can be found is required at submission. Your answers to this question constitute the Data Availability Statement and **will be published in the article**, if accepted.<br><br>**Important:** Stating 'data available on request from the author' is not sufficient. If your data are only available upon request, select 'No' for the first question and explain your exceptional situation in the text box.<br><br>Do the authors confirm that all data underlying the findings described in their manuscript are fully available without restriction?	Yes - all data are fully available without restriction
**Describe where the data may be found in full sentences. If you are copying our sample text, replace any instances of XXX with the appropriate details.**	All data underlying the findings described in their manuscript have been made available in the publicly available GitHub repository at https://github.com/willwx/XDream

• If the data are **held or will be held in a public repository**, include URLs, accession numbers or DOIs. If this information will only be available after acceptance, indicate this by ticking the box below. For example: *All XXX files are available from the XXX database (accession number(s) XXX, XXX.).*<br>• If the data are all contained **within the manuscript and/or Supporting Information files**, enter the following: *All relevant data are within the manuscript and its Supporting Information files.*<br>• If neither of these applies but you are able to provide **details of access elsewhere**, with or without limitations, please do so. For example:<br><br>*Data cannot be shared publicly because of [XXX]. Data are available from the XXX Institutional Data Access / Ethics Committee (contact via XXX) for researchers who meet the criteria for access to confidential data.*<br><br>*The data underlying the results presented in the study are available from (include the name of the third party and contact information or URL).*<br>• This text is appropriate if the data are owned by a third party and authors do not have permission to share the data.<br><br>* typeset	
Additional data availability information:	

We are thankful to the reviewers for their insightful and constructive comments. We have addressed the comments point-by-point and incorporated all replies into the text.

Due to the editor's request to reformat the article to conform to the Software Article format of the journal, substantial parts of the manuscript have been moved to the supplementary material; we apologize in advance for the major changes. In the following replies, line references correspond to the clean version (no tracked changes) of the revised manuscript (line #) or supplementary material (line S#).

Reviewer 1: The authors produce an analysis of the XDream method that uses generative networks with a genetic algorithm for obtaining stimuli that will optimally drive neurons. They test this method by using it on pre-trained deep neural networks. They show that XDream can consistently find stimuli that will drive units in pre-trained networks to responses greater than those of the image set from which the networks were trained. This result is approximately independent of depth in the network and is independent of initial conditions. It is relatively robust to the choice of generative network, as long as that generator produces a "high-level" representation. These results are somewhat robust to the choice of optimization but are impacted by adding noise to the representation.

This looks at an important issue for visual neuroscience, that of finding stimuli that drive neurons in a manner that is not plagued by various experimental and theoretical biases (cf. Olshausen-Field "What is the other 85% of V1 doing?" and Carandini, et al 2005, "Do we know what the early visual system does?"). The XDream tool is an example of an emerging methodology for dealing with this problem and studying its capabilities and drawbacks is therefore important and useful to the field. The paper is clear. I recommend publication after the authors have addressed the feedback below.

Thank you for mentioning Olshausen & Field, 2005 and Carandini et al., 2005. We certainly embrace their viewpoint and now cite them in the Discussion (line S338).

1) The most important issue the authors should address is to provide some acknowledgement or discussion of the notion of optimal or "preferred" stimuli in the first place. By their methodology, they are looking for a single image that will optimally drive neurons, but this is probably not the right way to think about the coding problem, certainly not for neurons and probably not for the units in deep networks as well. The "single best image" approach is going to gloss over issues like contextual dependence. These optimal stimuli may in fact be a very small set that drives responses because they combine some "feature" for which the neuron/unit codes with exactly the right context to enhance the response. Depending on the size of this space and the nature of the context, one might wind up with an image that is not very informative of the actual coding properties of the neuron or unit. The authors use the phrase "true feature preference" in the introduction, but need to acknowledge that their method may not find it either. Furthermore, this problem is compounded in the case of real neurons, in which there are feedback and lateral inputs that

produce extra-classical effects. These effects may or may not appear in the models that the authors are testing, since they are all feedforward networks. This set of questions will almost certainly lead to the "invariance manifold" that the authors discussed.

None of this takes away from the authors' work, but users of the method should be aware of these issues.

Thank you for this insightful evaluation of the XDream method. The reviewer makes many interesting points here, which we will discuss one by one.

In terms of finding the "true feature preference," we acknowledge here and in text that we cannot guarantee that XDream will converge to the global optimum ("Of note, these results do not [...] provide a proof of convergence to the global maximum for all visual neurons" (lines S177–179). Data such as (what is now) S3 Fig suggest that XDream can get quite close in certain cases, whereas data such as (what is now) Fig 3d, comparing random init and best-ivt init for layer fc6 and fc8, suggest that in certain cases XDream may not find the global optimum within the amount of observation allowed (10,000 image presentations).

Regarding the "right way to think about the coding problem," we also concur that the optimal stimulus of a neuron only gives us a limited understanding of the function of that neuron in representing visual information. We focused on defining the optimal stimulus, which we believe can serve as the first step to defining stimulus parameters whose variation is encoded by that neuron (e.g., Pauspathy & Connor, 2002). This is in turn essential for understanding how a population of neurons, with such a collection of "tuning curves," can lead to the representation of complete visual percepts (e.g., Chang & Tsao, 2017). As the discovery of visual tuning features is often serendipitous, we think XDream is useful in providing ideas of what visual properties investigators should test, both in areas whose tuning is still poorly understood, and in well-studied areas where studies may nevertheless have missed important aspects of tuning. Furthermore, we note that during the optimization process, XDream does test thousands of related images, covering the target unit's response levels both widely and densely (Fig 1c). Closer analyses of these images will likely reveal richer information about the tuning curve (e.g., properties of tuning and invariant properties) than what's reflected by the single best image.

In terms of contextual dependence, we again completely agree with the reviewer that XDream is designed to identify any and all aspects of the stimulus that will activate the neuron, including features that may be considered "context." However, we think that the notion of context is somewhat arbitrary (in the same way that there is no sharp distinction between classical and extra-classical receptive fields). What does it mean for a neuron to distinguish between "object" and "context"? Perhaps, different stimulus features interact in a non-linear fashion, in a possibly hierarchical manner (e.g., a face neuron may have a preferred face orientation, but it will not respond to other objects in that orientation); XDream will not automatically factorize the different features, a fact that can be considered a shortcoming of the method. Still, in such a case, we believe that both features are part of the "actual coding properties" of the

neuron, and it is valuable for XDream to uncover them.

Finally, concerning recurrent (feedback and lateral) inputs that may produce extra-classical effects in real neurons, the reviewer correctly points out that they are absent from the models we have tested in the paper. Therefore, we constructed a toy model that mimics the iconic extra-classical effect of "surround suppression" (Allman et al., 1985, Annu Rev Neurosci): We took a single feature channel from the conv1 layer of AlexNet and tiled it spatially with a central, circular excitatory region and a concentric suppressive ring, creating a unit that is activated by a feature in the center but suppressed by the same feature in the surround (see the new S6 Fig). When evolving a stimulus for this unit, XDream indeed creates a center-ON surround-OFF pattern that reflects the pattern preference and matches varying receptive field sizes. In general, we think that as long as any extra-classical effect is reflected in the part of the firing rate of a neuron used for the fitness function (in particular, the relevant response time window needs to be included), and to the extent that XDream is a general method for finding stimuli that maximizes stimulus response—which appears true to the extent we have tested—XDream should be able to address the issue of recurrent, extra-classical effects. In fact, we think it is an interesting future direction to study the extra-classical tuning of ventral stream neurons by using XDream with different-sized stimuli or different response time windows.

Thank you for these questions that clarify the utility of XDream. We have added to the text summarizing all the discussion above (lines 381–396, S365–372).

2) Related to point 1, the authors should comment on the possible ethological relevance of super-stimuli. It is not surprising that such stimuli exist. Given that each unit produces a 1D parameterized function of image space, one should be able to find a point in the input space that produces a more extreme result than any finite set of inputs. Do these have a useful meaning in terms of describing that function?

One preferred stimulus already contains some information about the tuning function. Ponce et al., 2019 showed that the evolved stimulus can be used as a simple model to predict a neuron's responses to natural images: A natural image that is more similar to the super-stimuli will elicit a higher firing rate than a more dissimilar image.

Of course, we do not think this is a sufficient description of the tuning function. As noted in the previous response, XDream not only maps one extreme on the 1D tuning function of a neuron, but also a cross-section through it, since the trajectory of each evolution contains stimuli that elicit a wide range of responses. Both the extreme and the trajectory should be useful for generating hypotheses and guiding investigation of the tuning function.

3) The authors showed that the generative model in XDream was "expressive", but those demonstrations also show what the model does not capture, namely high-frequency content of images (fine details are lost in all the examples in (what should be) Figure 3). Is this a limitation for the methodology? It seems so, since the model shouldn't then be able to capture optimal stimuli for neurons that respond to fine details. I suspect this is not a severe practical

limitation, but it is there.

Thank you for raising this point. We have pondered the question of generator bias and refined our thinking about it several times. The generative neural networks are almost certainly limited in what images they can generate, but we find it non-trivial to describe just what that limit is. Unfortunately, we do not have an analytical way to describe the extent of the stimulus space encompassed by the generators.

We do not think that the bias is about fine details *per se*. The new S6 Fig gives an example of recreating fine details of feature preference (gratings of about 2 pixels in spatial frequency, for AlexNet conv1 units). To take another example, in (what is now) S3 Fig, not *all* details of the target images were recovered, but we don't think what was lost was necessarily the *fine* details. For the example of Pluto and Charon, in the reconstruction (the last row, or leftmost column when viewed in portrait) the entire moon is missing, but much detail on Pluto itself about the colors (if not their exact spatial arrangement) is preserved. Or, consider the reconstruction of the painting of a wheatfield. The reconstruction is much higher contrast than the original, but it still seems to preserve the spirit of van Gogh's signature brushstrokes. In general, in this figure, we found that many of the inaccuracies are consistent with the limit of the loss function–blurrier images and loss of sharp edges when using pixel-level loss, and large non-localized changes that nonetheless still leaves the image "looking similar" when using pool5 loss. Because the lost features depend specifically on the loss function, it is difficult to attribute the losses to the generator (lines S170–174).

Based on the training objective of these generative networks—that is, to make images that look like ImageNet images (mostly photographs)—we may assume that the generators probably cannot make arbitrary pixel combinations that deviate far from natural image statistics, such as random Gaussian-noise images. However, since neurons may be biased to representing natural images too, and since that is the functionally-relevant domain interesting for study anyway, we agree with the reviewer that this should not pose a severe practical limitation.

The next reply may also be relevant, as it discusses whether the biases or limitations of the generators are specifically constrained by ImageNet, or whether they are more general biases that perhaps encompass a wide domain of natural images. This and the next reply are summarized and added to the main text (lines S335-344).

4) The authors should acknowledge the bias that is built into their methodology by looking at samples from fixed image databases. ImageNet does have a particular structure and, unless I'm mistaken, all the tested generators were trained on this structure (implicitly by inverting AlexNet). This will bias towards finding features similar to those necessary for describing ImageNet in particular. This is not just an issue of whether the generative model can reproduce an image when forced to (e.g. the Figure that should be Figure 3) but whether it will tend to promote certain features over others. ImageNet will provide an implicit bias.

We completely agree with the reviewer that the generative networks have an implicit bias partly provided by ImageNet. We would in fact argue that, to be useful for an efficient search, a generator will *need* an implicit bias (whether it is in what images are represented, or in how images are arranged relative to each other), since it is inefficient to search in the raw pixel representation space containing all possible images (of a given resolution; S4 Fig).

While certain features may be promoted over others, it is a separate question whether or how this adversely affects the search for optimal stimuli. We tested PlacesCNN (AlexNet architecture trained on the Places-205 dataset instead of ImageNet) in Fig 2, specifically to assess whether the ImageNet-trained generators will still be general-enough to create super stimuli for a classifier trained on another dataset. Indeed, similar relative activation values were achieved in both AlexNets regardless of their training set. For a more direct comparison, we have additionally trained the DeePSiM-fc6 generator on the Places-365 dataset (a later version of Places-205), and tested both generators (identical architecture, different training sets) respectively on both classifiers (identical architecture, the same two different training sets). The results are shown in what is now S4 Fig, panel b. By and large, both generators performed equally well on both classifiers, supporting the notion that the generators have learned general features (at least, general enough to apply across these two datasets). Furthermore, in Nguyen et al., 2016 (https://arxiv.org/abs/1605.09304), the same generator was successfully used to generate preferred stimuli for an even wider range of target networks, including an action-recognition network and an AlexNet with RGB channels permuted (which would make it prefer otherwise normal images with highly unnatural colors, such as wine glasses with green wine).

Finally, to the extent that biological neurons have a large "invariance manifold," yet another question is whether, while being able to create super stimuli, XDream is still biased in over-representing a subset of that manifold congenial to the generator. For example, for a face neuron that responds equally well to a photograph and a cartoon, the generators may tend to, or perhaps even exclusively, produce a photographic optimal stimulus. Is this a problem? The answer seems to depend on what the goal is and what the alternative is. Since animals live in a natural world, a bias on natural images seem a reasonable, ethologically-relevant prior, and for this purpose ImageNet is among the best available datasets. Indeed, it seems harder to motivate any other prior, including a uniform prior on all possible pixel combinations. However, it is worth pointing out that, should a different prior be needed, XDream can be outfitted with another generator that reflect the chosen prior. (Training such a generator may be challenging, but an entire, active field of generative modeling is dedicated to such problems.)

In sum, we agree that the generators are biased to "natural image statistics" as represented in the dataset in a hard-to-define way, although available data suggest it does not adversely affect search for preferred images.

5) The claim about robustness to noise is overstated. Figure 6 makes the technique look quite susceptible to Poisson noise, depending upon target layer.

Thank you for pointing this out. We originally intended to compare the noise robustness of different optimization algorithms; the header is overly broad and we have changed it. Although the genetic algorithm is more robust than the other two optimization algorithms, it still sensitive to noise to an extent. To characterize the noise sensitivity better, we have collected additional data where we varied the amount of noise. In the new Fig 4b, the optimization results are presented according to amount of stochasticity quantified in intuitive and commonly-used terms (self-correlation, noise as fraction of signal, and signal-to-noise ratio). This can function as a reference table that, based on the empirical amount of noise, helps an investigator better estimate how well XDream can perform.

Minor issues:

1) Figure 1b and 1c: there are 9 minor tick marks and ten example images.

Thank you for catching this. The minor tick at 0 was blocked by a major tick mark. It has been fixed.

2) Figure 2 and 3 appear to be swapped (at least in the pdf I received).

Thank you. It was a mistake when uploading the figures and should now be fixed.

3) Figure 2: label the random ImageNet samples in the Figure (grey boxes). label the dotted line as "maximum ImageNet response" or something similar. This will make the figure easier to read and digest.

4) line 112: ...we qualitative[ly] assessed...

5) lines 136-7: delete either "it can generate" or "can be generated"

6) Figure 4: label the open and solid violin plots on the Figure

3–6) Thank you for the suggestions. We have applied all of them.

7) Table 1: the caption appears to be a Latinate placeholder.

8) lines 211-9: this section needs to be rewritten. There needs to be a reference to Table 1, otherwise "slope" is introduced before the reader has seen such a thing. It isn't in a figure or preceding discussion. One is left to infer what variables the linear regression is being performed upon.

7–8) We have inadvertently placed the table caption in the main text. This is now fixed, and "slope" is also more clearly defined in the table legend.

Reviewer 2: I will upload the pdf with my comments in it, where I have found typos and made wording suggestions.

We have fixed all the typos identified and incorporated the wording suggestions, thank you. We added the Zeiler & Fergus reference. As requested, we added a supplementary figure (new S2 Fig) showing evolved images for intermediate layers for non-AlexNet architectures. There are a few comments in the PDF document for which a written response would be useful:

Regarding the gradient of slopes in Table 1, we added to the main text offering our interpretation (lines 263–266).

A set of recommended hyperparameters had been included in Supplementary Tables S1 and S2.

Regarding the "rather high" mutation rate in Table S1, we note that: 1) mutation rate and size work in conjunction, 2) mutation size is not scale-free but rather depends on the scale of the input space of the generator, which

needs to be measured empirically based on the input training data. We did so for one example generator, DeePSiM-fc6; the input codes during training have standard deviation of approx. 15—in this light, a mutation size of 0.5 is relatively small. In general, rather than manually choosing hyperparameters, we empirically optimize them. An example of how hyperparameter choice matters for XDream performance is shown in what is now S5 Fig.

We switch next to the questions and suggestions that were separate from those in the PDF document (for which, again, we are grateful).

Review of XDream: finding preferred stimuli for visual neurons using generative networks and gradient-free optimization

by Gary Cottrell

This paper uses an existing method for finding optimal visual stimuli for neurons that has previously been applied in Macaque in a paper in Cell in 2019. The goal here is to elucidate how robust the method is by applying it to several deep network models with varying architectures and at different layers of the networks. Using network models allows for extensive experimentation that is impractical in biological preparations. The model proves to be very robust to various parameter regimes, and finds stimuli that drive the neuron much more than any of the over 1 million images in the ImageNet dataset. One of the most interesting findings here is that, using different image generators or different initial conditions, the model finds multiple images that drive the neurons similarly, and these images resemble one another to the human eye. Hence they suggest that there is an optimal image manifold in the latent space of images. Unfortunately, this point was already made in the prior paper with actual monkey visual neurons.

Since the authors postulate that there is an invariance manifold, it would be useful to test this idea by looking at what is generated by a linear interpolation of the codes found from different initial conditions, and how well those interpolated images drive the model neuron. While it is unlikely that the manifold is linear, since the images are similar, they are probably nearby in this space.

We would like to point out that there are many nearby images that can drive the target neurons approximately equally well: During the last generations, there tend to be subtle changes to the image and small changes in the activation values.

Of course, this does not imply the converse, that similarly-activating images are all nearby. To test this, we performed the suggested analysis (thank you for this wonderful idea). We measured model unit responses to linear interpolations between codes optimized from different initial conditions. (Of note, we interpolated in code space and not pixel space. We think this is more reasonable because images quite different on a pixel level can elicit similar responses, e.g., Fig 3c.) The results are added in the new Fig 3b. The manifolds do not appear *linear*, since extrapolating outward from the line segment connecting two strong stimuli results in significant loss in stimuli effectiveness. For early layer (conv2 and conv4) units, extrapolating inward resulted in higher activation than extrapolating outward, showing a tendency for the manifolds to be *convex*. But the convexity is not perfect, since the interpolations are still weaker than the

endpoints (original optimized images). For fc6 and fc8 units, the interpolation midpoint has much weaker activity than either endpoint, suggesting either that the set of strong stimuli is indeed disjoint, or that the invariance manifold has complex, non-convex geometry (meaning that activity-preserving interpolations between two optimal images are *nonlinear*).

To elaborate, we should first specify that our notion of invariance manifold is intended to mean the collection of codes/images that activate a neuron similarly; an equivalent term is a "level set." A level set exists by definition (even if it may only contain one point), but it may be linear (i.e., it is a linear subspace of the image representation space), convex (i.e., points on an interpolation between any two members of the set are also members of the set), connected (i.e., one can travel between any two points in the set while always remaining in the set), or none of these. The geometry of the level set is dependent on both the image representation space (the "code" space) and the target neuron. To take a concrete example, consider so-called "face neurons" in the middle lateral (ML) face patch, and consider a space of faces images defined by "face shape axes" (Chang et al., 2017). The neurons have linear level sets ("invariance manifolds") reflected by their linear tuning function. On the other hand, if we consider the same neurons with regards to pixel-level representation, one can imagine that the "invariance manifolds" will be highly nonlinear.

In a sense, to understand the tuning of a neuron is equivalent to identifying the image representation space in which the neuron has a simple (e.g., linear) "invariance manifold." The fact that we do not find linear or convex manifolds for later layer neurons suggests that, even though the generative networks enables us to find effective stimuli, these networks still do not perfectly match the feature representation of the neurons.

We thank the reviewer for raising this interesting point and summarize the discussion in the main text (lines S345–361).;

In the paragraph on other things one could study with this approach, such as correlated firing, synchronized firing, LFPs, etc., it would be helpful to say what you would optimize in a couple of cases. E.g., you might say, "for example, we could optimize based on increased correlations in firing rates between neurons" or some such.

Thank you. We have followed your suggestion.

The paper could be improved by moving more of the information into the methods section or into supplementary material. There are many details that make the exposition rather tough sledding for the reader. In particular, the section on the effects of different generators has a couple of caveats, e.g., "except for CaffeNet conv2", and "The pixel-based image generator, compared to generative neural networks, worked more poorly in all target layers other than CaffeNet conv2 except when compared to deepsim-norm2 ($p = 1$ compared to deepsim-norm2; $p > 0.14$ compared to other generators in CaffeNet conv2; $p < 10^{-4}$ in all other comparisons; FDR-corrected for 32 tests comparing each generator to raw-pixel in each target layer)." This amount of detail makes my eyes glaze over. I think this section doesn't add a lot to the paper, and could profitably be moved to the supplementary material.

Thank you. To make this section flow better, we have re-written to report the statistics in a clearer fashion. We think this content does add to the results; please see the next reply.

Similarly, the section on different optimizers doesn't flow well. The GA works better on some layers and the other two algorithms work better in some other layers. I'm not sure I care, and I'm not sure what the take-home message is. Again, there are similar "this works better in this layer and that works better in other layers" results in the noise experiments. I think the noise experiments are important, as this is a more realistic case. Unfortunately, the results in Figure 6 are not encouraging. I would disagree with the header for this section: "XDream is robust to noise in neuronal responses." It doesn't seem that robust. I wonder if there is a fix for this - for example, can you average the rate over some time interval of the Poisson process instead of simply sampling from it? Unfortunately, I don't know enough about Poisson processes to know if this is a good suggestion or not.

Thank you for these comments. In terms of practical utility, we agree that the sections on generator and optimizer choice are of limited use, in large part because it turns out that the original choice of generator and optimizer happens to be near-optimal, among the options tested. However, in terms of developing the algorithm, we think these sections are useful for highlighting the modularity of the algorithm, suggesting directions in which it can be improved, and explaining extensions we have tested.

We agree that the original section header is inaccurate (the section was originally meant to compare among optimization algorithms.) and have changed it to "The influence of neuronal noise on XDream." Now, we better characterize the algorithm's ability to tolerate varying amounts of noise; the results are presented in the new Fig 4b.

We consistently use a Poisson noise model, which has a Fano factor (variance-to-mean ratio) of 1, and which has been a typical way to characterize spiking activity in cortex (Gabbiani, 2010, *Mathematics for Neuroscientists*). As discussed in Methods, in this model, the relative amount of noise (standard deviation-to-mean ratio, which is closely related to signal-to-noise ratio) will decrease with more spikes observed—this could result from a higher firing rate, longer observation time, and/or several repetitions, as the reviewer suggested. To account for these factors, we varied the "expected max number of spikes"—that is, we normalized all responses by the expected max activation in 2,500 natural images, scaled the responses by a varying number, then used that as the rate of a Poisson process (Methods). This procedure results in different levels of noise. We further quantify the amount of noise with several commonly used measures: trial-to-trial correlation, standard deviation-to-mean ratio, and signal-to-noise ratio. The results can be used like a look-up-table to map experimentally observed neuronal noisiness to how XDream is expected to perform. In (what is now) Fig 4a, the "Noisy" condition corresponds to expecting 20 spikes from the best image—for example, a firing rate of 100 spks/s and a 200 ms observation window. The spike number can be higher (and effective noise lower) when using a longer observation window, multi-unit activity, and/or more image repetitions.

The number of repetitions is considered separately. Since we impose a strict limit of 10,000 image *presentations*, more repetition means fewer unique images shown and consequently fewer optimization iterations. This trade-off has been considered during parameter optimization (now S5 Fig, last panel); for the genetic algorithm, the final performance decreases when the number of repetitions is greater than 1, at least with the original noise level (20 spikes expected in response to a preferred image). It is possible that with even noisier data, more repetitions could be beneficial, although this would make optimization harder in general.

One concern is that, although the authors state that "we focus on the more biologically relevant scenario where there is no information about the architecture and weights of the target model, and where we only have access to a few, potentially stochastic, activation values from the neurons." In fact, they don't have access to only a few activations. The model is used to generate 10,000 images to find the optimal one. This seems biologically unrealistic. I skimmed the previous paper, and there they used many fewer generations for the monkeys - 200. Since this is what is apparently possible in biological preparations, it seems like they should evaluate how much is gained in 200 generations and compare that to the 500 generations they used here. This would provide a better estimate of what is possible today.

The main limitation in biological recordings is time, which maps directly to the number of "queries" (image presentations). The number of queries is therefore an important concern for us, motivating our strict limit of 10,000 image presentations throughout the paper. This number is quite realistic. For example, in Ponce et al., 2019, each generation consisted of 40 synthetic and 40 reference images, which for 200 generations corresponds to $40 \times 40 \times 200 = 16,000$ image presentations (of which 8,000 were used for evolution). What's more, in this paper the population size (images per generation) is 20 rather than 40, making it unfair to directly compare the number of generations.

Still, 10,000 is just an arbitrary number. To gauge how much optimization can be achieved over time, take Fig 1b as an example: Activation grows roughly logarithmically with generation, and most of the optimization gain is achieved in the first 250 generations (activation of around 60 by generation 250 *vs.* around 70 by 500).

Originality

The originality is tempered somewhat by the fact that this is investigating an existing method that has already been used in Macaque visual cortex. Obviously, this is different in the sense that using convnets as the preparation allows for much more extensive experimentation with the method than would be possible with a biological one, which is the point of this paper. For example, they can test the neuron's response to the over one million images in ImageNet, and then compare the results of this brute force search to the effectiveness of XDream. They vary just about everything and still find that the method works well. So it is original in that sense, but I have to think that the big-font headline is the first paper.

We are glad that the reviewer agrees with our motivation for the paper.

Several neurophysiologists are now interested in this type of methods to study neural responses. We hope that the current paper will justify parameter choice and better describe performance under conditions that cannot quite be tested experimentally for practical reasons, as the reviewer points out.

Innovation

Again, the innovation here is to benchmark their method using in silico models. This kind of thing has been done before with other analyses of deep net features.

We are not entirely sure which "other analyses"the reviewer is referring to. Perhaps the reviewer is referring to DeepDream and similar studies where investigators visualize unit preferences. However, we emphasize that DeepDream and similar algorithms strictly depend on completely knowledge of the system's weights and architecture. There have also been studies that apply neurophysiological methods to explain deep nets (Pospisil et al., 2018, eLife); the chief goal in those studies is not to use deep nets to benchmark/validate a method. As the reviewer points out, our main goal here is to connect with the Neuroscience literature and help experimentalists who want to study neural coding for visual features in cortex.

High importance to researchers in the field:

To the extent to which other researchers may start to use this method, the importance here is that neuroscientists can get some assurance that the method is robust, and that they needn't worry too much about optimizing the meta-parameters.

Indeed, we agree with the reviewer.

"Similar activation values notwithstanding, the optimized images were different on a pixel level (Fig 4b); they may comprise an "invariance manifold" for each neuron that contains similar but not identical images eliciting comparable activation values (see Discussion)." I think this is an important point, so it would be good to test the hypothesis by looking along a line between some of the image codes to see if there are optimal images between these. This would be of interest to researchers using this method.

Thank you for this suggestion. As detailed above, we have tested this idea and reported the results.

Significant biological and/or methodological insight:

There is not much in the way of biological insight here, but the paper does demonstrate that this methodology is robust.

Indeed, the focus here is on the method itself. On a speculative note, several works have argued that the ConvNets that we test here constitute an approximation to visual cortex (e.g., Yamins & Dicarlo 2014 among many others). To the extent that visual cortex can be modeled by a ConvNets (a matter of significant debate in the field), the conclusions drawn here could extrapolate to visual cortex. However, the reviewer correctly points out that the main focus here is on the method.

Rigorous methodology

This is quite a rigorous test of the model.

Substantial evidence for its conclusions

The evidence that this approach works well over a variety of convnet architectures and layers is extensive. On the other hand, I think the evidence for robustness to noise is weak. This is a point the authors should address in a revision of the paper.

We have revised this part as suggested (see above).

Minor comments:

I'm not quite sure what this sentence means: "the standard deviation was lower than 10% of the activation values (Fig 4a)."

This means that if a different *random* initialization is used, on average, one would expect no more than a 10% change in the activation value. We have made the relevant figure and description clearer.

I'm not sure the slopes in Table 1 give a very intuitive idea of how much improvement you get by using good vs. bad initializations. A bar chart might be better. Also, while the text suggests that there isn't much difference, the slopes seem fairly big as you go deeper in the net using the ivt method. I don't know if that conclusion of mine is warranted. I.e., I would better understand this if the actual differences in medians were shown instead of the slope. Perhaps another violin plot would work here? It seems slightly counterintuitive to conclude that there is only slight variation in the optimized image activation while the p-values for the differences are on the order of 10^{-77}.

We apologize that the legend for this table was misplaced into the main text, making the table hard to interpret. This has now been fixed. Regarding the suggesting of a bar chart/violin plot, the effect of using different initializations are indeed reported, as a violin plot, in (what is now) Fig 3d.

The slope was intended as a more specific statistical test (because it specifies the direction of change) than ANOVA. We report p-values for the slope (Table 1) and ANOVA (main text). To be sure, there is an effect of using a *good* initialization—provided the investigator knows, *a priori*, a preferred image comparable to the best in approx. 1 M images (a strong assumption). Nevertheless, this is consistent with the conclusion that there is no effect when using different *random* initializations and, indeed, when using middle or worst ImageNet images. These issues are discussed in the last two paragraphs in the main text, section "XDream is robust to different initial conditions."

Manuscript Click here to access/download;Manuscript;main2.pdf

XDream: finding preferred stimuli for visual neurons using generative networks and gradient-free optimization

Will Xiao[1,2,*], Gabriel Kreiman[2,3],

1 Department of Molecular and Cellular Biology, Harvard University, Cambridge, MA, U.S.A.
2 Center for Brains, Minds, and Machines, Boston, MA, U.S.A.
3 Department of Ophthalmology, Boston Children's Hospital, Boston, MA, U.S.A

* xiaow@fas.harvard.edu

Short title : Finding preferred visual stimuli via optimization

Abstract

A longstanding question in sensory neuroscience is what types of stimuli drive neurons to fire. The characterization of effective stimuli has traditionally been based on a combination of intuition, insights from previous studies, and luck. A new method termed XDream (E**X**tending **D**eepDream with **r**eal-time **e**volution for **a**ctivation **m**aximization) combined a generative neural network and a genetic algorithm in a closed loop to create strong stimuli for neurons in the macaque visual cortex [1]. Here we extensively and systematically evaluate the performance of XDream. We use ConvNet units as *in silico* models of neurons, enabling experiments that would be prohibitive with biological neurons. We evaluated how the method compares to brute-force search, and how well the method generalizes to different neurons and processing stages. We also explored design and parameter choices. XDream can efficiently find preferred features for visual units without any prior knowledge about those units. XDream extrapolates to different layers, architectures, and training datasets, performing better than brute-force search, and often better than exhaustive sampling of >1 million images. Furthermore, XDream is robust to choices of multiple image generators, optimization algorithms, and hyperparameters, suggesting that its performance is locally near-optimal. Lastly, we found no significant advantage to problem-specific parameter tuning. These results establish expectations and provide practical recommendations for using XDream to investigate neural coding in biological preparations. Overall, XDream is an efficient, general, and robust algorithm for uncovering neuronal tuning preferences using a vast and diverse stimulus space. XDream is implemented in Python, released under the MIT License, and works on Linux, Windows, and MacOS.

Introduction

What stimuli excite a neuron, and how can we find them? Consider vision as a paradigmatic example: The selection of stimuli to probe neural

activity has shaped the understanding of how visual neurons represent information. It is practically impossible to exhaustively evaluate neuronal responses to images, due to the combinatorially large number of possible images. Instead, investigators have traditionally selected stimuli guided by natural image statistics, behavioral relevance, theoretical postulates about internal representations, intuitions from previous studies, and serendipitous findings. Stimuli selected in this way underlie our current understandings of how circular center-surround receptive fields [2] give rise to orientation tuning [3], then to encoding of more complex shapes such as curvatures [4, 5], and further to selective responses to complex objects such as faces [6–8].

Despite the progress made in understanding visual cortex by testing limited sets of hand-chosen stimuli, these experiments could be missing the true feature preferences of neurons. In other words, there could be other stimuli that drive visual neurons better than stimuli found so far. Such stimuli could lead us to revisit our current descriptions of feature tuning in visual cortex.

A recently introduced method shows promise to begin bridging the gap. Named XDream (e**X**tending **D**eepDream with **r**eal-time **e**volution for **a**ctivation **m**aximization), this method combines a genetic algorithm and a deep generative neural network [9]—both inspired by previous work [10–13]—to evolve images that trigger high activation in neurons [1]. XDream can generate strong stimuli for neurons in macaque inferior temporal (IT) and primary visual cortex (V1).

The performance and design options of XDream have not been thoroughly evaluated, due to the time-intensive nature of neuronal recordings and the difficulty to fully control experimental variables. To overcome these challenges, here we test the performance of XDream using state-of-the-art *in silico* models of visual neurons in lieu of real neurons, in the same spirit of [14]. Specifically, we use convolutional neural networks (ConvNets) pre-trained on visual recognition tasks as an approximation to the computations performed along ventral visual cortex [15–17]. Using these models as a proxy for real neurons allows us to compare synthetic stimuli with a large set of reference images, to evaluate XDream's performance across processing stages, model architectures, and training regimes, to empirically optimize algorithm design and parameter choices in a systematic fashion, and to disentangle the effects of neuronal response stochasticity.

Although there is a rich literature in computer science on feature visualization [18–21], we focus on the more biologically relevant scenario where there is no information about the architecture and weights of the target model, and where we only have access to a few, potentially stochastic, activation values from the neurons. These conditions reflect those prevailing in neuronal recordings and are fundamentally different from the assumptions made in computer science studies.

Under these realistic constraints, we show that XDream still reliably and efficiently uncovers preferred features of units with a wide range of response properties, generalizing to different processing stages within a network, different network architectures, and different training datasets. Furthermore, XDream performed equally well with a wide range of algorithmic and parameter choices. Based on these results, we suggest parameters to use and results that can be expected when using XDream to investigate neuronal tuning properties. Our findings indicate that XDream

is a general and robust method for investigating neuronal preferences in visual cortex.

Design and Implementation

Overview

Move to the end of the sentence

XDream combines in a closed loop an image generator (e.g., the generator in a generative adversarial network), a target neuron (e.g., a unit in a ConvNet), and a non-gradient-based optimization algorithm (e.g., a genetic algorithm). In each iteration, the optimization algorithm proposes a set of codes, the image generator synthesizes the codes into images, the images are evaluated by the target neuron to produce one scalar score per image, and the scores are used by the optimization algorithm to propose a new set of codes (Fig. 1). Importantly, no optimization gradient is needed from the neuron.

The image generators and optimization algorithms are detailed below. The code is implemented in Python 3 and runs on Linux, Windows, and MacOS, although the former two platforms are required to use GPU acceleration. The main dependency is Caffe [22] (`https://caffe.berkeleyvision.org/`) or PyTorch (`https://pytorch.org/`), which are required for neural network computation. Other dependencies are standard Python packages and listed in requirements.txt in the repository, including: numpy, h5py, opencv-python, scipy, and scikit-image.

Image generators

An image generator is a function that outputs an image given some representation of that image (an *image code*) as input. We tested the family of DeePSiM generators developed in [9]. DeePSiM generators are generative adversarial networks trained to invert each layer of AlexNet [23]. The pre-trained models are available at `https://lmb.informatik.uni-freiburg.de/people/dosovits/code.html`. We have converted the models into PyTorch for convenience for future research. Links to the converted models are available in the code repository (see Code availability below). We used the image generator inverting the fc6 layer by default except in S4 Fig, where we compared different generators. An alternative version of the DeePSiM-fc6 generator was trained on the Places-365 dataset using code from [9] and a pre-trained classifier [24].

Fitness function

The key metric XDream optimizes is a scalar value we refer to as *fitness*, which is associated with each image. In the neuroscience context, a fitness function can be the stimulus-evoked spike count for a neuron in visual cortex. In the current study, the fitness function is the activation of the target unit in a ConvNet.

Optimization algorithms

An optimization algorithm in the context of XDream is a function that iteratively proposes a set of n image codes (real-valued vectors) or *codes*

for short, $\boldsymbol{c}_i, i = 1, \ldots, n$, and then uses their corresponding *fitness* values $y_i, i = 1, \ldots, n$ to propose a new set of codes expected to have higher fitness. We used a genetic algorithm by default, but also considered two other algorithms: finite-difference gradient descent (FDGD) and natural evolution strategies [25] (NES). Implementation details for the optimization algorithms are available in the Supplementary Material.

Computing environment

Neural network computations were performed on NVIDIA GPUs. Portions of this research were conducted on the O2 High Performance Compute Cluster, supported by the Research Computing Group, at Harvard Medical School. See `http://rc.hms.harvard.edu` for more information.

delete highlighted comma

Results

Random exploration of stimulus space is inefficient

A common approach for exploring neuronal selectivity is to use arbitrarily selected images, often from a limited number of categories (for example in [8, 26]). Thus, we considered random exploration as a baseline for comparison. We used the AlexNet architecture as the target model [23] (implemented as CaffeNet; S1 Table) and sampled images from ImageNet [27] (ILSVRC12 dataset, 1,431,167 images), a large dataset common in computer vision that also contains the training set of CaffeNet. We randomly sampled n images either from all of ImageNet or from 10 categories randomly selected from the 1,000 training categories in ImageNet ($n/10$ images per category). We evaluated the activation values in response to these images for units in different layers of the network, and calculated the *relative activation*, defined as the ratio between the activations to the n random images and the maximum activation in all of ImageNet. By definition, the relative activation for the best image in ImageNet is 1, which is also an upper bound on the observed relative activation values when using random sampling. Randomly selected images typically yielded relative activation values well below 1 (S1 Fig). As expected, the maximum observed relative activation increased with n but only did so slowly, with near-logarithmic growth. Moreover, for later layers (e.g., fc8), sampling from only 10 categories yielded significantly worse results than sampling completely randomly, which we hypothesize is because the small number of categories imposes a bottleneck on the diversity of high-level features represented. In neuroscience studies, category selection is clearly not completely random: Investigators may have intuitions and prior knowledge about the types of stimuli that are more likely to be effective. To the extent that those intuitions are correct, such priors can enhance the search process. However, those intuitions are seldom guided by systematic examination of stimulus space and could well miss important types of stimuli.

XDream can find strong stimuli for neurons

XDream has three key components: an image generator representing the search space; an objective function given by the activation of the target unit guiding the search; and an optimization algorithm performing the

Fig 1. Overview of the XDream method. a), XDream combines an image generator, a target neuron, and a non-gradient-based optimization algorithm. **b,c)**, An example experiment targeting CaffeNet layer fc8, unit 1. **b)**, Mean activation achieved over 500 generations, 20 images per generation (10,000 total image presentations). **c)**, Images obtained at a few example generations indicated by minor x-ticks in **b)**. The activation to each image is labeled above the image and indicated by the color of the margin. **d)**, The top 5 images among 10,000 random images from ImageNet (ILSVRC12 dataset, $>$ 1.4 M images). The number of random images is matched to the number of images presented during optimization. The top image in all $>$ 1.4 M images is shown in Fig 2b.

search (Fig 1a). In each generation, the generator creates images from their latent representations (*codes*), the target unit activation is evaluated for each of the generated images, and the optimizer refines the codes based on the activation values. The algorithm is initialized randomly (examples shown in Fig 1a) and iterated for 10,000 total image presentations. We chose this number because it is relatively small and accessible in a typical neuroscience experiment [1]. Critically, the algorithm does not use any prior knowledge about the architecture or weights of the target model.

consider replacing with "Crucially"

An example experiment with unit 1 in the output layer (layer fc8) of CaffeNet is shown in Fig 1b,c. In 500 generations of 20 images each, the activation of the target unit increased rapidly and saturated at approximately generation 300. Fig 1c shows example images at a few generations (log-spaced to show a range of activations), illustrating the evolution of the images from the initial noise pattern to the final image. In the following analyses, we concentrate on the best image in the last generation, which we refer to as the *optimized image*. However, it is worth noting that responses to all the 10,000 unique images during the evolution may illuminate features of the neuron's tuning (see Discussion).

How strong was the activation achieved by XDream-generated images? We compared the optimized image to images from ImageNet. Unit 1 in layer fc8 was trained to be a "goldfish" detector. Correspondingly, when we randomly sampled 10,000 images from ImageNet, the best images were photos of goldfish (Fig 1d). The highest activation value observed in this random sample was 30.67. The best image from ImageNet for this unit was a picture of a goldfish and elicited an activation of 40.55 (Fig 2b). Consistent with S1 Fig, the best image found by random sampling produced a much lower activation value than the best example in ImageNet. In comparison, the optimized image generated by XDream elicited an activation of 72.42. In other words, using a limited number of presentations, XDream generated images that elicited higher activation than any natural image from ImageNet. We refer to such images with relative activation $>$ 1 as *super stimuli*.

XDream generalizes across layers, architectures, and training sets

The default generative network used in XDream was trained to invert the internal representations at layer fc6 of CaffeNet, which was in turn trained on ImageNet [9]. Can this generator allow XDream to generalize to other network layers, architectures, and training sets? If XDream is specific to

Fig 2. XDream generalizes across layers, architectures, and training sets. a), Violin plot showing the distributions of relative activation (activation of optimized stimulus relative to highest activation in > 1.4 M ImageNet images) over 100 randomly selected units per layer. For each target model, we investigated early, middle, late, and output layers (see S1 Table for the specific layers). The violin contours indicate kernel density estimates of the distributions, white circles indicate the medians, thick bars indicate first and third quartiles, and whiskers indicate $1.5\times$ interquartile ranges. For comparison, grey boxes (interquartile ranges) and lines (medians) show the distribution of maximum relative activation for 10,000 random ImageNet images. The horizontal dashed line corresponds to the best ImageNet image. **b)**, Optimized (top row) and best ImageNet (bottom row) images and activations for 10 example units across layers and architectures. For output units, corresponding category labels are shown below the images.

certain layers and architectures, or specific to ImageNet-trained networks, this may limit its applicability to real neurons.

We first assessed whether XDream could extrapolate to other layers in CaffeNet by selecting 100 units respectively from the early, middle, late, and output layers of CaffeNet (Fig 2a). XDream was able to find optimized images that are better than the best randomly selected images across all layers ($p < 10^{-16}$, false discovery rate (FDR) corrected for 28 tests in this section). The optimized images were also significantly better than the best images in ImageNet ($p < 10^{-9}$, FDR corrected).

Next, we tested 100 units from each of 4 layers from 5 different network architectures: ResNet-v2 152- and 269-layer variants [28], Inception-v3 [29], Inception-v4, and Inception-ResNet-v2 [30]. These models were all trained on ImageNet. XDream was able to generate better images than random exploration across all layers and architectures (Fig 2a; $p < 10^{-8}$ across layers) except the early layer of Inception-v3 ($p = 0.2$) and of Inception-ResNet-v2 ($p = 0.09$). With the same exceptions, XDream generated super stimuli for all other tested layers ($p = 0.01$ for the early layer of Inception-v4, $p = 2 \times 10^{-4}$ for the middle layer of Inception-ResNet-v2, and $p < 10^{-9}$ for all other layers). Example optimized images for units in different layers and architectures are shown in Fig 2b and S2 Fig. Furthermore, several generators trained on different layer representations performed equally well across classifier layers (S4 Fig).

Finally, we tested the ability of XDream to optimize unit responses when the generator and target networks are trained on different datasets. We tested PlacesCNN [31], a network with the same architecture as CaffeNet but trained on the MIT Places dataset. The Places dataset also contains photographic images, but they mainly depict scenes rather than objects. Again, XDream was able to find super stimuli across all layers in this network (Fig 2a, last four distributions; $p < 10^{-6}$ across layers), even when using a generative network trained on different images. Conversely, when using a generator trained on the Places dataset, XDream still performed similarly well in optimizing CaffeNet and PlacesCNN (S4 Fig).

These results show that XDream can efficiently create images that trigger high activations in a target unit without making assumptions about the type of images that unit may prefer and without any knowledge of the target model architecture. This indicates that XDream may well be

applicable to biological neurons. Furthermore, XDream generalizes across layers in a ConvNet, while different layers roughly correspond to areas along the ventral visual stream [17, 32, 33]. Thus, XDream may also generalize to several ventral stream areas. Consistent with this observation, results from [1] indicated that XDream can find optimized stimuli for V1 and inferior temporal cortex (IT) neurons.

XDream is robust to different initial conditions

XDream starts the search from an initial generation of image codes. In Fig 2, we always initialized the algorithm using the same set of 20 random image codes, 6 of which are shown in Fig 1a. Does the choice of initial conditions affect the results?

To address this question, we first tested how much the particular set of random initial codes matters. For each target unit, we repeated the experiment using 10 ~~othermore~~ different random initializations and compared the optimized relative activation to that of the original random initialization. Different initial conditions produced slightly better or worse relative activation values centered around a mean difference of 0, and the standard deviation of the fractional change was lower than 10% (Fig 3a).

remove "othermore"

Similar activation values notwithstanding, the optimized images were different on a pixel level (Fig 3c). These images may comprise an "invariance manifold" that contains similar, but not identical, images eliciting comparable activation values. What might this invariance manifold look like? To explore this question, in CaffeNet layers conv2, conv4, fc6, and fc8, we linearly interpolated between two separately optimized images (from different initializations), and measured target unit activation in response to the interpolated images (Fig 3b). The interpolated images were much stronger stimuli compared to the majority of ImageNet images. However, particularly in layers fc6 and fc8, the interpolation midpoint has weaker activity than either endpoint, suggesting either that the sets of strong stimuli are disjoint, or that the invariance manifold may have non-convex geometry.

David Sheinberg has some data that supports this - he found a cell in macaque that responded to both car images and butterfly images, with the strongest response to a particular car and a particular butterfly.

Fig 3. Comparison of different initializations. a,b,c), Effect of using different random initializations. **a)**, Distributions of fractional change in optimized activation if 10 different random initializations are used. **b)**, Left, relative activation in response to interpolated images between two optimized images from two different random initial conditions. Right, activation normalized to the endpoints (location 0 or 1), highlighting the change in activation away from the endpoints. **c)**, Optimized images from different initializations for 3 example units in the output layer (one unit per row). Activation values are shown above each image. **d)**, Good versus bad initializations. For each target unit, its best, middle, or worst 20 images from ImageNet were used as the initial generation. The images were converted to the image code space using either an optimization method ("opt") or an inversion method ("ivt"; Methods). Left to right within the opt and ivt groups are results from initialization with the best, middle, and worst 20 images. Random initialization is shown for comparison. The open and solid violins show the distributions, in the first and last generation respectively, of relative activation over 100 units in each layer.

You ordered them here opposite of the way you did in the initial manuscript, so it is worst, middle, and best.

Next, we tested whether there are particularly good or bad ways of

I don't see how Figure 3c shows this? And Table 1 shows the slope again, without any explanation in the text!

Ok, it looks like you intended for the text under Table 1 to be a "caption": but Tables don't have captions! Please move that into the main text.

Anyway, don't reference Figure 3c for this. I think you mean Figure 3d?

choosing the initial stimuli. We selected, separately for each target unit, the 20 ImageNet images that led to the highest, middle, and lowest activation values and used those images to form the initial population (Fig 3c). To convert images into image codes comprising the initial population, we used either the "opt" or the "ivt" algorithm (Methods). Initializing with better or worse natural images did not improve the optimized images in the conv2 layer ($p = 0.87$ and 0.19 for "opt" and "ivt," respectively, FDR-corrected for 8 tests in this and the next sentence). In higher layers, initializing with the best natural images led to slightly higher relative activation values (Fig 3c; Table 1; $p < 5 \times 10^{-3}$ for "opt" and $p < 10^{-10}$ for "ivt" across layers). We speculate that the improvement in higher layers is because units in deeper layers are progressively more selective, making it more difficult to optimize their responses. Therefore, more optimal initializations are beneficial. However, in an actual neurophysiology experiment, it is unlikely that the investigator would know, *a priori*, such good stimuli as the best of 1.4 M images. Excluding the best natural images, initializing with the middle natural images, worst natural images, or random images codes were not significantly different ($p \geq 0.15$, FDR-corrected for 8 tests) in any layer with the "opt" method. With the "ivt" method, these initializations worked similarly in layers conv2 and conv4, not in layers fc6 ($p = 0.0014$) and fc8 ($p = 2 \times 10^{-15}$). However, in those layers, using random initialization was better ($p < 10^{-8}$, FDR-corrected for 4 tests). Therefore, initializing randomly appears to be a reasonable choice in general practical scenarios.

Better than what? They aren't better than the best

Table 1. Effect of using good vs. bad initialization.

Encoding alg.	Measure	Layer conv2	conv4	fc6	fc8
opt	slope	0.010	0.037	0.047	0.056
	p-value	0.87	0.004	0.004	7×10^{-5}
ivt	slope	0.044	0.113	0.241	0.353
	p-value	0.19	7×10^{-11}	4×10^{-22}	5×10^{-77}

How did you calculate the slope? I assume you used linear regression over the three (0,1,2). Just say that.

For each unit, the 20 best, middle, and worst images from ImageNet, as ranked by that unit, were used to initialize the genetic algorithm. The images were converted to image codes using one of two encoding algorithms, "opt" or "ivt" (see Methods). The slope was calculated for relative activation (median across 100 random units each layer) as a function of the initialization, with {0,1,2} for {worst, middle, best}, respectively. This slope quantifies the improvement in relative activation when a better initialization is used (worst → middle or middle → best).

To summarize, initializing the algorithm with different random conditions resulted in only a small variation in the optimized image activation, and the optimized images were similar, although not identical, at the pixel level. Initializing with prior knowledge has little to no effect on the optimized image activation, unless the seed is comparable to the best image in ~ 1 M images and only in later layers.

Different optimization algorithms can be incorporated into XDream, but the genetic algorithm consistently works well

An important component of XDream is the optimization algorithm. The results shown thus far were based on using a genetic algorithm as the optimization algorithm, a choice inspired by previous work [10–12]. Here, we compared the genetic algorithm to two additional algorithms, a naïve finite-difference gradient descent algorithm (FDGD; Methods) and Natural Evolution Strategies (NES; [25], Methods). NES has been used in a related problem [34]. FDGD and NES were significantly worse than the genetic algorithm in CaffeNet conv2 ($p < 10^{-13}$, FDR corrected for 20 tests here and in the next section) and conv4 layers ($p < 10^{-3}$), but significantly better than the genetic algorithm in CaffeNet fc6 ($p < 10^{-16}$), fc8 ($p < 10^{-16}$), and Inception-ResNet-v2 classifier layers ($p < 10^{-12}$; Fig 4a).

Why do you think that is?

Fig 4. Comparison of optimization algorithms and their robustness to noise. a), Three gradient-free optimization algorithms were evaluated for their ability to find preferred images and tolerate noise: a genetic algorithm, finite-difference gradient descent (FDGD), and Natural Evolution Strategies (NES; [25]). Left and right half of each violin, respectively, correspond to noiseless and noisy units. Format of the plot is as in Fig S4 Figb. **b)**, The performance of the genetic algorithm gradually improves with decreasing amounts of noise within a neurophysiologically relevant range. Format of the plot is as in Fig 2a except that the violins are horizontal.

The influence of neuronal noise on XDream

An important difference between model units and real neurons is the lack of noise in model unit activations. In response to the same image, a model unit returns a deterministic activation value. In contrast, for biological neurons, the same image can evoke different responses on repeated presentations (even though the responses may be highly consistent when averaged across trials; see [35]). To test whether XDream could still find super stimuli with noisy units, we used a simple model of neuronal noise: We used the true activation value to control the rate of a homogeneous Poisson process, from which the "observed" activation value on a single trial was drawn (Methods). Homogeneous Poisson processes have been used extensively to model stochasticity in cortical neurons [36].

As expected, performance deteriorated when noise was added (Fig 4a, noisy condition). However, XDream using the genetic algorithm was still able to find optimized stimuli better than random exploration for most layers ($p < 10^{-10}$ for all tested layers except $p = 0.19$ for CaffeNet fc8, FDR-corrected for 5 tests). XDream was also able to find super stimuli for some layers ($p < 10^{-5}$ for CaffeNet conv4 and fc6 layers; FDR-corrected for 5 tests).

This is a big difference between hidden layers and output. Any idea why? Especially since you optimized your hyperparameters for the final layer, if I understand your methods correctly.

Noisy unit activations affected different optimization algorithms to different extents. The genetic algorithm was at least as good as, and often superior to, both alternative optimization algorithms when considering noisy units. The NES algorithm performed ~~similarly well as the genetic~~ algorithm in CaffeNet fc8 layer and Inception-ResNet-v2 classifier layer

performed similarly to the genetic…

($p = 0.03$ and 0.65, respectively), but was worse in the other 3 tested layers ($p < 10^{-14}$). The FDGD algorithm was particularly sensitive to noise, performing worse than the genetic algorithm in all layers tested ($p < 10^{-6}$) and frequently failing to find good stimuli.

In the noisy ~~condition~~ conditions examined thus far, we assumed that in each presentation, model units yielded approximately 20 spikes for a "good" stimulus (defined as the expected best image in 2,500 random ImageNet images). This choice was motivated by what may be realistically expected when recording from biological neurons (e.g., firing rate of 100 spikes per second to a good stimulus over a 200 ms observation window), but this number will be dependent on individual neurons and specific experimental designs. This number matters because, for a homogeneous Poisson process, its standard deviation-to-mean ratio is inversely proportional to the square root of the rate parameter (average expected number of spikes), and thus a higher firing rate means a higher signal-to-noise ratio. To characterize the performance of XDream under different noise conditions, we varied the rate parameter as defined by the expected max spike number and measured XDream performance on the different noise levels (Fig 4b). The empirical level of noise was quantified with commonly used measures such as trial-to-trial self-correlation, standard deviation-to-mean ratio, and signal-to-noise ratio (SNR). As the amount of noise decreased, the performance of XDream gradually approached its noiseless performance. Notably, even with a high level of noise (5 spikes for a good stimulus, self-correlation of 0.08, and SNR of 2), XDream was able to find super stimuli for around half of the target units in all but the deepest layer (fc8) tested.

Availability and Future Directions

The code for XDream can be obtained directly from `https://github.com/willwx/XDream/`.

In the computer science literature, activation maximization is a well-known approach for visualizing features represented by units in a ConvNet [13, 21, 37–39]. However, the techniques are only applicable to networks that provide optimization gradients. In other words, perfect knowledge is assumed of the target network architecture and weights. Clearly, such requirements are not met in current neuroscience experiments.

Several recent studies have focused on the problem of finding preferred stimuli for biological neurons, and introduced a different approach [32, 33, 40, 41]: A ConvNet-based model is first fitted to predict neuronal responses to a set of training images. Then, standard white-box activation maximization techniques are applied ~~on~~ to the ConvNet model. The relation between this approach and XDream is similar to the relation between the so-called "substitute model" method and what, in comparison, we may call a "direct" method, in research on black-box adversarial attacks. A promising future direction is to combine the two approaches to leverage their unique advantages: unlimited queries (after training) and efficient optimization with substitute models; avoiding model extrapolation and transferability problems with direct optimization.

The results presented here are based on maximizing activation values, whereas the results obtained in visual cortical neurons in [1] are based on maximizing spike counts. Activation values and firing rates are

commonly-used proxies for internal representation in machine learning and neuroscience, respectively. However, other putative neural codes can be studied. For example, one could maximize pooled activation across multiple units, increase sparseness of the representation across units, match a pre-specified pattern of population firing, increase correlation or synchrony in firing of nearby units, maximize power in a certain frequency band in local field potentials, and so on. XDream is agnostic to the underpinning of the objective function as long as it is image-specific, quantitatively defined, and computable in real time. Thus, the same algorithm can be readily applied to investigate different putative neural coding mechanisms.

Finally, it is worth remembering that the identification of an optimal stimulus, or even a diverse set of them, still does not automatically lead to a full characterization of the function of a neuron. Finding preferred stimuli, or "feature visualization" in computer science parlance, has guided thinking about the function of individual neurons in both neuroscience and deep learning [7, 21]. However, optimal stimuli reflect but do not disentangle critical issues like tuning features, invariant features, and context dependence; these questions need to be distinguished by subsequent hypothesis-driven investigation [42–44]. A method to automatically find preferred stimuli of neurons can suggest initial hypotheses about a poorly-understood visual area, or motivate re-thinking about an extensively-studied region. Of note, during the optimization process, XDream does test thousands of related images, covering the target unit's response levels both widely and densely (Fig 1c). Closer analyses of these images may reveal richer information about the tuning surface of a neuron (e.g., invariances) than what is reflected by the single best image.

In summary, XDream is able to discover preferred stimuli of visual neurons without assuming any knowledge about the structure or connectivity of the system under study. Thus, XDream can be a powerful tool for elucidating the tuning properties of neurons in a variety of visual areas in different species, even where there is no prior knowledge about the neuronal preferences. The general framework of XDream may potentially be applicable to other sensory domains, such as sounds, language, and music, as long as good generative models can be built.

Acknowledgements

This work was supported by the Center for Brains, Minds and Machines funded by NSF STC award CCF-1231216 and also by NIH R01EY026025. Portions of this research were conducted on the O2 High Performance Compute Cluster, supported by the Research Computing Group, at Harvard Medical School. See `http://rc.hms.harvard.edu` for more information. The authors declare no competing interests.

References

1. Ponce CR, Xiao W, Schade P, Hartmann TS, Kreiman G, Livingstone MS. Evolving Images for Visual Neurons Using a Deep Generative Network Reveals Coding Principles and Neuronal Preferences. Cell. 2019;177:999–1009. doi:10.1016/j.cell.2019.04.005.

2. Kuffler S. Discharge patterns and functional organization of mammalian retina. Journal of Neurophysiology. 1953;16:37–68.

3. Hubel DH, Wiesel TN. Receptive fields, binocular interaction and functional architecture in the cat's visual cortex. The Journal of physiology. 1962;160(1):106–154.

4. Gallant JL, Braun J, Van Essen DC. Selectivity for polar, hyperbolic, and Cartesian gratings in macaque visual cortex. Science. 1993;259(5091):100–103.

5. Pasupathy A, Connor CE. Population coding of shape in area V4. Nature Neuroscience. 2002;5(12):1332–1338. doi:10.1038/972.

6. Logothetis NK, Sheinberg DL. Visual object recognition. Annual Review of Neuroscience. 1996;19:577–621. doi:10.1146/annurev.ne.19.030196.003045.

7. Desimone R, Albright T, Gross C, Bruce C. Stimulus-selective properties of inferior temporal neurons in the macaque. Journal of Neuroscience. 1984;4(8):2051–2062.

8. Tsao DY, Freiwald WA, Tootell RBH, Livingstone MS. A Cortical Region Consisting Entirely of Face-Selective Cells. Science. 2006;311(5761):670–674. doi:10.1126/science.1119983.

9. Dosovitskiy A, Brox T. Generating Images with Perceptual Similarity Metrics based on Deep Networks. In: Advances in Neural Information Processing Systems; 2016. p. 658–666.

10. Yamane Y, Carlson ET, Bowman KC, Wang Z, Connor CE. A neural code for three-dimensional object shape in macaque inferotemporal cortex. Nature Neuroscience. 2008;11(11):1352–1360. doi:10.1038/nn.2202.

11. Carlson ET, Rasquinha RJ, Zhang K, Connor CE. A Sparse Object Coding Scheme in Area V4. Current Biology. 2011;21(4):288–293. doi:10.1016/j.cub.2011.01.013.

12. Vaziri S, Carlson ET, Wang Z, Connor CE. A Channel for 3D Environmental Shape in Anterior Inferotemporal Cortex. Neuron. 2014;84(1):55–62. doi:10.1016/j.neuron.2014.08.043.

13. Nguyen A, Dosovitskiy A, Yosinski J, Brox T, Clune J. Synthesizing the preferred inputs for neurons in neural networks via deep generator networks. In: Advances in Neural Information Processing Systems; 2016. p. 3387–3395.

14. Pospisil DA, Pasupathy A, Bair W. 'Artiphysiology' reveals V4-like shape tuning in a deep network trained for image classification. eLife. 2018;7:e38242.

15. Yamins DLK, Hong H, Cadieu CF, Solomon EA, Seibert D, DiCarlo JJ. Performance-optimized hierarchical models predict neural responses in higher visual cortex. Proceedings of the National Academy of Sciences. 2014;111(23):8619–8624. doi:10.1073/pnas.1403112111.

16. Schrimpf M, Kubilius J, Hong H, Majaj NJ, Rajalingham R, Issa EB, et al. Brain-Score: Which Artificial Neural Network for Object Recognition is most Brain-Like? bioRxiv. 2018;.

17. Cadena SA, Denfield GH, Walker EY, Gatys LA, Tolias AS, Bethge M, et al. Deep convolutional models improve predictions of macaque V1 responses to natural images. PLOS Computational Biology. 2019;15(4):1–27. doi:10.1371/journal.pcbi.1006897.

18. Erhan D, Bengio Y, Courville A, Vincent P. Visualizing Higher-Layer Features of a Deep Network. University of Montreal. 2009;1341(3).

19. Zeiler MD, Fergus R. Visualizing and Understanding Convolutional Networks. In: European conference on computer vision. Springer; 2014. p. 818–833.

20. Szegedy C, Zaremba W, Sutskever I, Bruna J, Erhan D, Goodfellow I, et al. Intriguing properties of neural networks. arXiv. 2013;.

21. Olah C, Mordvintsev A, Schubert L. Feature Visualization. Distill. 2017;.

22. Jia Y, Shelhamer E, Donahue J, Karayev S, Long J, Girshick R, et al. Caffe: Convolutional Architecture for Fast Feature Embedding. arXiv. 2014;.

23. Krizhevsky A, Sutskever I, Hinton GE. ImageNet Classification with Deep Convolutional Neural Networks. In: Pereira F, Burges CJC, Bottou L, Weinberger KQ, editors. Advances in Neural Information Processing Systems 25; 2012. p. 1097–1105.

24. Zhou B, Lapedriza A, Khosla A, Oliva A, Torralba A. Places: A 10 million Image Database for Scene Recognition. IEEE Transactions on Pattern Analysis and Machine Intelligence. 2017;40(6):1452–1464.

25. Wierstra D, Schaul T, Glasmachers T, Sun Y, Peters J, Schmidhuber J. Natural Evolution Strategies. Journal of Machine Learning Research. 2014;15:949–980.

26. Liu H, Agam Y, Madsen J, Kreiman G. Timing, timing, timing: Fast decoding of object information from intracranial field potentials in human visual cortex. Neuron. 2009;62:281–290.

27. Russakovsky O, Deng J, Su H, Krause J, Satheesh S, Ma S, et al. ImageNet Large Scale Visual Recognition Challenge. International Journal of Computer Vision. 2015;115(3):211–252. doi:10.1007/s11263-015-0816-y.

28. He K, Zhang X, Ren S, Sun J. Identity Mappings in Deep Residual Networks. Lecture Notes in Computer Science. 2016; p. 630–645. doi:10.1007/978-3-319-46493-0_38.

29. Szegedy C, Vanhoucke V, Ioffe S, Shlens J, Wojna Z. Rethinking the Inception architecture for computer vision. In: Proceedings of the IEEE conference on computer vision and pattern recognition; 2016. p. 2818–2826.

30. Szegedy C, Ioffe S, Vanhoucke V, Alemi AA. Inception-v4, Inception-ResNet and the Impact of Residual Connections on Learning. In: Thirty-First AAAI Conference on Artificial Intelligence; 2017. p. 4278–4284.

31. Zhou B, Lapedriza A, Xiao J, Torralba A, Oliva A. Learning Deep Features for Scene Recognition using Places Database. In: Advances in Neural Information Processing Systems 29; 2014. p. 487–495.

32. Bashivan P, Kar K, DiCarlo JJ. Neural population control via deep image synthesis. Science. 2019;364(6439). doi:10.1126/science.aav9436.

33. Abbasi-Asl R, Chen Y, Bloniarz A, Oliver M, Willmore BDB, Gallant JL, et al. The DeepTune framework for modeling and characterizing neurons in visual cortex area V4. bioRxiv. 2018;.

34. Ilyas A, Engstrom L, Athalye A, Lin J. Black-box Adversarial Attacks with Limited Queries and Information. In: Dy J, Krause A, editors. Proceedings of the 35th International Conference on Machine Learning. vol. 80 of Proceedings of Machine Learning Research; 2018. p. 2137–2146.

35. Koch C. Biophysics of Computation. New York: Oxford University Press; 1999.

36. Gabbiani F, Cox S. Mathematics for Neuroscientists. London: Academic Press; 2010.

37. Simonyan K, Vedaldi A, Zisserman A. Deep Inside Convolutional Networks: Visualising Image Classification Models and Saliency Maps. arXiv. 2013;.

38. Olah C, Satyanarayan A, Johnson I, Carter S, Schubert L, Ye K, et al. The Building Blocks of Interpretability. Distill. 2018;.

39. Carter S, Armstrong Z, Schubert L, Johnson I, Olah C. Activation Atlas. Distill. 2019;.

40. Walker EY, Sinz FH, Froudarakis E, Fahey PG, Muhammad T, Ecker AS, et al. Inception in visual cortex: in vivo-silico loops reveal most exciting images. bioRxiv. 2018;.

41. Malakhova K. Visualization of information encoded by neurons in the higher-level areas of the visual system. J Opt Technol. 2018;85(8):494–498. doi:10.1364/JOT.85.000494.

42. Kobatake E, Tanaka K. Neuronal selectivities to complex object features in the ventral visual pathway of the macaque cerebral cortex. Journal of Neurophysiology. 1994;71:856–867.

43. Chang L, Tsao DY. The Code for Facial Identity in the Primate Brain. Cell. 2017;169:1013–1028.e14. doi:10.1016/j.cell.2017.05.011.

44. Geirhos R, Rubisch P, Michaelis C, Bethge M, Wichmann FA, Brendel W. ImageNet-trained CNNs are biased towards texture; increasing shape bias improves accuracy and robustness. arXiv. 2018;.

Fig 1

Click here to access/download;Figure;Figure 1.tif

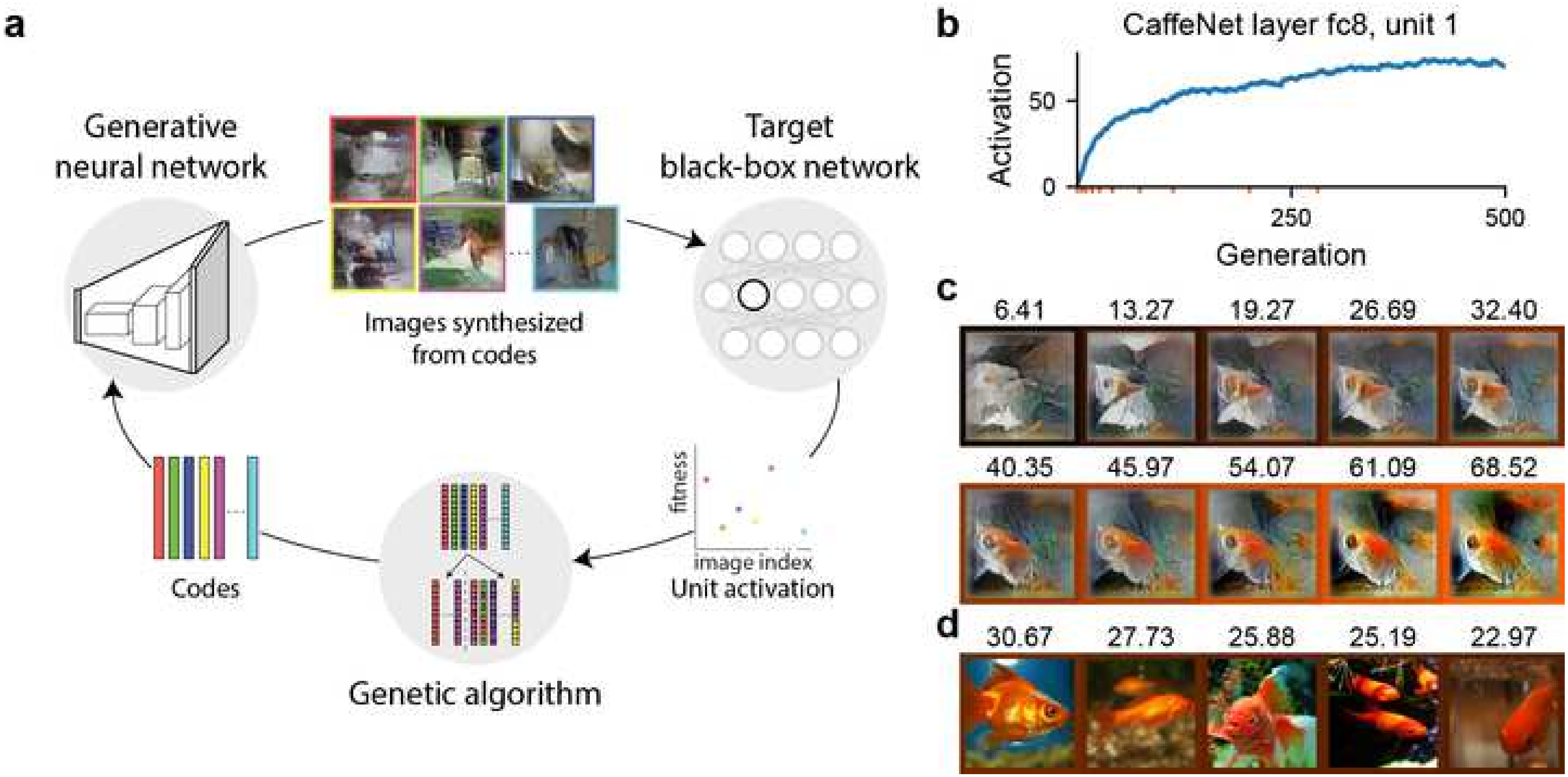

Fig 2

Click here to access/download;Figure;Figure 2.tif

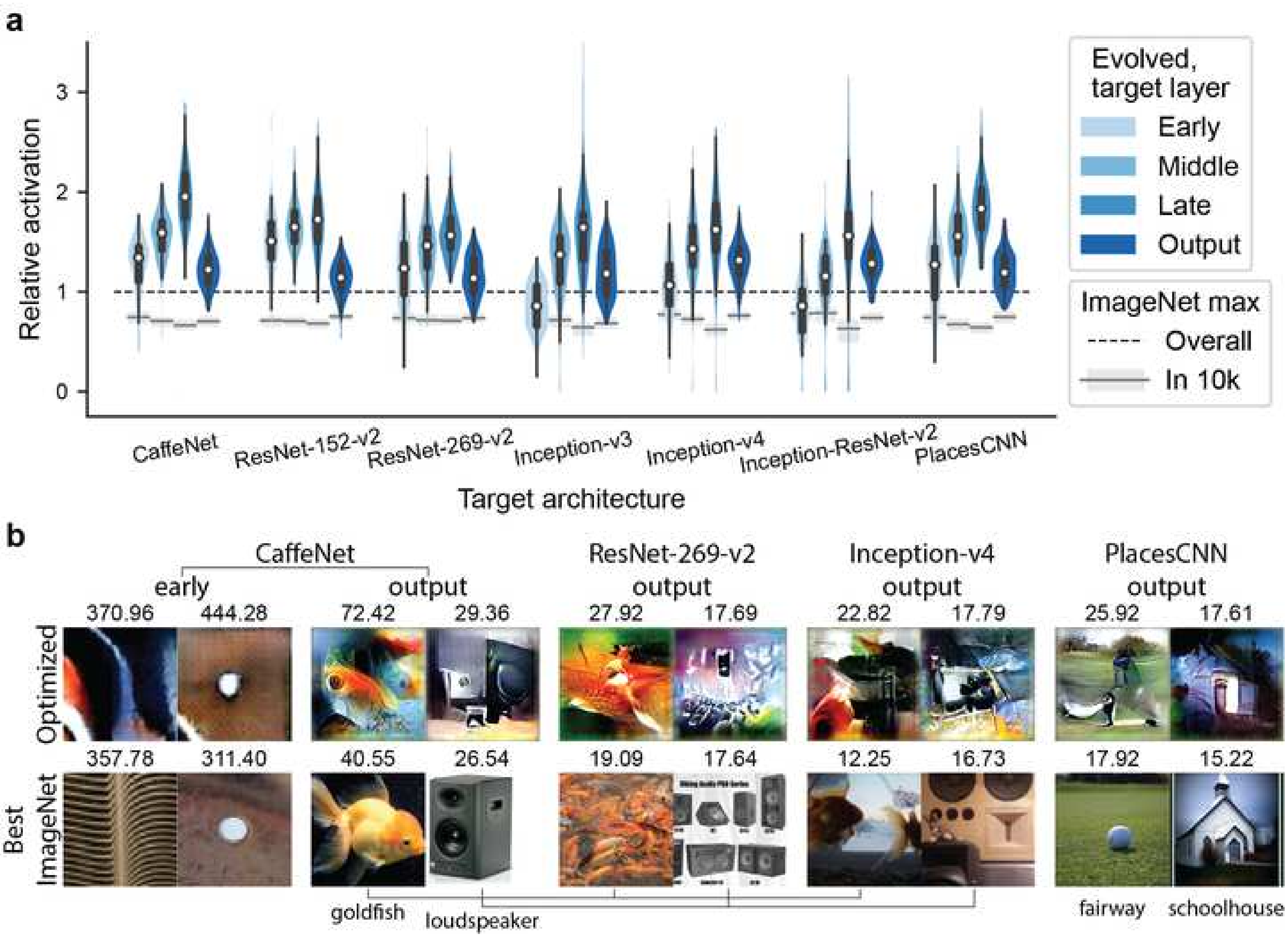

Fig 3

Click here to access/download;Figure;Figure 3.tif

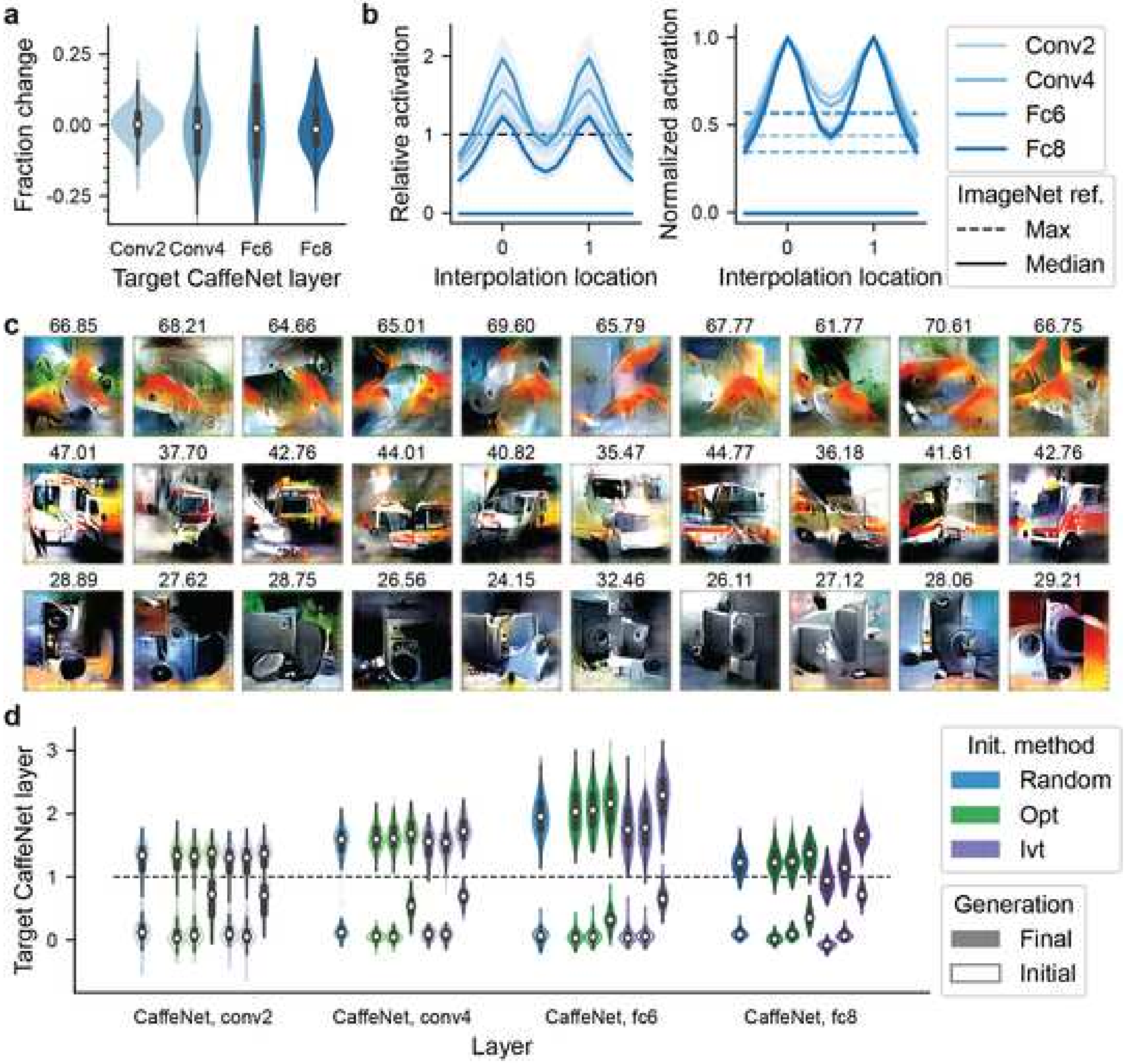

Fig 4 Click here to access/download;Figure;Figure 4.tif

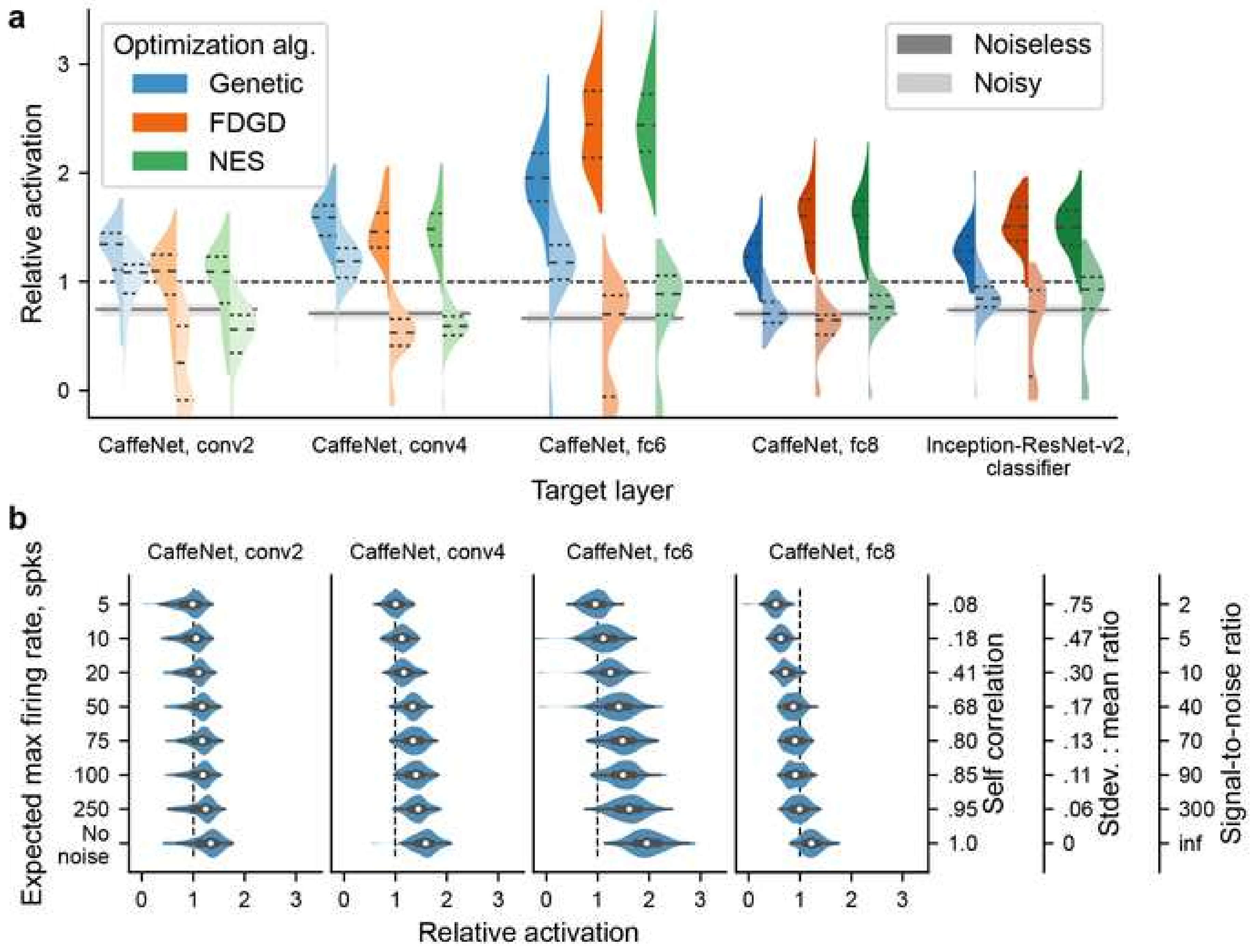

Click here to access/download
Supporting Information
suppl2.pdf

S1 Fig

Click here to access/download
Supporting Information
S1 Fig.tif

S2 Fig

Click here to access/download
Supporting Information
S2 Fig.tif

S3 Fig

Click here to access/download
Supporting Information
S3 Fig.tif

S4 Fig

Click here to access/download
Supporting Information
S4 Fig.tif

S5 Fig

Click here to access/download
Supporting Information
S5 Fig.tif

S6 Fig

Click here to access/download
Supporting Information
S6 Fig.tif

Code to Deposit

Click here to access/download

Supporting Information - Compressed/ZIP File Archive

XDream-update_1.zip

Manuscript with Tracked Changes

Click here to access/download
Related Manuscript
main2-changes.pdf

Click here to access/download
Related Manuscript
main1-changes.pdf

Data Review URL

Click here to download Data Review URL
http://github.com/willwx/XDream/tree/update_1
