# Supplementary material for: XDream: Finding preferred stimuli for visual neurons using generative networks and gradient-free optimization
Source: PLoS Comput Biol. 2020 Jun 15;16(6):e1007973. doi: 10.1371/journal.pcbi.1007973 (PMC7316361; doi:10.1371/journal.pcbi.1007973)
Supplement: Attachment — Submitted filename: simon_selectivity.pdf [file pcbi.1007973.s015.pdf]

Cell Si002 – Response to "Diagnostic Cue" (Color) Stimuli

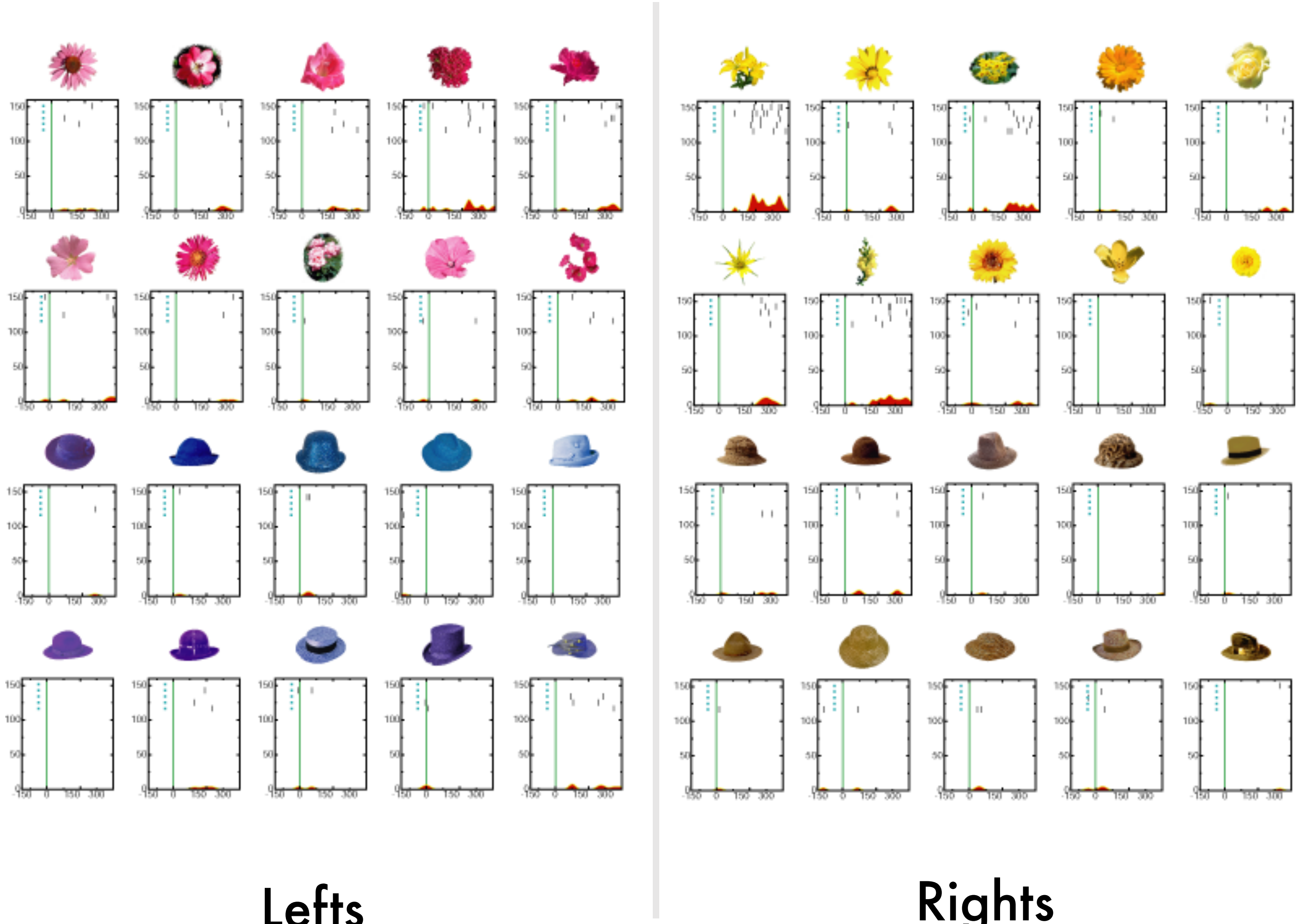

Lefts

Rights

Cell Si002 – Response to "Diagnostic Cue" (Color) Stimuli

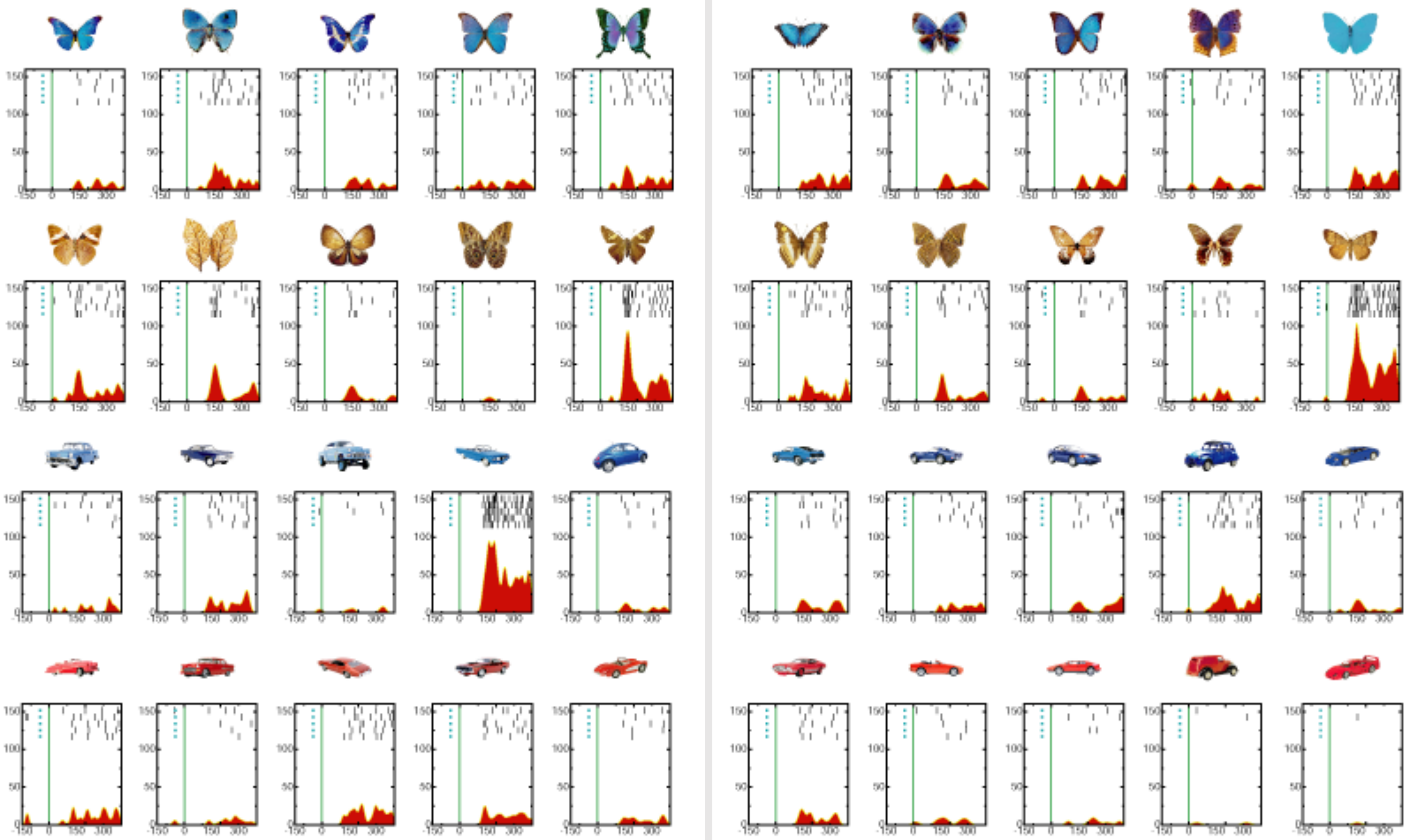

Lefts

Rights
