# Supplementary material for: XDream: Finding preferred stimuli for visual neurons using generative networks and gradient-free optimization
Source: PLoS Comput Biol. 2020 Jun 15;16(6):e1007973. doi: 10.1371/journal.pcbi.1007973 (PMC7316361; doi:10.1371/journal.pcbi.1007973)
Supplement: Attachment — Submitted filename: response.pdf [file pcbi.1007973.s016.pdf]

Reviewer #2: Review of Xdream revision 1.

The reviewers have adequately addressed most of my comments, so this version is improved over the previous version, but there are still a few things that need to be addressed, and unfortunately, on the second reading, I have a couple of new questions for you.

I will also upload my marked-up version of the pdf for wording and typos I found.

I should say I only skimmed the supplementary material, but I did have one comment on the discussion: In the second paragraph, you refer to "the direct method", which is not mentioned in the previous paragraph. You need to reintroduce this idea here, as a neuro person will not have any idea what you are talking about without going back to the main article.

In the paper, you linearly interpolate between the optimized images. In the response, you mention that you don't do this in pixel space, but in the latent space. You should mention that here.

Thank you very much for these comments, which clarify and improve the writing. Now, the term "direct method" is briefly introduced in the supplement, and the reader is also referred to the main text. We have specified the interpolation space in the main text and figure legend, as suggested.

BTW, David Sheinberg showed some data at CNS in 2003 that may be relevant here. He told me it didn't make it into a paper, but he found a cell in macaque that responded to both car images and butterfly images, with the strongest response to a particular car and a particular butterfly - very disjoint stimuli! It would be cool to stick that in here somewhere; I'll upload the data with my review. I should mention that these images were very well known to the monkey.

Thank you for bringing up this illustrative result, which deepens thinking about seemingly disjoint stimulus preferences. In our results (Fig. 3c), independently evolved images for *in silico* units do share (subjective) semblance, which is somewhat different from the data you shared from David Sheinberg. As the reviewer points out, the observation of seemingly unrelated feature preference is not uncommon (in our personal discussions with neurophysiologists). We have added a general description in the main text:

"Studies have reported visual neurons that prefer seemingly unrelated stimuli. It remains an interesting open question to identify whether there exists a feature representation space in which neuronal tuning functions have "simple" geometry."

There's a typo on page 8/14, line 262, where you refer to Figure 3c, but I think you mean Figure 3d. On that same page, you again mention Table 1, without explanation. The explanation does appear in a caption under table (you should also mention the way you calculated the slope - I assume linear regression). Tables don't have captions, so you will still need to move this into the main text, presumably before you mention the table.

Thank you for the suggestions. We have fixed the panel reference. We now specify that the slope is calculated via linear regression in the table legend, which appears to be allowed in this journal (https://journals.plos.org/ploscompbiol/s/tables).

The table addresses the same point (effect of using good vs. bad initialization) as the sentence and the cited figure panel (Fig. 3d) immediately preceding it, so we did not add a dedicated explanation for the table.

Line 272 page 8: With the "ivt" method, these initializations worked similarly in layers conv2 and conv4, not in layers fc6 ($p = 0.0014$) and fc8 ($p = 2 \times 10^{-15}$). This doesn't say how they worked in layers fc6 anbd fc8 (again, these details are boring and irrelevant, since, as you say, PIs are not likely to have optimal images to start with. I still recommend leaving them to the supplementary material). But I'm not even sure what you are saying here. What are you referring to? Is it that opt is better than ivt in these layers? When I looked at the figure, I thought you were referring to how the best initialization gave bigger improvements here over medium and worst initializations for these layers. Please clarify this.

Likewise, the next sentence says that random initializations worked better, but you don't say better than what, and it sure doesn't look like they are better than the other results in Figure 3d, at least for medium and bad initializations.

Again, this paragraph doesn't flow well. The message you want to get across is: In a realistic situation, where the PI doesn't have access to optimal starting images, random initialization works about as well as anything else. Start by saying that starting with the optimal image does help in some cases, but given that investigators are not likely to have access to these, using random initialization is sufficient. Details in the supplementary material.

Thank you for the questions and suggestion. We agree that this discussion is of marginal interest in practice and, rather than belabor it, we have removed the two sentences you pointed out as confusing. To address your specific questions, by "worked similarly" we refer to the results of ANOVA tests, which test for *whether* conditions are different, not *how* they are different; the latter requires separate tests for each specific possibility. We did perform such tests (one tailed Wilcoxon signed-rank test) in the next sentence to compare random initialization to initializing with the middle or the worst stimuli (specified in the text: "Excluding the best natural images..."). Random initialization is statistically significantly better. This significant difference may not be evident upon visualization of the violin plot since the Wilcoxon test is pairwise (i.e., it compares two conditions for each unit individually), whereas the violin plot (and ANOVA) compares on the level of the whole population of 100 units, and hence is less sensitive.

Figure 3d has some issues. In the caption, you give the order as best, middle, worst, but you've reversed the order from the previous manuscript - it's now worst, middle, best. Also, the y-axis caption is incorrect - it should be "Relative Activation", not "Target CaffeNet layer."

Thank you. We have fixed the typos.

Figure 4B has four different y-axis labels (!). This is not explained in the figure caption (or the text), so you may as well leave the extra three out, as they are confusing and a distraction from the main point.

Thank you for the suggestion. We realize this is an unconventional format of plotting. Now, we have de-emphasized the additional axes by grouping them

separately, in a lighter color and with smaller font size. We kept the additional y-axes because they do serve an essential purpose, namely to allow investigators to relate their experimental condition to the simulation conditions we used. This has been explained in the main text.

Page 9: optimization methods: There are some significant differences between the algorithms. Can you comment on why you think that is?

Thank you for the question. This is an empirical finding, for which we do not have a strong hypothesis. Further experiments are likely required for dissecting why certain optimizers work bettern in certain cases.

Page 9: noise: There is a big difference of the effect of noise on the algorithm in the hidden layers versus the output. Any idea why? This seems odd, given that you've optimized your metaparameters on the output neurons, if I understood the methods correctly.

Thank you for the observation. We suspect that the larger effect of noise in the output layer is because the output layer, unlike hidden layers, is specifically trained to be sparse due to the categorization task. Therefore, we speculate that the optimization gradient in output layers is shallow in most of image space, making the optimization harder than in other layers and especially susceptible to noise.
